# The heterogeneity of attenuated and brief limited psychotic symptoms: association of contents with age, sex, country, religion, comorbidities, and functioning

**DOI:** 10.3389/fpsyt.2023.1209485

**Published:** 2023-07-07

**Authors:** Christian Theisen, Marlene Rosen, Eva Meisenzahl, Nikolaos Koutsouleris, Theresa Lichtenstein, Stephan Ruhrmann, Joseph Kambeitz, Lana Kambeitz-Ilankovic, Anita Riecher-Rössler, Katharine Chisholm, Rachel Upthegrove, Linda A. Antonucci, Alessandro Bertolino, Alessandro Pigoni, Raimo K. R. Salokangas, Christos Pantelis, Stephen J. Wood, Rebekka Lencer, Peter Falkai, Jarmo Hietala, Paolo Brambilla, André Schmidt, Christina Andreou, Stefan Borgwardt, Naweed Osman, Frauke Schultze-Lutter

**Affiliations:** ^1^Department of Psychiatry and Psychotherapy, Medical Faculty, Heinrich-Heine University, Düsseldorf, Germany; ^2^Department of Psychiatry and Psychotherapy, Faculty of Medicine and University Hospital Cologne, University of Cologne, Cologne, Germany; ^3^Department of Psychiatry and Psychotherapy, Ludwig-Maximilian-University, Munich, Germany; ^4^Max-Planck-Institute of Psychiatry, Munich, Germany; ^5^Institute of Psychiatry, Psychology and Neuroscience, King's College London, London, United Kingdom; ^6^Research Center Jülich, Institute for Cognitive Neuroscience (INM-3), Jülich, Germany; ^7^Medical Faculty, University of Basel, Basel, Switzerland; ^8^Institute for Mental Health, University of Birmingham, Birmingham, United Kingdom; ^9^Department of Psychology, Aston University, Birmingham, United Kingdom; ^10^Department of Basic Medical Science, Neuroscience, and Sense Organs, University of Bari Aldo Moro, Bari, Italy; ^11^Department of Neurosciences and Mental Health, Fondazione IRCCS Ca' Granda Ospedale Maggiore Policlinico, Milan, Italy; ^12^MoMiLab Research Unit, IMT School for Advanced Studies Lucca, Lucca, Italy; ^13^Department of Psychiatry, Medical Faculty, University of Turku, Turku, Finland; ^14^Melbourne Neuropsychiatry Centre, University of Melbourne, Melbourne, VIC, Australia; ^15^Orygen, The National Centre of Excellence for Youth Mental Health, Melbourne, VIC, Australia; ^16^Centre for Youth Mental Health, University of Melbourne, Melbourne, VIC, Australia; ^17^Department of Psychiatry and Psychotherapy, Otto Creutzfeldt Center for Cognitive and Behavioral Neuroscience, University of Muenster, Muenster, Germany; ^18^Department of Psychiatry and Psychotherapy, University of Lübeck, Lübeck, Germany; ^19^Department of Pathophysiology and Mental Health, University of Milan, Milan, Italy; ^20^Department of Psychiatry, University of Basel, Basel, Switzerland; ^21^Department of Psychology, Faculty of Psychology, Airlangga University, Surabaya, Indonesia; ^22^University Hospital of Child and Adolescent Psychiatry and Psychotherapy, University of Bern, Bern, Switzerland

**Keywords:** Attenuated Psychotic Symptoms, delusional ideas, hallucinatory experiences, disorganized communication, clinical-high risk for psychosis

## Abstract

**Introduction:**

The Attenuated Psychosis Symptoms (APS) syndrome mostly represents the ultra-high-risk state of psychosis but, as does the Brief Intermittent Psychotic Symptoms (BIPS) syndrome, shows a large variance in conversion rates. This may be due to the heterogeneity of APS/BIPS that may be related to the effects of culture, sex, age, and other psychiatric morbidities. Thus, we investigated the different thematic contents of APS and their association with sex, age, country, religion, comorbidity, and functioning to gain a better understanding of the psychosis-risk syndrome.

**Method:**

A sample of 232 clinical high-risk subjects according to the ultra-high risk and basic symptom criteria was recruited as part of a European study conducted in Germany, Italy, Switzerland, and Finland. Case vignettes, originally used for supervision of inclusion criteria, were investigated for APS/BIPS contents, which were compared for sex, age, country, religion, functioning, and comorbidities using chi-squared tests and regression analyses.

**Result:**

We extracted 109 different contents, mainly of APS (96.8%): 63 delusional, 29 hallucinatory, and 17 speech-disorganized contents. Only 20 contents (18.3%) were present in at least 5% of the sample, with paranoid and referential ideas being the most frequent. Thirty-one (28.5%) contents, in particular, bizarre ideas and perceptual abnormalities, demonstrated an association with age, country, comorbidity, or functioning, with regression models of country and obsessive-compulsive disorders explaining most of the variance: 55.8 and 38.3%, respectively. Contents did not differ between religious groups.

**Conclusion:**

Psychosis-risk patients report a wide range of different contents of APS/BIPS, underlining the psychopathological heterogeneity of this group but also revealing a potential core set of contents. Compared to earlier reports on North-American samples, our maximum prevalence rates of contents were considerably lower; this likely being related to a stricter rating of APS/BIPS and cultural influences, in particular, higher schizotypy reported in North-America. The various associations of some APS/BIPS contents with country, age, comorbidities, and functioning might moderate their clinical severity and, consequently, the related risk for psychosis and/or persistent functional disability.

## 1. Introduction

Psychotic disorders are associated with high cost and burden (1–3) and lead to a reduced life expectancy of almost 12 years (4). Only 13.5% of all schizophrenia patients met the criteria for recovery despite advances in treatment (5). Thus, there is an urgent need for prevention (6). Because the first episode of psychotic disorders is mostly preceded by a prodromal phase of several years on average during which functional deficits already develop, an indicated prevention targeting help-seeking persons with the first signs of the emerging disorder has been considered most feasible (7). To identify persons with an increased risk of going on to develop a first psychotic episode among the help-seekers, clinical high-risk criteria (CHR) were developed and validated within the past three decades (7). Thereby, two complementary approaches were followed: the Ultra-High-Risk (UHR) and the basic symptom criteria (8) (note: CHR is used as an umbrella term when referring to both the UHR and the basic symptom approach). UHR criteria consist of the Attenuated Psychotic Symptoms (APS) syndrome, the Brief Intermittent Psychotic Symptoms (BIPS) syndrome, and the Genetic Risk and Functional Deterioration (GRFD) syndrome (9). Basic symptom criteria comprise the COGnitive-PERceptive basic symptoms (COPER) and the COGnitive DISturbance (COGDIS) (10). Of these five single criteria, the APS and BIPS syndromes and COGDIS were recommended for CHR detection (8).

On average, 85% of persons recruited into UHR samples meet the APS syndrome (11) and showed significant heterogeneity in conversion rates (8, 11). Among others, this may be due to the heterogeneity of APS, which integrate a wide range of different symptoms (12), different recruitment strategies and, relatedly, epidemiological filters (13), cultural effects (14, 15), childhood adversities and trauma (16), as well as effects of age or sex on both clinical significance and psychosis-predictive value of APS (17–20). APS/BIPS is identified in semi-structured interviews that rate APS/BIPS syndromally (21). In doing so, the various unusual thought contents, perceptual abnormalities, and types of conceptual disorganization that constitute APS/BIPS are generally assessed in separate aggregated items that distinguish unusual thought contents either by their bizarreness or by their general content, i.e., paranoid, grandiose, or other ideas (22). Even without considering the frequency of the individual symptoms or APS/BIPS contents, which are summarized in the positive items of UHR assessments, severity and prevalence rates of the different positive items commonly greatly differ, with grandiose ideas and conceptual disorganization often reported as least frequent or pronounced (17, 23–25). This heterogeneity of positive items may be partly caused by age and sex. With regard to sex, in clinical samples, more conceptual disorganization (17, 26) and grandiose ideas (17) were found in male participants, whereas female participants showed more severe (27, 28) or more frequent perceptual aberrations (29). This was partly supported by a community study that reported more delusional and perceptual APS in female participants, and a trend significance toward more frequent conceptual disorganization in males participants. Other studies, however, did not show any sex differences in positive items of UHR assessments (30–34). Furthermore, both community and CHR studies reported age effects on the prevalence and clinical relevance—in terms of an association with non-psychotic mental disorders and/or functioning—of APS, with higher frequency and fluctuation of perceptual APS and lesser clinical relevance of delusional APS in younger age groups, in particular those below the age of 16 years (35–38).

First studies on the prevalence rates of APS/BIPS contents in UHR individuals of the North American Prodrome Longitudinal Study 2 (NAPLS-2) (39), conducted in several states of the United States of America (US) and Canada, and a US undergraduate sample (40) using categories predefined in the Content of Attenuated Positive Symptoms (CAPS) codebook (41) indicated that the heterogeneity of contents of APS/BIPS by far exceeds heterogeneity in positive items of UHR assessments. Yet, age and sex effects have not been studied so far with regard to detailed APS/BIPS contents, and neither has the impact of country, religion, functioning, and comorbidities on APS/BIPS contents been studied in UHR samples.

Therefore, our study aimed to detail the contents of APS and BIPS and their prevalence within a large CHR sample of the European Personalized Prognostic Tools for Early Psychosis Management (PRONIA) study (42); (https://www.pronia.eu). Furthermore, to detect what clinical or sociodemographic variable might be related most to the heterogeneity of contents, we examined the cross-sectional association of contents with sex, age, country, religion, psychiatric comorbidity, and functioning to gain a better understanding of the clinical presentation of UHR patients and, hereby, to better target intervention for current APS/BIPS. Based on the above studies, we expected that perceptual APS/BIPS contents would be more frequent in younger and/or female participants, and grandiose contents and signs of conceptual disorganization more frequent in male participants. Furthermore, for the consistent findings of age effects (36–39) and the inconsistent findings of sex effects on APS/BIPS (17, 26–35), we expected that age would be more related to contents than sex.

## 2. Materials and methods

### 2.1. Sample

The CHR sample (*N* = 232) was recruited as part of the PRONIA study, which was funded by a grant from the European Commission and carried out at ten early detection centers in Germany, Italy, England, Finland, and Switzerland between 02/2014 and 11/2018 (42, 43). CHR patients had to meet the following inclusion criteria: age between 15 and 40 years, meeting at least one of the three UHR criteria and/or the basic symptom criterion COGDIS, language skills sufficient for participation, and sufficient capacity to consent/assent.

Participants with a past or present diagnosis of a manifest psychotic disorder, an antipsychotic medication for more than 30 days (cumulative number of days) at or above minimum dosage of the “1st episode psychosis” range of the S3 Guidelines Schizophrenia of the *Deutsche Gesellschaft für Psychiatrie und Psychotherapie, Psychosomatik und Nervenheilkunde* (DGPPN; German Association for Psychiatry, Psychotherapy, and Psychosomatics) (44) (Supplementary Table 1), or any intake of antipsychotic medication (i.e., independent of the duration of intake) within the past 3 months before psychopathological baseline assessments at or above minimum dosage of the “1st episode psychosis” range of DGPPN S3 Guidelines (Supplementary Table 1) were excluded. Furthermore, an intelligence quotient below 70, not sufficient hearing for neurocognitive testing, current or past head trauma with loss of consciousness for more than 5 min, current or past known neurological disorder of the brain, current or past known somatic disorder potentially affecting the structure or functioning of the brain, current or past alcohol dependency, current polytoxicomania (poly-dependency) or polytoxicomania (poly-dependency) within the past 6 months, and the inability to undergo a magnetic resonance imaging for medical or personal reasons were exclusion criteria of the study.

Because APS/BIPS can also occur outside samples meeting the frequency and course requirements of the symptomatic UHR criteria (25, 45), all CHR patients were considered in analyses (Table 1).

**Table 1:** Sociodemographic and clinical characteristics of the sample.

	**Female (*n =* 127; 54.7%)**	**Male (*n =* 105; 45.3%)**	**Total sample (*N =* 232)**	**Statistics**
Age median (mean ± SD)	21 (23.0 ± 5.3)	22 (23.6 ± 5.3)	22 (23.2 ± 5.3)	U = 6,109.5, *p =* 0.271; r=0.072
Education years median (mean ± SD)	13 (13.9 ± 2.4)	13 (13.3 ± 2.7)	13 (13.6 ± 2.6)	U = 5,868.0, *p =* 0.113; r=0.104
**Country** ***n*** **(%)**				χ^2^_(4)_ = **18.242**, ***p** =* **0.001; V** = **0.280**
Germany	74 (58.3%)	49 (46.7%)	123 (53.0%)	
England	13 (10.2%)	6 (5.7%)	19 (8.2%)	
Switzerland	3 (2.4%)	18 (17.1%)	21 (9.0 %)	
Finland	18 (14.2%)	11 (10.5%)	29 (12.5 %)	
Italy	19 (15.0%)	21 (20.0)	40 (17.2 %)	
**Religion** ***n*** **(%)**				χ^2^_(4)_ = **6.526**, ***p** =* **0.163; V** = **0.168**
Atheists	47 (37.0%)	46 (43.8%)	93 (40.0%)	
Christians	66 (52.0%)	55 (52.4%)	121 (52.1%)	
Other (55.6% Muslims)	14 (11.0%)	4 (3.8%)	18 (7.8%)	
**Marital status** ***n*** **(%)**				χ^2^_(3)_ = **5.215**, ***p** =* **0.157; V** = **0.150**
Single	86 (67.7%)	83 (79.0%)	169 (72.8%)	
Married	6 (4.7%)	1 (1.0%)	7 (3.0%)	
Steady partnership	34 (26.8%)	20 (19.0%)	54 (23.3 %)	
Divorced	1 (0.8%)	1 (0.8%)	2 (0.9%)	
**Occupation** ***n*** **(%)**				χ^2^_(1)_ = **19.202**, ***p** =* **0.024; V** = **0.288**
Full-time (incl. in school/training, full-time house-keeper)	79 (62.2%)	64 (60.9%)	143 (61.6%)	
Part-time (incl. others)	11 (4.7%)	8 (7.6%)	19 (8.2%)	
Unemployed/unable to work	37 (29.1%)	33 (31.4%)	70 (30.2%)	
**Current mental disorder** ***n*** **(%)**				
Any disorders	99 (78.0%)	72 (68.6%)	171 (73.7%)	χ^2^_(1)_ = 2.2610, *p =* 0.134; V=0.106^*^
Mood disorder	68 (53.5%)	49 (46.7%)	117 (50.4%)	χ^2^_(1)_ = 1.087, *p =* 0.356; V=0.068^*^
Anxiety disorder	49 (38.6%)	23 (21.9%)	72 (31.0%)	χ^2^_(1)_ = 7.470, *p =* 0.007; V=0.179^*^
Obsessive-compulsive disorder	12 (9.4%)	8 (7.6%)	20 (8.6%)	χ^2^_(1)_ = 0.224, *p =* 0.686; V=0.032^*^
Somatization disorder	6 (4.7%)	7 (6.7%)	13 (5.6%)	χ^2^_(1)_ = 0.410, *p =* 0.575; V=0.042^*^
Other disorders (e.g., eating or stress-related disorders)	16 (12.6%)	5 (4.8%)	21 (9.0%)	χ^2^_(1)_ = 4.288, *p =* 0.041; V=0.136^*^
**CHR criteria** ***n*** **(%)**				χ^2^_(9)_ = **10.957**, ***p** =* **0.279; V** = **0.217**
GRFD only	6 (4.7%)	4 (3.8%)	10 (4.3%)	
GRFD + COGDIS	2 (1.6%)	5 (4.8%)	7 (3.0%)	
GRFD + APS	2 (1.6%)	3 (2.9%)	5 (2.1%)	
GRFD + APS + COGDIS	4 (3.1%)	9 (8.6%)	13 (5.6%)	
COGDIS only	28 (22.0%)	29 (27.6%)	57 (24.6%)	
COGDIS + APS	41 (32.3%)	20 (19.0%)	61 (26.3%)	
COGDIS + BIPS	1 (0.8%)	0 (0.0%)	1 (0.4%)	
APS only	40 (31.5%)	32 (30.5%)	72 (31.0%)	
APS + BIPS	1 (0.8%)	1 (1.0%)	2 (0.9%)	
BIPS only	2 (1.6%)	2 (1.9%)	4 (1.7%)	
**APS** *n* (%)	88 (69.3%)	65 (61.9%)	153 (65.9%)	χ^2^_(1)_ = 1.397, *p =* 0.267; V = 0.078^*^
**BIPS** *n* (%)	4 (3.1%)	3 (2.9%)	7 (3.0%)	χ^2^_(1)_ = 0.017, *p =* 0.897; V = 0.009
**COGDIS** *n* (%)	76 (59.8%)	63 (60.0%)	139 (41.9%)	χ^2^_(1)_ = 0.001, *p =* 1.000; V = 0.002^*^
**GRFD** *n* (%)	14 (11.0%)	21 (20.0%)	35 (15.0%)	χ^2^_(1)_=3.615, *p =* 0.066; V = 0.125^*^
**Schizotypal PD** *n* (%)	4 (3.1%)	11 (10.5%)	15 (6.5%)	χ^2^_(1)_ = 5.102, *p =* 0.024; V = 0.148
**Positive family history of psychosis** *n* (%)	18 (14.2%)	16 (15.2%)	34 (14.7%)	χ^2^_(1)_ = 0.052, *p =* 0.819; V = 0.015
**GF-S** median (mean ± SD)	6 (6.63 ± 1.478)	6 (6.23 ± 1.429)	6 (6.30 ± 1.455)	U = 6,170.0, *p =* 0.315; r = 0.066
**GF-R** median (mean ± SD)	6 (5.83 ± 1.773)	6 (5.71 ± 1.752)	6 (5.78 ± 1.749)	U = 6,431.5, *p =* 0.637; r = −0.030
**SIPS-P1 severity** median (mean ± SD)	3 (2.22 ± 2.093)	0 (1.93 ± 2.053)	3 (2.09 ± 2.076)	U = 6,180.5, *p =* 0.307; r = 0.067
**SIPS-P2 severity** median (mean ± SD)	0 (1.77 ± 2.071)	0 (1.77 ± 2.039)	0 (1.77 ± 2.052)	U = 6,664.5, *p =* 0.995; r = −0.004
**SIPS-P3 severity** median (mean ± SD)	0 (0.13 ± 0.713)	0 (0.37 ± 1.171)	0 (0.24 ± 0.953)	U = 6,244.0, *p =* 0.044; r = −0.132
**SIPS-P4 severity** median (mean ± SD)	3 (2.03 ± 2.055)	0 (1.39 ± 1.968)	0 (1.74 ± 2.037)	U = 5,569.0, *p =* 0.017; r = −0.156
**SIPS-P5 severity** median (mean ± SD)	0 (0.70 ± 1.460)	0 (0.73 ± 1.619)	0 (0.72 ± 1.531)	U = 6,629.5, *p =* 0.913; r = −0.007

^*^Fisher's test, used when any expected cell frequency was < 5. GRFD, Genetic risk and functional deterioration; COGDIS, Cognitive Disturbances; APS, Attenuated psychotic symptoms; BIPS, Brief limited psychotic symptoms; PD, Personality disorder; GF-S, Global Functioning—Social; GF-R, Global Functioning—Role; SIPS, Structured Interview for Psychosis-Risk Syndromes; SIPS-P1, Unusual thought content/delusional ideas; SIPS-P2, Suspiciousness/persecutory ideas; SIPS-P3, Grandiose ideas; SIPS-P4, Perceptual abnormalities/hallucinations; SIPS-5, Disorganized communication; V, Cramer's V with 0.1 = small effect, 0.3 = moderate effect, and 0.5 = large effect; r, Rosenthal's r with 0.1 = small effect, 0.3 = moderate effect, and 0.5 = large effect.

The study was approved by all local ethics committees, and all participants and, where required, the participants' parents/guardians gave written informed consent/assent.

### 2.2. Assessments

CHR criteria and symptoms were assessed using semi-structured clinical interview assessments: UHR criteria were assessed with the Structured Interview for Psychosis-Risk Syndromes (SIPS) (12) and COGDIS with the Schizophrenia Proneness Instrument—Adult version (SPI-A) (46). The five positive SIPS-items (SIPS-P1 “unusual thought content/delusional ideas”; SIPS-P2 “suspiciousness/persecutory ideas”; SIPS-P3 “grandiose ideas”; SIPS-P4 “perceptual abnormalities/hallucinations”; and SIPS-P5 “disorganized communication”) are each rated syndromally for severity based on anchor points ranging from 0 = “absent” to 6 = “severe and psychotic”. These anchors are intended to provide guidelines and examples of signs for every observed symptom but not exact definitions. For example, in P1 “unusual thought content/delusional ideas”, a score of 1 = “questionably present” is rated for symptoms that are perceived as puzzling mind tricks or a sense that something is different, such as déjà-vu experiences, mild changes in perception of time, or vague feelings of estrangement; while a score of 5 = “severe but not psychotic” is appointed to non-paranoid, non-grandiose delusional ideas (such as *Ich-Störungen*, unusual nihilistic, erotomane, religious, referential, or somatic ideas, or unusual ideas about guilt or jealousy) that have become familiar, appear distressingly real, and affect functioning but can still be doubted when contrary evidence or other opinions are presented. A score of 3–5 on a positive SIPS item signifies the presence of an APS, and a score of 6 indicates the presence of a BIPS.

A risk syndrome with APS requires that at least one APS (1) began within the past year or was rated one or more points higher on the severity scale compared to 12 months ago (2), occurred at an average frequency of at least once per week for at least several minutes per event in the past month, and (3) was not better explained by another mental disorder. A risk syndrome with BIPS (that was slightly modified in PRONIA) requires that at least one BIPS (1a) was present at a severity level of 6 within each of the past 3 months (irrespective of the time a severity of 6 was reached) for at least several minutes per day at a frequency of at least once per month or (1b) was present at a severity level of 6 within the past month (irrespective of the time a severity of 6 was reached) for at least several minutes at a mean frequency of at least once per week or in a cumulative frequency of at least 1 h, (2) spontaneously remitted to a severity level of < 6 within 1 week (i.e., without antipsychotic medication), and (3) was not better explained by another mental disorder. Next to these two symptomatic risk syndromes, the SIPS includes the GRFD syndrome that requires (1a) meeting SIPS criteria for a Schizotypal Personality Disorder and/or (1b) having a first-degree relative with a psychotic disorder, and (2) a drop in the global assessment of functioning (GAF) score within the past 12 months by at least 30%.

Contrary to the syndromal item rating of the SIPS, each SPI-A item represents exactly one clearly defined basic symptom, which is rated according to its maximum occurrence within the past 3 months from 0 = “absent” to 6 = “daily”. Basic symptoms are subtle, subjectively experienced disturbances in mental processes including thinking, speech, attention, perception, drive, stress tolerance, and affect that are immediately self-recognized as deviations from “normal” mental processes and commonly not directly observable by others (47). For their spontaneous, immediate self-recognition as disturbances of their own mental processes, basic symptoms are distinct from hallucinatory and delusional APS and BIPS, in which reality testing is disturbed at least to some degree and which are at least briefly perceived as real or realistic. In addition, for the intact immediate self-reflection, basic symptoms are also distinct from observed but often not self-perceived signs of disorganized communication. Because of their strict connection to mental processes, cognitive basic symptoms are therefore not differentiable by thought content like delusional APS and BIPS. The risk syndrome COGDIS requires the presence of at least 2 of the following 9 cognitive basic symptoms with at least weekly occurrence during the last 3 months, i.e., a score ≥3 in the SPI-A: inability to divide attention (B1); captivation of attention by details of the visual field (O7); disturbance of receptive (C4), expressive speech (C5), or abstract thinking (O3); thought interference (C2); blockages (C3) or pressure (D3); and unstable ideas of reference (D4).

To rule out lifetime psychosis and to evaluate other mental disorders, patients were assessed with the Structured Clinical Interview for the Diagnostic and Statistical Manual of mental disorders, fourth edition, Text Revision (DSM-IV-TR) (48). Moreover, the Global Functioning: Social (GF:S) (49) and the Global Functioning: Role (GF:R) (50) assessed social and role functioning. A score of 6 or lower in GF:R and GF:S signify the presence of a functional deficit.

In addition to training of all interviewers, weekly supervision was implemented within each center and monthly CHR case conferences on inclusion-relevant CHR symptoms by phone with the senior author (F.S.-L.), an expert in early detection of psychoses and qualified trainer of both SPI-A and SIPS, were performed to guarantee excellent and reliable data quality.

For supervision purposes, case vignettes of each patient possibly meeting CHR criteria were prepared, giving a synopsis of the patient's description of the content, course, and impact on the functioning of each reported potentially inclusion-relevant CHR symptom, i.e., the five positive SIPS items and the 9 COGDIS symptoms, when scored at least 3. These case vignettes formed the basis for the monthly case conferences, in which they were discussed in detail with F.S.-L. In case the assessment was insufficient, i.e., relevant questions of FS-L could not be answered, the patient was interviewed again for these open questions and the case was discussed again. From these case vignettes, i.e., symptom descriptions, the first author (CT) extracted phenomenological contents of all APS and/or BIPS. All extracted contents were discussed under regular supervision by FS-L (Supplementary Table 2 gives examples of symptom descriptions from case vignettes and the content extracted from these).

### 2.3. Data analyses

Using version 28.0.1.1 of the Statistical Package for the Social Sciences, the frequency of the contents was descriptively compared for sex, age, country, comorbid mental disorders, and GF:S and GF:R using χ^2^-test or Fisher's exact test for nominal and Kruskal–Wallis or Mann–Whitney tests for continuous data. Next, contents showing a cell with a significant Standardized Residual (SR>|1,96|) in χ-test^2^ test or trend significance (*p* < 0.10) were examined for their association with the above factors in regression analyses using logistic regression for binary data with *k* = 2, multinominal regression for categorical data with *k* ≥ 2, and ordinal or linear regression for continuous data. Because an external validation set was not available, we used bootstrapping validation of regression models to predict their fit to a hypothetical testing set.

## 3. Results

### 3.1. Sociodemographic characteristics

Table 1 shows the sample characteristics. Most patients were recruited in the four German centers (53.0%), followed by the three Italian centers (17.2%). Although sex was evenly distributed in the total sample [$\chi_{\left(1\right)}^{2}$ = 2.086, *p* = 0.168), the distribution of sex differed between countries, with a dominance of female participants in Germany and England and a dominance of male participants in Switzerland. Not significantly different between sexes, the median age in the total sample was 22 years and the median education years were 13 years. The majority of the sample was single (72.8%) and in full-time occupation (61.6%).

The median values of GF-S and GF-R were 6 each, indicating moderate impairment in both functional domains. Most patients (73.7%) had a mental disorder next to their CHR status. Of these, mood disorders were the most frequent (50.4%), followed by anxiety disorders (31.0%) which were more frequent in female participants. Disorders of other main diagnostic categories were present in < 10% of the sample. Disorders of main diagnostic categories according to DSM-IV-TR with a presence of < 5% (*n* = 11), such as eating disorders, were summarized as “other disorders” in analyses for power reasons (Table 1).

Regarding CHR criteria, the symptomatic UHR criteria were met by 158 (68.1%) patients. The APS syndrome was the most frequent (65.9%), followed by COGDIS (41.9%). The APS syndrome occurred mainly by itself (31.0%) but also frequently in combination with COGDIS (31.9%). The BIPS syndrome was infrequently met (3.0%), in almost half of the instances in combination with the APS syndrome or COGDIS (Table 1).

### 3.2. Number and type of contents of attenuated and brief intermittent psychotic symptoms

We distinguished 109 different thematic contents of APS and BIPS (Tables 2–**4**): 63 delusional contents (40 included in SIPS-P1, 17 included in SIPS-P2, and six included in SIPS-P3), 29 perceptual aberrations (SIPS-P4), and 17 signs of disorganized communication (SIPS-P5). Only 20 contents (18.3%) were present in more than 5% of patients (Tables 2–**4**). Nine of these were rated at “unusual thought content/delusional ideas” (SIPS-P1; Table 2), four at “suspiciousness/persecutory ideas” (SIPS-P2; Table 2), five at “perceptual abnormalities/hallucinations” (SIPS-P4; Table 3), and two at “disorganized communication” (SIPS-P5; Table 4). Paranoid-referential ideas related to being the focus of negative attention (24.6%) and ideas that others intend to harm the patient in a non-physical way (12.5%) were the two most frequent contents (Table 2). The most frequent content of “grandiose ideas” (SIPS-P4), i.e., “grandiose ideas with respect to own (natural) abilities”, was present in only 4.3% of patients (Table 2). Fifty-one contents (46.7%) were present in < 1% of the sample (Tables 2–4).

**Table 2:** Frequency of attenuated and transient delusional ideas (SIPS-P1, SIPS-P2, and SIPS-P3) in CHR patients in descending order (*N* = 232).

**SIPS No**.	**Content of attenuated and transient delusional ideas**	***n* (%)**
P2	Paranoid ideas of reference (negative gazes of passers-by)	**57 (24.6%)**
P2	Ideas that others intend to harm the patient (not physically)	**29 (12.5%)**
P1E	Ideas of being the center of non-negative attention	**26 (11.2%)**
P1D	Nihilistic ideas about the non-existence of others	**25 (10.8%)**
P2	Ideas that others intend to physically harm the patient	**25 (10.8%)**
P1E	Non-paranoid ideas of being especially addressed by random events (e.g., media)	**24 (10.3%)**
P1B	Experiences of mind being read	**17 (7.3%)**
P1D	Nihilistic ideas about own non-existence	**17 (7.3%)**
P1D	Exaggerated ideas of guilt	**15 (6.5%)**
P1D	Hypochondriac ideas	**14 (6.0%)**
P2	General mistrust	**14 (6.0%)**
P1B	Audible thoughts (heard by others)	**12 (5.2%)**
P1B	Thought insertion	10 (4.3%)
P2	Paranoid ideas of reference involving friends/family	10 (4.3%)
P3	Grandiose ideas with respect to own (natural) abilities	10 (4.3%)
P2	Ideas of persecution	9 (3.9%)
P1C	Belief in supernatural phenomena (ghosts, telepathy, afterlife, power of the universe, etc.)	8 (3.4%)
P2	Mistrust of friends	8 (3.4%)
P2	Increased vigilance due to feeling unsafe	8 (3.4%)
P1B	Thought broadcasting	7 (3.0%)
P2	Ideas of being threatened/observed by supernatural/invisible beings	7 (3.0%)
P1D	Unusual and unrealistic ideas about the own body	6 (2.6%)
P2	Ideas of being excluded	6 (2.6%)
P1B	Experience of being controlled by external forces	5 (2.2%)
P2	Ideas of being under surveillance (not solely observation)	5 (2.2%)
P3	Grandiose ideas of becoming famous	5 (2.2%)
P1D	Ideas of jealousy	4 (1.7%)
P2	Ideas that others would exploit the patient	4 (1.7%)
P3	Grandiose ideas with respect to own supernatural abilities	4 (1.7%)
P1C	Numbers have special meaning (magical thinking)	3 (1.3%)
P1C	Ideas that things in the surrounding have a special meaning (magical thinking)	3 (1.3%)
P1D	Ideas of being part of a movie, computer game, etc.	3 (1.3%)
P3	Grandiose ideas of being chosen to fulfill a greater plan (e.g., by God)	3 (1.3%)
P1B	Thought withdrawal	2 (0.9%)
P1	Ideas that strangers know something about patient	2 (0.9%)
P1C	Ideas of being directly affected by other persons feelings/actions	2 (0.9%)
P1C	Tendency to see relations between random events	2 (0.9%)
P1C	Unusual ideas about the world	2 (0.9%)
P1D	Ideas of the existence of another reality/universe	2 (0.9%)
P1D	Ideas of vanishing from the world	2 (0.9%)
P1D	Nihilistic ideas of being dead/dying	2 (0.9%)
P1D	Ideas of observing oneself from a birds-eye perspective	2 (0.9%)
P1D	Erotomane ideas	2 (0.9%)
P2	Ideas that others intend to poison the patient	2 (0.9%)
P2	Ideas of being at risk of falling victim to terror attacks or similar	2 (0.9%)
P1C	Ideas that own thoughts could become real	1 (0.4%)
P1C	Ideas that own actions would influence the surrounding	1 (0.4%)
P1C	Ideas that positive thoughts might cause bad things	1 (0.4%)
P1C	Belief that everything is connected	1 (0.4%)
P1C	Belief in conspiracy theories	1 (0.4%)
P1C	Ideas that others take over the patient's self/personality	1 (0.4%)
P1D	Belief in fate	1 (0.4%)
P1C	Unusual religious ideas	1 (0.4%)
P1D	Ideas of being pregnant	1 (0.4%)
P1D	Ideas that a part of the soul is separated	1 (0.4%)
P1D	Ideas of not being a human being	1 (0.4%)
P1D	Identity confusion (patient thinks s/he is someone else)	1 (0.4%)
P1D	Demarcation experiences	1 (0.4%)
P2	Ideas that others wish the patient ill	1 (0.4%)
P2	Ideas of being observed anonymously (e.g., by cameras, internet, etc.)	1 (0.4%)
P2	Ideas that supernatural beings intend to harm the patient	1 (0.4%)
P3	Grandiose ideas of becoming enlightened/a higher being	1 (0.4%)
P3	Grandiose ideas of being a god/higher being	1 (0.4%)

P1 “unusual thought content/delusional ideas” with B: section “first rank symptoms”, C: section “overvalued beliefs”, D: section “other unusual thoughts/delusional ideas”, and E: section “non-persecutory ideas of reference”. P2 “suspiciousness/persecutory ideas”. P3 “grandiose ideas”. The bold values indicated the items with a frequency >5%.

**Table 3:** Frequency of attenuated and transient hallucinations (SIPS-P4) in CHR patients in descending order (*N* = 232).

**SIPS No**.	**Content of attenuated and transient hallucinations**	***n* (%)**
P4D	Non-painful bodily sensation	**21 (9.1%)**
P4C	Seeing moving shadows in the corner of the eye	**20 (8.6%)**
P4B	Hearing sounds made by non-living objects	**18 (7.8%)**
P4B	Hearing one's own name being called	**14 (6.0%)**
P4C	Sensing a presence	**14 (6.0%)**
P4B	Hearing of unintelligible voices (e.g., murmur)	**13 (5.6%)**
P4C	Distinct visual hallucinations	11 (4.7%)
P4B	Hearing of insulting voices	9 (3.9%)
P4B	Hearing sounds made by living beings (humans, animals)	8 (3.4%)
P4C	Seeing a person's shape	8 (3.4%)
P4D	Painful bodily sensation	8 (3.4%)
P4B	Hearing of imperative voices	7 (3.0%)
P4B	Hearing of commenting voices	6 (2.6%)
P4D	Sense of changed body functions	6 (2.6%)
P4C	Visual illusions	5 (2.2%)
P4C	Indistinct visual hallucinations	5 (2.2%)
P4D	Sense of being touched	5 (2.2%)
P4E	Olfactory hallucinations	5 (2.2%)
P4B	Acoustic illusions	3 (1.3%)
P4B	Hearing of dialoguing voices	2 (0.9%)
P4D	Dysmorphophobic illusions	2 (0.9%)
P4B	Audible thoughts (not by others)	1 (0.4%)
P4B	Hearing of God's voice	1 (0.4%)
P4C	Sensing the presence of deceased persons	1 (0.4%)
P4C	Illusions of objects moving	1 (0.4%)
P4C	Confusion of persons	1 (0.4%)
P4D	Sense of being infested by parasites	1 (0.4%)
P4D	Sensing normally non-sensible body functions (e.g., blood flow)	1 (0.4%)
P4E	Gustatory hallucinations	1 (0.4%)

P4 “perceptual abnormalities/hallucinations”; with B: section “auditory distortions, illusions, hallucinations”, C: section “visual distortions, illusions, hallucinations”, D: section “somatic distortions, illusions, hallucinations”, and E: section “olfactory and gustatory distortions, illusions, hallucinations'. The bold values indicated the items with a frequency >5%.

**Table 4:** Frequency of speech-disorganized symptoms (SIPS-P5) in CHR patients in descending order (*N* = 232).

**SIPS No**.	**Type of speech-disorganized symptoms**	***n* (%)**
P5	Losing the thread of thoughts (observed by others)	**18 (7.8%)**
P5	Losing the thread of thoughts (self-experienced)	**15 (6.5%)**
P5	Derailment (observed by others)	7 (3.0%)
P5	Tangentiality (observed by others)	7 (3.0%)
P5	Circumstantial speech	5 (2.2%)
P5	Poverty of speech	3 (1.3%)
P5	Communication is vague	2 (0.9%)
P5	Neologisms	2 (0.9%)
P5	Thought blockage by intrusion (observed by others)	2 (0.9%)
P5	Stilted or pedantic speech	2 (0.9%)
P5	Extremely short non-elaborative speech	1 (0.4%)
P5	Derailment (self-experienced)	1 (0.4%)
P5	Paralogia/alogia	1 (0.4%)
P5	Thought blockage by intrusion (self-experienced)	1 (0.4%)
P5	Thought intrusion (observed by others)	1 (0.4%)
P5	Restricted/stereotyped thinking (observed by others)	1 (0.4%)
P5	Use of inadequate words	1 (0.4%)

P5 “disorganized communication”. The bold values indicated the items with a frequency >5%.

### 3.3. Preselection of the contents of attenuated and brief intermittent psychotic symptoms with group differences

In group comparisons, a sex difference was found only for ideas of persecution (Supplementary Tables 3a–c). Thirty-three contents—20 delusional ideas, ten attenuated hallucinations, and three speech-disorganized symptoms—revealed any cell with SR>|1.96| or trend-level significance between countries (Supplementary Tables 4a–c), and five delusional contents and three speech-disorganized symptoms demonstrated any cell with SR>|1,96| or trend-level significance between religions (Supplementary Tables 5a–c). Furthermore, 32 contents, mainly delusional ideas, showed any cell with SR>|1,96| or trend-level significance in the group comparisons of the five main categories of mental disorders (Supplementary Tables 6–10). With respect to age, GF:S and GF:R, 38 contents, mainly delusional ideas, showed trend-level significance (Supplementary Tables 11–13).

### 3.4. Associations of the contents of attenuated and brief intermittent psychotic symptoms with psychosocial and clinical factors

#### 3.4.1. Associations of the contents of attenuated and brief intermittent psychotic symptoms with age and sex

In the significant univariate regression model of sex [Wald(1) = 3.309, *p* = 0.041]; female participants as the reference group) that explained 2.9% of the variance, “ideas of persecution” was associated with male sex [Beta = −1.945; Exp(Beta) = 0.143, 95%CI: −21.283/−0.450).

For age, the regression analysis of the 18 items revealed negative associations with five items (“nihilistic ideas about the non-existence of others”, “demarcation experiences”, “paranoid ideas of reference (gazes of passers-by)”, “indistinct visual hallucinations”, and “sense of being touched”) and positive associations with three items (“identity confusion”, “acoustic illusions”, “extremely short, non-elaborative speech”), whereby 23.6% of the variance was explained (Table 5).

**Table 5:** Association of the contents of attenuated and brief intermittent psychotic symptoms with age, linear regression analysis (*N* = 232).

**SIPS No**.	**Content**	**Unstand. Beta**	**SEa**	**Stand. Beta**	**T**	**pa**	**95% CI; lower^a^**	**95% CI; upper^a^**
P1B	Audible thoughts (heard by others)	−0.783	1.003	−0.033	−0.486	0.436	−3.960	2.394
P1B	Experiences of mind being read	−1.745	0.927	−0.086	−1.297	0.057	−4.397	0.907
P1D	Nihilistic Ideas about the non-existence of others	−1.799	0.714	−0.106	−1.637	0.014	−3.965	0.368
P1D	Identity confusion (patient thinks s/he is s.o. else)	8.812	1.041	0.109	1.785	0.005	−0.917	18.541
P1D	Demarcation experiences	−7.281	0.750	−0.090	−1.472	0.005	−17.028	2.467
P1D	Ideas of jealousy	3.126	5.275	0.077	1.149	0.479	−2.236	8.488
P1E	Ideas of being the center of non-negative attention	−0.441	1.317	−0.026	−0.426	0.768	−2.485	1.602
P2	Paranoid ideas of reference (gazes of passers–by)	−1.870	0.679	−0.152	−2.341	0.014	−3.445	−0.296
P2	Ideas that others would exploit the patient	4.725	5.246	0.116	1.775	0.251	−0.523	9.972
P2	Ideas that others intend to harm the patient (not physically)	1.109	1.075	0.069	1.058	0.336	−0.958	3.175
P2	Ideas of being at risk of falling victim to terror attacks or similar	5.701	3.828	0.100	1.530	0.066	−1.646	13.049
P4B	Acoustic illusions	7.660	3.753	0.164	2.582	0.014	1.813	13.508
P4B	Hearing one's own name being called	−1.817	1.249	−0.082	−1.276	0.109	−4.625	0.990
P4C	Sensing a presence	−1.872	1.122	−0.084	−1.321	0.090	−4.665	0.921
P4	Seeing moving shadows in the corner of the eye	−0.983	0.754	−0.052	−0.787	0.213	−3.444	1.478
P4C	Indistinct visual hallucinations	−3.953	0.594	−0.109	−1.797	0.005	−8.290	0.384
P4D	Sense of being touched	−3.126	0.972	−0.086	−1.334	0.014	−7.744	1.492
P5	Extremely short, non-elaborative speech	14.921	0.438	0.185	3.083	0.005	5.382	24.460

GoF, F_(18)_ = 3.659; *p* < 0.001; R^2^ = 0.236. ^a^Values from Bootstrapping (*N* = 185). P1 “unusual thought content/delusional ideas” with B: section “first rank symptoms”, C: section “overvalued beliefs”, D: section “other unusual thoughts/delusional ideas”, and E: section “non-persecutory ideas of reference.” P2 “suspiciousness/persecutory ideas.” P3 “grandiose ideas.” P4 “perceptual abnormalities/hallucinations”; with B: section “auditory distortions, illusions, hallucinations”, C: section “visual distortions, illusions, hallucinations”, D: section “somatic distortions, illusions, hallucinations”, and E: section “olfactory and gustatory distortions, illusions, hallucinations.” P5 “disorganized communication.” “Ideas that others take over the patient's self/personality” was included in the regression analysis, but due to redundancy was not represented in the results by SPSS.

#### 3.4.2. Associations of the contents of attenuated and brief intermittent psychotic symptoms with country and religion

Although the model of religion became significant and explained 16.4% of the variance, none of the eight contents was a significant predictor (Table 6), also because of the rarity of contents and, relatedly, absence of contents in some subgroups (Supplementary Tables 5a–c) led to 0% confidence levels, indicating that the result is likely unreliable, i.e., unlikely to be repeated in other samples.

**Table 6:** Association of the contents of attenuated and brief intermittent psychotic symptoms with religion, multi-nominal regression analysis (*N* = 232).

**SIPS No**.		**Beta**	**SE^a^**	**Wald (df = 1)**	**p^a^**	**Exp (Beta)**	**95% CI; lower**	**95% CI; upper**
**Christian**								
P1C	Ideas that own actions would influence the surrounding	−0.064	0.000	0.000	n.c.^b^	0.938	0.938	0.938
P1C	Belief in supernatural phenomena (ghosts, telepathy, afterlife, etc.)	−1.846	1.108	2.778	0.096	0.158	0.018	1.384
P1C	Belief in conspiracy theories	−0.064	0.000	0.000	n.c.^b^	0.938	0.938	0.938
P1C	Tendency to see relations between random events	10.055	179.709	0.003	0.955	23,272.36	2.5 E-149	>1 Mio.
P1D	Belief in fate	0.451	0.000	0.000	n.c.^b^	1.570	1.570	1.570
P5	Losing the thread of thoughts (observed by others)	−0.515	0.569	0.822	0.365	0.597	0.196	1.820
P5	Circumstantial speech	0.469	1.240	0.143	0.705	1.599	0.141	18.159
P5	Restricted/stereotyped thinking (observed by others)	−10.589	0.000	0.000	n.c.^b^	2.5 E-5	2.5 E-5	2.5 E-5
**Other**								
P1C	Ideas that own actions would influence the surrounding	21.412	9,963.33	5.0 E-6	0.998	>1 Mio.	0.000	n.c.^b^
P1C	Belief in supernatural phenomena (ghosts, telepathy, afterlife, etc.)	0.996	0.919	1.175	0.278	2.709	0.447	16.418
P1C	Belief in conspiracy theories	21.412	9,963.33	5.0 E-6	0.998	>1 Mio.	0.000	n.c.^b^
P1C	Tendency to see relations between random events	0.523	446.275	1.0 E-6	0.999	1.688	0.000	n.c.^b^
P1D	Belief in fate	20.564	9,963.33	4.0 E-6	0.998	>1 Mio.	0.000	n.c.^b^
P5	Losing the thread of thoughts (observed by others)	0.848	0.781	1.178	0.278	2.334	0.505	10.790
P5	Circumstantial speech	1.883	1.476	1.627	0.202	6.572	0.364	118.578
P5	Restricted/stereotyped thinking (observed by others)	19.006	9,972.02	4.0 E-6	0.998	>1 Mio.	0.000	n.c.^b^

Reference group: Atheists, GoF: $\chi_{\left(16\right)}^{2}$ = 34.300; *p* = 0.005; Nagelkerke's R^2^ = 0.164. ^a^Bootstrapping could not be conducted successfully. ^b^n.c., not calculable due to zero cases with the present item in the reference group. P1 “unusual thought content/delusional ideas” with B: section “first rank symptoms”, C: section “overvalued beliefs”, D: section “other unusual thoughts/delusional ideas”, and E: section “non-persecutory ideas of reference.” P2 “suspiciousness/persecutory ideas.” P3 “grandiose ideas.” P4 “perceptual abnormalities/hallucinations”; with B: section “auditory distortions, illusions, hallucinations”, C: section “visual distortions, illusions, hallucinations”, D: section “somatic distortions, illusions, hallucinations”, and E: section “olfactory and gustatory distortions, illusions, hallucinations.” P5 “disorganized communication.”

The significant regression model of the country with Germany as a reference revealed that 55.8% explained variance (Table 7). Positive associations were found for “hypochondriac ideas” for Finland, and “numbers have a special meaning”, “hypochondriac ideas”, “mistrust against friends”, and “hearing one's own name being called” for Italy (Table 7). No content significantly distinguished England or Switzerland from the reference country Germany.

**Table 7:** Association of the contents of attenuated and brief intermittent psychotic symptoms with country, multi-nominal regression analysis (*N* = 232).

**SIPS No**.		**Beta**	**SE^a^**	**Wald (df = 1)**	**p^a^**	**Exp (Beta)**	**95% CI; lower**	**95% CI; upper**
**England**								
P1B	Experiences of mind being read	−8.729	75.133	0.013	0.908	0.000	1.8 E-68	>1 Mio.
P1C	Ideas that own thoughts could become real	16.631	9,375.49	3.0 E-6	0.999	>1 Mio.	0.000	n.c.^b^
P1C	Ideas that own actions would influence the surrounding	17.337	9,375.57	3.0 E-6	0.999	>1 Mio.	0.000	n.c.^b^
P1C	Numbers have special meaning	−9.213	281.243	0.001	0.974	9.9 E-5	4.0 E-244	>1 Mio.
P1C	Ideas that others take over the patient's self/personality	−0.007	9,374.97	5.8 E-13	1.0	0.993	0.000	n.c.^b^
P1D	Belief in fate	0.166	949.052	3.1 E-8	1.0	1.180	0.000	n.c.^b^
P1C	Ideas that things in the surrounding have a special meaning (no ideas of reference)	−3.922	160.469	0.001	0.980	0.020	5.1 E-139	>1 Mio.
P1D	Hypochondriac ideas	−7.735	79.138	0.010	0.922	0.000	1.9 E-71	>1 Mio.
P1E	Ideas of being pregnant	19.408	4,433.22	1.9 E-5	0.997	>1 Mio.	0.000	n.c.^b^
P1D	Identity confusion (patient thinks s/he is someone else)	−8.890	239.300	0.001	0.970	0.000	2.8 E-208	>1 Mio.
P1D	Ideas of jealousy	−0.850	260.186	1.1 E-5	0.997	0.427	1.5 E-222	>1 Mio.
P2	General mistrust	−7.705	63.615	0.015	0.904	0.000	3.2 E-58	>1 Mio.
P2	Mistrust against friends	−7.682	121.662	0.004	0.950	0.000	1.3 E-107	>1 Mio.
P2	Paranoid ideas of reference involving friends/family	2.945	1.545	3.632	0.057	19.016	0.920	393.206
P2	Ideas that others wish the patient ill	7.675	2,407.59	1.0 E-5	0.997	2,153.29	0.000	n.c.^b^
P2	Ideas that others would exploit the patient	7.873	216.516	0.001	0.971	2,626.56	1.3 E-181	>1 Mio
P2	Ideas of being excluded	−9.435	84.876	0.012	0.911	7.9 E-5	4.5 E-77	>1 Mio
P2	Ideas that others intend to physically harm the patient	0.205	1.154	0.031	0.859	1.227	0.128	11.773
P3	Grandiose ideas of being a god/higher being	16.456	9,375.61	3.0 E-6	0.999	>1 Mio.	0.000	n.c.^b^
P4B	Hearing sounds made by non-living objects	0.032	1.232	0.001	0.979	1.032	0.092	11.541
P4B	Hearing one's own name being called	1.122	1.133	0.980	0.322	3.070	0.333	28.295
P4	Hearing of God's voice	35.819	4,435.55	6.5 E-5	0.994	>1 Mio	0.000	n.c.^b^
P4C	Sensing the presence of deceased persons	8.721	9,375.28	8.6 E-7	0.999	6,132.79	0.000	n.c.^b^
P4C	Illusions of objects moving	0.166	949.052	3.1 E-8	1.0	1.180	0.000	n.c.^b^
P4C	Distinct visual hallucinations	0.927	1.790	0.268	0.605	2.526	0.076	84.395
P4C	Seeing a person's shape	1.831	2.455	0.556	0.456	6.241	0.051	766.724
P4C	Confusion of persons	7.424	987.288	5.7 E-5	0.994	1,676.41	0.000	n.c.^b^
P4D	Painful bodily sensation	−7.891	85.776	0.008	0.927	0.000	3.6 E-77	>1 Mio.
P4	Gustatory hallucinations	−1.665	949.054	3.0 E-6	0.999	0.189	0.000	n.c.^b^
P5	Derailment (self-experienced)	−0.729	2,404.52	9.2 E-8	1.0	0.482	0.000	n.c.^b^
**Switzerland**								
P1B	Experience of mind being read	1.724	0.963	3.208	0.073	5.608	0.850	36.993
P1C	Ideas that own thoughts could become real	16.858	3,429.28	2.4 E-5	0.996	>1 Mio.	0.000	n.c.^b^
P1C	Ideas that own actions would influence the surrounding	17.318	3,429.28	2.6 E-5	0.996	>1 Mio.	0.000	n.c.^b^
P1C	Numbers have special meaning	−8.970	267.515	0.001	0.973	0.000	2.5 E-232	>1 Mio.
P1C	Ideas that others take over the patient's self/personality	18.390	3,429.28	2.9 E-5	0.996	>1 Mio.	0.000	n.c.^b^
P1D	Belief in fate	9.136	910.101	1.0 E-4	0.992	9,283.03	0.000	n.c.^b^
P1C	Ideas that things in the surrounding have a special meaning (no Ideas of reference)	0.496	154.733	1.0 E-5	0.997	1.642	3.2 E-132	>1 Mio.
P1D	Hypochondriac ideas	−7.742	77.007	0.010	0.920	0.000	1.2 E-69	>1 Mio.
P1E	Ideas of being pregnant	0.440	0.000	n.c.^b^	n.c.^b^	1.553	1.553	1.553
P1D	Ideas of not being a human being	−8.647	227.620	0.001	0.970	0.000	3.1 E-198	>1 Mio.
P1D	Ideas of jealousy	−0.231	322.109	5.2 E-7	0.999	0.793	5.3 E-275	>1 Mio.
P2	General mistrust	−0.093	1.475	0.004	0.949	0.911	0.051	16.417
P2	Mistrust against friends	3.492	1.820	3.682	0.055	32.860	0.928	1,163.39
P2	Paranoid ideas of reference involving friends/family	−6.268	57.825	0.012	0.914	0.002	1.1 E-52	>1 Mio.
P2	Ideas that others wish the patient ill	−3.150	2,303.20	2.0 E-6	0.999	0.043	0.000	n.c.^b^
P2	Ideas that others would exploit the patient	0.583	221.665	7.0 E-6	0.998	1.792	3.7 E-189	>1 Mio.
P2	Ideas of being excluded	1.264	1.264	0.999	0.318	3.539	0.297	42.174
P2	Ideas that others intend to physically harm the patient	0.098	1.184	0.007	0.934	1.103	0.108	11.233
P3	Grandiose ideas of being a god/higher being	24.408	3„430.14	5.1 E-5	0.994	>1 Mio.	0.000	n.c.^b^
P4B	Hearing sounds made by non-living objects	−8.696	43.472	0.040	0.841	0.000	1.7 E-41	>1 Mio.
P4B	Hearing one's own name being called	0.200	1.302	0.024	0.878	1.222	0.095	15.664
P4	Hearing of God's voice	−4.776	0.000	n.c.^b^	n.c.^b^	0.008	0.008	0.008
P4C	Sensing the presence of deceased persons	16.666	3,429.28	2.4 E-5	0.996	>1 Mio.	0.000	n.c.^b^
P4C	Illusions of objects moving	9.136	910.101	1.0 E-4	0.992	9,283.03	0.000	n.c.^b^
P4C	Distinct visual hallucinations	−7.292	81.549	0.008	0.929	0.001	2.6 E-73	>1 Mio.
P4C	Seeing a person's shape	−8.887	132.485	0.004	0.947	0.000	2.3 E-117	>1 Mio.
P4C	Confusion of persons	16.811	968.490	3.0 E-4	0.986	>1 Mio.	0.000	n.c.^b^
P4D	Painful bodily sensation	1.447	1.245	1.351	0.245	4.251	0.370	48.804
P4	Gustatory hallucinations	18.023	919.694	3.8 E-4	0.984	>1 Mio.	0.000	n.c.^b^
P5	Derailment (self-experienced)	7.732	2,304.65	1.1 E-5	0.997	2,280.62	0.000	n.c.^b^
**Finland**								
P1	Experiences of mind being read	1.055	0.965	1.197	0.274	2.873	0.434	19.029
P1C	Ideas that own thoughts could become real	−1.395	7,851.01	3.2 E-8	1.0	0.248	0.000	n.c.^b^
P1C	Ideas that own actions would influence the surrounding	9.732	7,851.23	2.0 E-6	0.999	16,852.67	0.000	n.c.^b^
P1C	Numbers have special meaning	−8.656	227.646	0.001	0.970	0.000	2.9 E-198	>1 Mio.
P1C	Ideas that others take over the patient's self/personality	−0.516	7,851.01	4.3 E-9	1.0	0.597	0.000	n.c.^b^
P1D	Belief in fate	0.248	794.777	9.8 E-8	1.0	1.282	0.000	n.c.^b^
P1C	Ideas that things in the surrounding have a special meaning (no ideas of reference)	−13.632	109.730	0.015	0.901	1.2 E-6	4.8 E-100	>1 Mio.
P1D	Hypochondriac ideas	2.099	0.865	5.889	0.015	8.155	1.497	44.415
P1E	Ideas of being pregnant	0.755	0.000	n.c.^b^	n.c.^b^	2.128	2.128	2.128
P1D	Ideas of not being a human being	−8.332	193.697	0.002	0.966	0.000	3.2 E-169	>1 Mio.
P1D	Ideas of jealousy	10.834	82.551	0.017	0.896	50,698.65	2.7 E-66	>1 Mio.
P2	General mistrust	1.095	1.130	0.938	0.333	2.988	0.326	27.392
P2	Mistrust against friends	−8.389	107.560	0.006	0.938	0.000	6.3 E-96	>1 Mio.
P2	Paranoid ideas of reference involving friends/family	3.042	1.563	3.787	0.052	20.938	0.979	447.988
P2	Ideas that others wish the patient ill	22.876	886.104	0.001	0.979	>1 Mio.	0.000	n.c.^b^
P2	Ideas that others would exploit the patient	8.908	85.814	0.011	0.917	7,387.53	6.7 E-70	>1 Mio.
P2	Ideas of being excluded	−10.072	58.621	0.030	0.864	4.2 E-5	5.3 E-55	>1 Mio.
P2	Ideas that others intend to physically harm the patient	1.271	0.710	3.201	0.074	3.565	0.886	14.349
P3	Grandiose ideas of being a god/higher being	−3.670	7,851.01	2.2 E-7	1.0	0.025	0.000	n.c.^b^
P4B	Hearing sounds made by non-living objects	0.507	1.173	0.187	0.666	1.660	0.167	16.537
P4B	Hearing one's own name being called	−7.942	45.149	0.031	0.860	0.000	1.3 E-42	>1 Mio.
P4	Hearing of God's voice	8.088	0.000	n.c.^b^	n.c.^b^	3,255.94	3255.94	3,255.94
P4C	Sensing the presence of deceased persons	−1.571	7,851.01	4.0 E-8	1.0	0.208	0.000	n.c.^b^
P4C	Illusions of objects moving	0.248	794.777	9.8 E-8	1.0	1.282	0.000	n.c.^b^
P4C	Distinct visual hallucinations	1.537	1.159	1.759	0.185	4.649	0.480	45.047
P4C	Seeing a person's shape	1.833	1.394	1.728	0.189	6.250	0.407	96.038
P4C	Confusion of persons	−6.013	800.328	5.6 E-5	0.994	0.002	0.000	
P4D	Painful bodily sensation	−8.828	62.741	0.020	0.888	0.000	5.8 E-58	>1 Mio.
P4	Gustatory hallucinations	−1.584	794.778	4.0 E-6	0.998	0.205	0.000	n.c.^b^
P5	Derailment (self-experienced)	14.222	879.552	2.6 E-4	0.987	>1 Mio.	0.000	n.c.^b^
**Italy**								
P1B	Experiences of mind being read	0.103	1.246	0.007	0.934	1.109	0.096	12.761
P1C	Ideas that own thoughts could become real	0.897	6,922.56	1.7 E-8	1.0	2.452	0.000	n.c.^b^
P1C	Ideas that own actions would influence the surrounding	1.176	6,922.56	2.9 E-8	1.0	3.242	0.000	n.c.^b^
P1C	Numbers have special meaning	2.754	1.259	4.785	0.029	15.702	1.332	185.158
P1C	Ideas that others take over the patient's self/personality	0.042	6,922.56	3.7 E-11	1.0	1.043	0.000	n.c.^b^
P1D	Belief in fate	14.461	347.157	0.002	0.967	>1 Mio.	6.0 E-290	>1 Mio.
P1C	Ideas that things in the surrounding have a special meaning (no ideas of reference)	10.249	58.145	0.031	0.860	28,242.65	9.1 E-46	>1 Mio.
P1D	Hypochondriac ideas	1.834	0.863	4.518	0.034	6.257	1.154	33.935
P1E	Ideas of being pregnant	0.937	0.000	n.c.^b^	n.c.^b^	2.553	2.553	2.553
P1D	Ideas of not being a human being	−8.150	164.928	0.002	0.961	0.000	1.2 E-144	>1 Mio.
P1D	Ideas of jealousy	10.011	82.559	0.015	0.903	22,265.55	1.2 E-66	>1 Mio.
P2	General mistrust	−0.063	0.959	0.004	0.948	0.939	0.143	6.152
P2	Mistrust against friends	3.434	1.431	5.757	0.016	30.989	1.875	512.095
P2	Paranoid ideas of reference involving friends/family	2.096	1.504	1.942	0.163	8.135	0.427	155.110
P2	Ideas that others wish the patient ill	−3.392	1,775.52	4.0 E-6	0.998	0.034	0.000	n.c.^b^
P2	Ideas that others would exploit the patient	10.650	85.809	0.015	0.901	42,203.99	3.8 E-69	>1 Mio.
P2	Ideas of being excluded	−0.176	1.461	0.014	0.904	0.839	0.048	14.694
P2	Ideas that others intend to physically harm the patient	0.895	0.731	1.499	0.221	2.448	0.584	10.262
P3	Grandiose ideas of being a god/higher being	−1.895	6,922.56	7.5 E-8	1.0	0.150	0.000	n.c.^b^
P4B	Hearing sounds made by non-living objects	−0.701	0.978	0.513	0.474	0.496	0.073	3.375
P4B	Hearing one's own name being called	1.675	0.796	4.429	0.035	5.340	1.122	25.413
P4	Hearing of God's voice	−2.600	0.000	n.c.^b^	n.c.^b^	0.074	0.074	0.074
P4C	Sensing the presence of deceased persons	−0.061	6,922.56	7.9 E-11	1.0	0.940	0.000	n.c.^b^
P4C	Illusions of objects moving	14.461	347.157	0.002	0.967	>1 Mio.	6.0 E-290	>1 Mio.
P4C	Distinct visual hallucinations	0.845	1.214	0.484	0.486	2.327	0.216	25.105
P4C	Seeing a person's shape	−18.142	89.917	0.041	0.840	1.3 E-8	3.8 E-85	>1 Mio.
P4C	Confusion of persons	0.046	356.841	1.7 E-8	1.0	1.047	1.9 E-304	>1 Mio.
P4D	Painful bodily sensation	1.745	0.904	3.731	0.053	5.728	0.975	33.665
P4	Gustatory hallucinations	32.603	358.611	0.008	0.928	>1 Mio.	8.1 E-292	n.c.^b^
P5	Derailment (self-experienced)	0.093	1,775.52	2.7 E-9	1.0	1.097	0.000	n.c.^b^

Reference group: Germany, GoF: $\chi_{\left(120\right)}^{2}$ = 169.272; *p* = 0.002; Nagelkerke's R^2^ = 0.558. ^a^Bootstrapping could not be conducted successfully. “Use of inadequate words” (P5) was completely redundant with “Derailment (self-experienced)” (P5) and, thus, not included in the analyses. ^b^n.c., not calculable due to zero cases with present item in reference group. P1 “unusual thought content/delusional ideas” with B: section “first rank symptoms”, C: section “overvalued beliefs”, D: section “other unusual thoughts/delusional ideas”, and E: section “non-persecutory ideas of reference.” P2 “suspiciousness/persecutory ideas.” P3 “grandiose ideas.” P4 “perceptual abnormalities/hallucinations”; with B: section “auditory distortions, illusions, hallucinations”, C: section “visual distortions, illusions, hallucinations”, D: section “somatic distortions, illusions, hallucinations”, and E: section “olfactory and gustatory distortions, illusions, hallucinations.” P5 “disorganized communication.” “Use of inadequate words” and “Identity confusion” were included in the regression analysis, but due to redundancy not represented in the results by SPSS.

#### 3.4.3. Associations of the contents of attenuated and brief intermittent psychotic symptoms with mental disorders and functioning

The regression analyses of mental disorders with the absence of a mental disorder as a reference group revealed significant models for all diagnostic categories that, increasing with the number of significant contents, showed explained variances between 13.7% for mood disorders and 38.3% for obsessive-compulsive disorders (OCD) (Tables 8–**12**).

**Table 8:** Association of the contents of attenuated and brief intermittent psychotic symptoms with the presence of a mood disorder, logistic regression analysis (*N* = 232).

**SIPS No**.	**Content**	**Beta**	**SE^a^**	**Wald (df = 1)**	**p^a^**	**Exp (Beta)**	**95% CI; lower**	**95% CI; upper**
P1C	Belief in supernatural phenomena (ghosts, telepathy, afterlife, etc.)	21.222	0.134	0.000	0.001	>1 Mio.	0.000	n.c.^b^
P4B	Hearing of imperative voices	−1.635	9.628	2.540	0.071	0.195	0.023	1.677
P4C	Visual illusions	21.222	0.133	0.000	0.001	>1 Mio.	0.000	n.c.^b^
P5	Derailment (observed by others)	−1.635	0.144	2.217	0.0907	0.195	0.023	1.677

Reference group: no mood disorder, GoF: $\chi_{\left(4\right)}^{2}$ = 25.156; *p* < 0.001; Nagelkerke's R^2^ = 0.137. ^a^Values from Bootstrapping (*N* = 995). ^b^n.c., not calculable due to zero cases with the present item in the reference group. P1 “unusual thought content/delusional ideas” with B: section “first rank symptoms”, C: section “overvalued beliefs”, D: section “other unusual thoughts/delusional ideas”, and E: section “non-persecutory ideas of reference.” P2 “suspiciousness/persecutory ideas.” P3 “grandiose ideas.” P4 “perceptual abnormalities/hallucinations”; with B: section “auditory distortions, illusions, hallucinations”, C: section “visual distortions, illusions, hallucinations”, D: section “somatic distortions, illusions, hallucinations”, and E: section “olfactory and gustatory distortions, illusions, hallucinations.” P5 “disorganized communication.”

Mood disorders revealed two positive associations with “belief in supernatural phenomena” and “visual illusions” (Table 8). Yet, as with several other contents in the disorder models, the rarity of contents and, relatedly, absence of contents in some subgroups led to 0% confidence levels, indicating that the result is likely unreliable, i.e., unlikely to be repeated in other samples. The model of anxiety disorders that explained 16.4% of variance included one significant but unreliable positive association with “ideas that others intend to poison the patient”, one positive association with “ideas that others intend to harm the patient (not physically)”, one significant unreliable negative association with “grandiose ideas with respect to own abilities”, and one significant negative association with “ideas of being the center of non-negative attention” (Table 9). The model on other disorders included three significant positive associations (with “experience of being controlled by external forces”, “paranoid ideas of reference involving friends/family”, and “painful bodily sensation”) and one unreliable negative association with “unusual religious ideas”, and explained 17.9% of the variance (Table 10). For somatization disorders (Table 11), significant positive associations were found with “thought withdrawal”, “sense of changed body functions”, “neologisms”, and “poverty of speech”, and two unreliable positive associations with “ideas of being pregnant” and “ideas that supernatural beings intend to harm the patient”. The somatization disorder model explained 32.4% of the variance. The model on OCD revealed the second highest rate of explained variance in APS/BIPS contents (38.3%; Table 12). Significant positive associations occurred with “thought withdrawal”, “tendency to see relations between random events”, “nihilistic ideas of being dead/dying”, “ideas of observing oneself from a birds-eye perspective”, and “losing the thread of thoughts (self experienced)”. Additionally, “belief that everything is connected” and “ideas of being observed anonymously” showed positive but unreliable associations with OCD (Table 12).

**Table 9:** Association of the contents of attenuated and brief intermittent psychotic symptoms with presence of an anxiety disorder, logistic regression analysis (*N* = 232).

**SIPS No**.	**Content**	**Beta**	**SE^a^**	**Wald (df = 1)**	**p^a^**	**Exp (Beta)**	**95% CI; lower**	**95% CI; upper**
P3	Grandiose ideas with respect to own (natural) abilities	−20.063	0.392	0.000	0.001	0.000	0.000	n.c.^b^
P4B	Hearing of unintelligible voices (e.g., murmur)	0.976	2.106	2.514	0.078	2.654	0.794	8.866
P4B	Experiences of mind being read	0.732	0.621	1.822	0.182	2.080	0.718	6.026
P1E	Ideas of being the center of non-negative attention	−1.561	6.318	4.147	0.042	0.210	0.047	0.943
P2	Ideas that others intend to poison the patient	22.485	5.458	0.000	0.033	>1 Mio.	0.000	n.c.^b^
P2	Ideas that others intend to harm the patient (not physically)	0.868	0.472	3.827	0.041	2.381	0.998	5.681

Reference group: no anxiety disorder, GoF: $\chi_{\left(6\right)}^{2}$ = 28.706; *p* = < 0.001; Nagelkerke's R^2^ = 0.164. ^a^Values from Bootstrapping (*N* = 871). ^b^n.c., not calculable due to zero cases with the present item in the reference group. P1 “unusual thought content/delusional ideas” with B: section “first rank symptoms”, C: section “overvalued beliefs”, D: section “other unusual thoughts/delusional ideas”, and E: section “non-persecutory ideas of reference.” P2 “suspiciousness/persecutory ideas.” P3 “grandiose ideas.” P4 “perceptual abnormalities/hallucinations”; with B: section “auditory distortions, illusions, hallucinations”, C: section “visual distortions, illusions, hallucinations”, D: section “somatic distortions, illusions, hallucinations”, and E: section “olfactory and gustatory distortions, illusions, hallucinations.” P5 “disorganized communication.”

**Table 10:** Association of the contents of attenuated and brief intermittent psychotic symptoms with other disorders, logistic regression analysis (*N* = 232).

**SIPS No**.	**Content**	**Beta**	**SE^a^**	**Wald (df = 1)**	**p^a^**	**Exp (Beta)**	**95% CI; lower**	**95% CI; upper**
P1B	Experience of being controlled by external forces	2.803	8.383	7.203	0.002	16.494	2.130	127.737
P1C	Unusual religious ideas	−17.634	1.270	1.9 E-7	0.003	0.000	0.000	n.c.^b^
P2	Paranoid ideas of reference involving friends/family	1.762	6.816	3.331	0.025^c^	5.826	0.878	38.662
P4D	Painful bodily sensation	2.470	6.961	7.249	0.003	11.824	1.958	71.400

Reference group: no other disorder, GoF: $\chi_{\left(4\right)}^{2}$ = 13.537; *p* = 0.009; Nagelkerke's R^2^ = 0.179. ^a^Values from Bootstrapping (*N* = 627). ^b^n.c., not calculable due to zero cases with the present item in the reference group. ^c^Because bootstrapping does not require distributional assumptions, the bootstrap provides more accurate inferences when the data are not well-behaved, e.g., including little events, or when the sample size is small (51). Thus, we followed the bootstrapping results, and considered variables significant predictors if the bootstrapping became significant, even if these were non-significant in the initial regression analysis and, therefore, included the 1 within the 95%-CI. P1 “unusual thought content/delusional ideas” with B: section “first rank symptoms”, C: section “overvalued beliefs”, D: section “other unusual thoughts/delusional ideas”, and E: section “non-persecutory ideas of reference.” P2 “suspiciousness/persecutory ideas.” P3 “grandiose ideas.” P4 “perceptual abnormalities/hallucinations”; with B: section “auditory distortions, illusions, hallucinations”, C: section “visual distortions, illusions, hallucinations”, D: section “somatic distortions, illusions, hallucinations”, and E: section “olfactory and gustatory distortions, illusions, hallucinations.” P5 “disorganized communication”.

**Table 11:** Association of the contents of attenuated and brief intermittent psychotic symptoms with somatization disorder, logistic regression analysis (*N* = 232).

**SIPS No**.	**Content**	**Beta**	**SE^a^**	**Wald (df = 1)**	***p* ^a^**	**Exp (Beta)**	**95% CI; lower**	**95% CI; upper**
P1B	Thought withdrawal'	3.016	15.380	4.253	0.004	20.414	1.161	358.798
P1E	Ideas of being pregnant	24.219	0.468	3.6 E-7	0.004	>1 Mio.	0.000	n.c.^b^
P1D	Exaggerated ideas of guilt	0.085	11.282	0.004	0.514	1.089	0.082	14.397
P2	Paranoid ideas of reference (gazes of passers-by)	−18.328	12.046	1.2 E-5	0.124	0.000	0	n.c.^b^
P2	Ideas that supernatural beings intend to harm the patient	24.219	0.468	3.6 E-7	0.004	>1 Mio.	0.000	n.c.^b^
P4D	Sense of changed body functions	2.072	13.616	2.703	0.004^c^	7.940	0.672	93.863
P5	Poverty of speech	1.320	20.535	0.329	0.011^c^	3.744	0.041	340.346
P5	Neologisms	1.320	24.827	0.329	0.014^c^	3.744	0.041	340.346

Reference group: no somatization disorder, GoF: $\chi_{\left(8\right)}^{2}$ = 27.944; *p* < 0.001; Nagelkerke's R^2^ = 0.324. ^a^Values from Bootstrapping (*N* = 281). ^b^n.c., not calculable due to zero cases with the present item in the reference group. ^c^See Table 10. P1 “unusual thought content/delusional ideas” with B: section “first rank symptoms”, C: section “overvalued beliefs”, D: section “other unusual thoughts/delusional ideas”, and E: section “non-persecutory ideas of reference.” P2 “suspiciousness/persecutory ideas.” P3 “grandiose ideas.” P4 “perceptual abnormalities/hallucinations”; with B: section “auditory distortions, illusions, hallucinations”, C: section “visual distortions, illusions, hallucinations”, D: section “somatic distortions, illusions, hallucinations”, and E: section “olfactory and gustatory distortions, illusions, hallucinations.” P5 “disorganized communication.”

**Table 12:** Association of the contents of attenuated and brief intermittent psychotic symptoms with obsessive-compulsive disorder (OCD), logistic regression analysis (*N* = 232).

**SIPS No**.	**Content**	**Beta**	**SE^a^**	**Wald (df = 1)**	***p* ^a^**	**Exp (Beta)**	**95% CI; lower**	**95% CI; upper**
P1B	Thought withdrawal	3.273	14.442	5.015	0.016	26.400	1.504	463.265
P1C	Belief that everything is connected	24.476	0.426	3.7 E-7	0.016^c^	>1 Mio.	0.000	n.c.^b^
P1C	Belief in conspiracy theories	23.912	14.521	3.5 E-7	0.095	>1 Mio.	0.000	n.c.^b^
P1C	Tendency to see relations between random events	3.273	15.060	5.015	0.016	26.400	1.504	463.265
P1D	Belief in fate	21.190	9.559	2.8 E-7	0.206	>1 Mio.	0.000	n.c.^b^
P1D	Nihilistic ideas of being dead/dying	1.806	39.369	0.627	0.032^c^	6.086	0.070	531.122
P1D	Ideas of observing oneself from a birds-eye perspective	1.806	34.961	0.627	0.016^c^	6.086	0.070	531.122
P2	Ideas of being observed anonymously (e.g., by cameras, internet, etc.)	24.476	0.426	3.7 E-7	0.016^c^	>1 Mio.	0.000	n.c.^b^
P3	Grandiose ideas of becoming famous	0.564	14.549	0.068	0.159	1.758	0.025	121.975
P4C	Visual illusions	0.564	9.154	0.068	0.159	1.758	0.025	121.975
P5	Losing the thread of thoughts (self-experienced)	2.165	11.057	1.864	0.016^c^	8.712	0.390	194.766
P5	Losing the thread of thoughts (observed by others)	0.557	10.548	0.125	0.175	1.746	0.079	38.544

Reference group: no OCD, GoF: $\chi_{\left(12\right)}^{2}$ = 43.252; *p* < 0.001; Nagelkerke's R^2^ = 0.383. ^a^Values from Bootstrapping (*N* = 62). ^b^n.c.=not calculable due to zero cases with the present item in the reference group. ^c^See Table 10. P1 “unusual thought content/delusional ideas” with B: section “first rank symptoms”, C: section “overvalued beliefs”, D: section “other unusual thoughts/delusional ideas”, and E: section “non-persecutory ideas of reference.” P2 “suspiciousness/persecutory ideas.” P3 “grandiose ideas.” P4 “perceptual abnormalities/hallucinations”; with B: section “auditory distortions, illusions, hallucinations”, C: section “visual distortions, illusions, hallucinations”, D: section “somatic distortions, illusions, hallucinations”, and E: section “olfactory and gustatory distortions, illusions, hallucinations.” P5 “disorganized communication.”

The model on social functioning (GF:S), which explained 20.9% of the variance, indicated that better social functioning was associated with “seeing moving shadows in the corner of the eye”, while poorer functioning was associated with “thought insertion” and “general mistrust” (Table 13). With regard to role functioning (GF:R), better functioning was associated with “ideas that others would exploit the patient”, while poorer functioning was associated with “neologisms” (Table 14). The GF:R model explained 15.8% of the variance.

**Table 13:** Association of the contents of attenuated and brief intermittent psychotic symptoms with social functioning, ordinal regression analysis (*N* = 232).

**SIPS No**.	**Content**	**Beta**	**SE^a^**	**Wald (df = 1)**	**p**	**95% CI; lower^a^**	**95% CI; upper^a^**
P1B	Thought insertion	−1.806	0.832	8.629	0.003	−3.464	−0.164
P1C	Ideas that strangers know s.th. about patient	−1.803	0.962	1.521	0.217	−3.384	0.000
P1C	Numbers have special meaning	−1.898	0.825	3.199	0.074	−3.621	0.000
P1C	Ideas of being directly affected by other persons feelings/actions	1.902	0.860	1.919	0.166	0.000	2.902
P1D	Ideas of vanishing from the world	−0.122	1.847	0.009	0.924	−2.731	2.976
P2	General mistrust	−1.247	0.651	5.786	0.016	−2.719	−0.141
P2	Ideas that others intend to poison the patient	−2.260	0.840	3.164	0.075	−2.946	0.000
P4	Seeing moving shadows in the corner of the eye	1.011	0.511	5.145	0.023	0.133	2.152
P4D	Painful cenesthesia	−1.208	1.227	2.939	0.086	−3.480	0.484
P5	Communication is vague	−1.803	0.953	1.521	0.217	−2.807	0.000
P5	Poverty of speech	−1.729	6.080	2.062	0.151	−23.373	0.307
P5	Losing the thread of thoughts (self-experienced)	−0.827	0.975	0.805	0.370	−3.138	0.644
P5	Losing the thread of thoughts (observed by others)	0.107	0.796	0.016	0.899	−1.286	2.015
P5	Tangentiality (observed by others)	−1.464	1.148	3.857	0.049	−3.590	1.152
P5	Circumstantial speech	−0.918	1.160	0.724	0.395	−3.513	0.710
P5	Stilted or pedantic speech	−1.602	1.361	0.944	0.331	−3.883	0.783

GoF: $\chi_{\left(16\right)}^{2}$ = 52.407; *p* < 0.001; Nagelkerke's R^2^ = 0.209. ^a^Values from Bootstrapping (*N* = 972). P1 “unusual thought content/delusional ideas” with B: section “first rank symptoms”, C: section “overvalued beliefs”, D: section “other unusual thoughts/delusional ideas”, and E: section “non-persecutory ideas of reference.” P2 “suspiciousness/persecutory ideas.” P3 “grandiose ideas.” P4 “perceptual abnormalities/hallucinations”; with B: section “auditory distortions, illusions, hallucinations”, C: section “visual distortions, illusions, hallucinations”, D: section “somatic distortions, illusions, hallucinations”, and E: section “olfactory and gustatory distortions, illusions, hallucinations.” P5 “disorganized communication.”

**Table 14:** Association of the contents of attenuated and brief intermittent psychotic symptoms with role functioning, ordinal regression analysis (*N* = 232).

**SIPS No**.	**Content**	**Beta**	**SE^a^**	**Wald (df = 1)**	**p**	**95% CI; lower^a^**	**95% CI; upper^a^**
P1B	Thought insertion	−1.177	1.401	3.311	0.069	−3.766	0.804
P1C	Tendency to see relations between random events	−1.771	3.043	1.643	0.200	−5.131	3.297
P1D	Ideas of being part of a movie, computer game etc.	−1.887	6.363	2.114	0.146	−23.834	0.000
P2	Ideas that others would exploit the patient	2.100	1.244	4.653	0.031	1.774	4.756
P3	Grandiose ideas of being a god/higher being	−20.206	10.751	n.c.^b^	n.c.^b^	−24.429	0.000
P5	Neologisms	−3.288	8.982	5.837	0.016	−25.070	0.000
P5	Tangentiality (observed by others)	−0.841	1.315	1.348	0.246	−2.561	0.574
P5	Circumstantial speech	−1.356	3.477	1.378	0.240	−5.296	3.106
P5	Stilted or pedantic speech	−2.075	8.660	1.462	0.227	−24.008	2.153

GoF: $\chi_{\left(9\right)}^{2}$ = 38.799; *p* < 0.001; Nagelkerke's R^2^ = 0.158. ^a^Values from Bootstrapping (*N* = 856). ^b^n.c., not calculable due to only one case with the present item. P1 “unusual thought content/delusional ideas” with B: section “first rank symptoms”, C: section “overvalued beliefs”, D: section “other unusual thoughts/delusional ideas”, and E: section “non-persecutory ideas of reference.” P2 “suspiciousness/persecutory ideas.” P3 “grandiose ideas.” P4 “perceptual abnormalities/hallucinations”; with B: section “auditory distortions, illusions, hallucinations”, C: section “visual distortions, illusions, hallucinations”, D: section “somatic distortions, illusions, hallucinations”, and E: section “olfactory and gustatory distortions, illusions, hallucinations.” P5 “disorganized communication.”

## 4. Discussion

We detailed the contents of APS/BIPS and investigated their association with sex, age, country, religion, functioning, and comorbidities for the first time in a large European CHR sample to gain a better understanding of the clinical presentation of CHR and, in particular, UHR patients. Supporting notions of high heterogeneity of UHR states and the contents of their constituting symptoms (39), we found 109 discernible contents of mainly low prevalence, i.e., a presence in < 5% of patients. Thirty-one reliable contents (28.5%), including eight of the 20 contents with a prevalence of ≥5%, showed an association with either sex, age, country, functional deficits, and/or comorbid mental disorders, while none were associated with religion.

### 4.1. Prevalence rates of the contents of attenuated and brief intermittent psychotic symptoms

In the whole sample, the prevalence rates of the 109 contents, of which 58% (*n* = 63) were delusional, varied between 24.6% (*n* = 57) for “paranoid ideas of reference” (e.g., people watching the patient are thinking negatively about him/her) and 0.4% (*n* = 1); this latter low prevalence rate was found for 33 delusional, hallucinatory, and speech-disorganized contents. When only patients meeting symptomatic UHR criteria (*n* = 158, 68.1%) were considered (Supplementary Table 14), these numbers rose to 36.1 and 0.6%. When we compared our results to the earlier studies of APS contents that used the CAPS (40, 41), in both our whole sample and our APS/BIPS subsample, even the most frequent content in our sample was less frequent than the most frequent CAPS-rated contents (see Supplementary Table 14 for a comparison of contents and their frequencies between our sample and the samples reported by Marshall et al. (39) and Trask et al. (40). In the undergraduate control subsample and the negative schizotypy subsample of Trask et al. (40), “ideas of being thought about in a bad way” (present in 51.2% of controls and 61.9% of negative schizotypes) and “guardedness toward people” (present in 41.5% of controls and 57.1% of negative schizotypes) were most frequent. In positive schizotypes (40) and patients with APS (39), “perplexed by reality” (77.6% in positive schizotypes and 54.1% in APS patients) was the most frequent, with “guardedness toward people” being equally frequent in positive schizotypes (77.6%) but less frequent in APS patients (42.6%). The second most frequent content in the APS sample (39) was “overvalued ideas” (52.3%), which was also among the four most frequent contents in positive (67.3%) and negative schizotypes (38.1%) and controls (22.0%) (40).

The lower ratings of these contents in our sample are likely related to a stricter definition of delusional APS as distinct ideas. Thus, mere feelings, even strong ones, that something might be different, or a self-experienced confusion of memories of dreams with memories of real events as captured by “perplexed by reality” that did not involve distinct ideas about a changed reality were not rated as APS in our study. A stricter rating of APS in our study may also explain the much higher frequency of “guardedness toward people” in the two studies using CAPS (39, 40) as we only rated distinct ideas but not indistinct feelings, such as a vague sense of unease or hypervigilance without a clear source of danger. Our stricter rating is supported by the suggestion of Trask et al. (40) that these most frequent contents may represent mild psychotic-like experiences of below-APS-threshold severity rather than clinically significant APS. Similarly, contents of “overvalued ideas” of the CAPS were only rated at APS severity in our study when related to contents of magical thinking but not when related to assumptions and beliefs, which determine the patient's actions to a morbid degree but are shared by other members of the same culture (*i.e*., overvalued ideas in their original definition), because (attenuated) delusions are false ideas unique to their possessor and not shared by other members of the same culture, or understandable in the context of other abnormal phenomena, such as (attenuated) hallucinations (52).

However, when only the rates in the 158 PRONIA patients meeting symptomatic UHR criteria were considered and compared to the NAPLS-2 APS patient sample (39), *Ich-Störungen* in terms of “thought reading”, “thought broadcast”, or “audible thoughts”; non-paranoid referential, somatic, and persecutory ideas; ideas of guilt, jealousy, or possessing supernatural abilities; as well as “unusual somatic perceptions of pains and bodily functions” showed similar frequencies in both samples (Supplementary Table 14). Only nihilistic and observation ideas (incl. frequent paranoid-referential ideas) were more frequent in the PRONIA sample, while schizotypy-like features (“odd ideas concerning supernatural beings/forces”, “unusual religious ideas”, “magical thinking/overvalued ideas”, and “suspiciousness/paranoid ideation”), grandiose ideas, and perceptual aberrations were more frequent in the NAPLS-2 sample (39). Of note, no unusual thoughts (SIPS-P1) with violent contents involving others or the patient as the victim were reported in our sample, whereas Marshal et al. (39) found them in 19.1% of their sample. Because ratings of paranoid ideas (SIPS-P2) involving physical harm to the patient were also less frequent compared to the NAPLS-2 sample (39, 53), this difference is not explained by potential differences in scoring for violent contents (SIPS-P1 vs. SIPS-P2).

Reflecting cultural characteristics, the higher prevalence rates in the NAPLS-2 sample might be explained by the lower prevalence of schizotypal personality disorders and lesser severity of positive schizotypy reported for Europe and the two main countries of PRONIA, Germany and Italy, compared to the US (54–56). This would be in line with the fact that higher rates of these contents were also found in the positive schizotypy undergraduate sample (40).

### 4.2. Association of the contents of attenuated and brief intermittent psychotic symptoms with sex and age

For sex, we found only one association of male sex with “ìdeas of persecution” that explained little variance. This finding is mainly in line with earlier studies that reported no sex differences (30–34) and did not replicate findings of more disorganization (17, 26) or grandiose ideas (17) in male and more attenuated hallucinations in female patients (29). However, one study (17) reported that paranoid and also speech-disorganization APS predicted psychosis specifically in male patients. Yet, for its minor role in our study, sex likely played a minor role in the differences between the NAPLS-2 (40) and the positive schizotypy samples (40), and our PRONIA sample that comprised the same sex distribution as the positive schizotypy sample and a 12% higher rate of females compared to the NAPLS-2 sample. However, due to mostly low prevalence rates of grandiose contents (P3) and specifically attenuated hallucinations (P4), statistical power may not have been sufficient to replicate the reported sex differences for specific contents, which have not been investigated in other studies so far. Another reason for the frequently higher prevalence rates in the NAPLS-2 and positive schizotypy samples might be the younger age of these samples that were on average 5 years younger than the PRONIA sample. Higher rates of schizotypal features and APS/BIPS have been repeatedly related to younger age, especially an age below 16 years (36, 37, 57–59). Thus, unsurprisingly for the few adolescents included in PRONIA (*n* = 16; 6.9%), age explained only 23.6% of the variance.

Nevertheless, our results support calls for more consideration of age-related factors in CHR research (60). In line with reports from early-onset and adult-onset psychosis (59), multimodal and visual hallucinations were more frequent in younger patients and abnormal acoustic perceptions were more frequent in older patients. Furthermore, some nihilistic contents but the one-off “identity confusion” were more frequent in younger patients, this corresponding to reports of higher levels of bizarre positive symptoms in early-onset compared to adult-onset psychosis (59). Also, “paranoid referential ideas by passers-by” of all ages were more frequent in younger patients, likely reflecting younger patients' liability toward less elaborated and vaguer delusions that are more linked to real experiences (59) and/or the possibly transdiagnostic status of paranoid ideas in minors (45). Finally, the higher frequency of “extremely short, non-elaborative speech” in older patients is in agreement with speech-disorganized symptoms slightly increasing with age in 16–40-year-old community subjects and might reflect aberrations in neurodevelopmental maturation and trajectories in cognitive development (18, 61).

### 4.3. Association of the contents of attenuated and brief intermittent psychotic symptoms with religion and country

In our sample, APS/BIPS content did not predict religion (Supplementary Table 14), although associations between symptoms and religion were demonstrated in schizophrenia patients (62). Yet, we mainly compared Christians with atheists and did not evaluate religiosity, which may mediate differences in APS/BIPS (63).

Despite the many discussed differences between the two US-American samples (39, 40), in our European sample and using Germany as a reference country, we found only a few associations of APS/BIPS with Finland and Italy, and none with Switzerland and England (Table 5). However, the low number of single contents and, consequently, the high number of cells with zero frequency resulted in several non-interpretable findings. “Hypochondriac ideas” were more frequent in Finland and/or Italy. Additionally, “hearing one's name called” in the absence of other persons, magical thinking in terms of numbers having a special meaning, and “mistrust against friends” in terms of positive schizotypal experiences were also more frequent in Italy which had the lowest expression of positive schizotypy in a world-wide 12-country comparison that, however, did not include Germany, Finland, or Switzerland (58). Thus, while this might point toward Germany possibly being even lower in the expression of schizotypy than Italy, the fact that no difference was found between Germany and England, which had the second-highest expression of schizotypy (58), contradicts this explanation. Thus, APS/BIPS likely differ most clearly between countries of clearly different backgrounds, such as the US and Shanghai (64), but also between countries of presumed more similar cultural backgrounds such as European countries and the US. Yet, they also seem to differ between European countries as signified by the fact that, at 55.8%, the country explained most of the variance of APS/BIPS, although these differences may be more subtle and related to overall patterns of contents rather than to the prevalence rates of specific contents. Overall, the results indicate that local or national peculiarities likely shape the expression of APS/BIPS to different degrees and possibly influence the predictive power of additional second-step prediction algorithms, such as the NAPLS-2 risk calculator (65). Therefore, national peculiarities should be more prominently considered in future CHR studies.

### 4.4. Associations of the contents of attenuated and brief intermittent psychotic symptoms with functioning and comorbidities

The association of APS contents with functioning was so far only studied by Trask et al. (40) in undergraduate students. They used the Global Assessment of Functioning (GAF) that is incorporated in the SIPS and assesses social and occupational functioning but also psychological symptoms. Yet, a comparison of the GAF with the symptom-independent Social and Occupational Functioning Assessment Scale (SOFAS) (66), the source scale of GF:S and GF:R, found GAF and SOFAS total scores to be practically exchangeable (67). Despite this correspondence between scales, the selected predictors of functioning differed greatly between our study and the study by Trask et al. (40). Only the CAPE-item “vague figures or shadows” and our corresponding content “seeing moving shadows in the corner of the eye” were selected in both studies. But while, in concert with “being perplexed by reality”, “ideas of guilt”, and “supernatural beliefs”, it predicted lower functioning in the undergraduates (41) and better social functioning in our patient sample; thereby counterbalancing the significant negative impact of “thought insertion”, “general mistrust”, and “tangential speech”. Poor role functioning was only significantly predicted by “neologisms”; this impact was counterbalanced by “ideas that others would exploit the patient”. The concordant negative role of signs of disorganized communication in functioning is consistent with reports of formal thought disorder severity predicting poor social functioning, unemployment, relapses, and re-hospitalizations in early stages of psychosis (68) and of an association between the disorganized schizotypy domain and functional impairment (69). The potential protective role played by the schizotypal perceptual aberration “seeing shadows in the corner of the eye” is possibly reinforcing notions of benign positive schizotypy in the absence of paranoid ideations, including general mistrust and disorganization (70), thus counterbalancing their effects, in particular, in social interactions. Interestingly, in line with earlier notions of better functioning in paranoid compared to non-paranoid psychiatric patients (71), “ideas that others would exploit the patient” was linked to better role functioning, indicating that different paranoid ideas might differentially impact functioning. In doing so, paranoid ideas, specifically “general mistrust” but possibly also ideas that others intend to harm the patient in some ways, may impact negatively more on social than on role functioning (72, 73).

To our best knowledge, this is the first study on the association between non-psychotic comorbidities and contents of APS/BIPS in CHR. Although depressive and anxiety disorders were commonly most frequent in CHR samples, including ours, in line with previous reports that both disorders were associated with negative and disorganized but not positive symptoms (74), APS/BIPS contents hardly predicted either disorder. Rather, other less frequent disorders, especially OCD and somatization disorders, demonstrated differential associations with various bizarre contents, somatic perception abnormalities, some paranoid ideas, magical thinking, and some cognitive disorganization. Yet, again, low frequencies impaired the interpretation of results in some instances.

Although a phenomenological overlap of “thought insertion” and compulsive thoughts is commonly discussed (75), the rare “thought withdrawal” (0.9%) was related to both OCD and somatization disorders. Furthermore, supporting the assumed link between magical ideation and OCD (76), especially neutralizing behaviors (77), some forms of magical thinking (e.g., “tendency to see relations between random events”) were associated with OCD in our study. Reports on depersonalization and related nihilistic ideas in OCD are conflicting (78, 79). Yet, our results indicate some association between nihilistic content, specifically ideas of being dead or dying, and OCD.

Thus, many of the associations of APS/BIPS contents with mental disorders replicate earlier findings and, consequently, have unlikely evolved by chance due to multiple testing. However, future studies should examine if the links of some APS contents with certain non-psychotic mental disorders limit their psychosis-predictive power and if contents with links to reduce functioning show increased risks for psychosis and long-term functional disability that was frequently observed in CHR samples (80).

### 4.5. Strengths and limitations

This study has the following strengths and limitations. The main strengths are the large sample size and the differential extraction of contents that was not limited by predefined categories. Limitations are the cross-sectional nature of the study that does not allow causal relations to be examined, and the lack of an external validation sample. Furthermore, the fact that 83.1% of the contents were reported by < 5% of the sample led to some hardly interpretable results, and limited statistical power in comparisons and the ability to analyze interaction effects of both contents and sociodemographic and clinical variables. Finally, for our emphasis on single contents and not on clusters or syndromes of contents, we did not analyze if the co-occurrence of contents might be additionally influenced by age, sex, country, and/or comorbid disorders.

## 5. Conclusion

Our study highlights the heterogeneity of a set of APS/BIPS contents in CHR patients and their various links with age, country, functioning, and comorbid mental disorders but also the invariance of another set of APS/BIPS contents that might thus be regarded as a symptomatic core of the symptomatic UHR criteria. The symptomatic core in adults and older adolescents seems mainly characterized by *Ich-Störungen* related to others knowing the patient's thoughts and other bizarre delusional contents, more distinct paranoid ideas of being harmed, shaped visual hallucinations, verbal, gustatoric, and olfactoric hallucinations, as well as derailed, circumstantial, or vague speech. The more heterogeneous contents in this age group seem to involve *Ich-Störungen* related to others controlling the patient's thoughts or actions, and schizotypal features, i.e., ideas of reference, magical thinking suspiciousness and ideas of persecution and observation, and unusual perceptual experience, hypochondriac ideas, as well as tangential, associatively loosened, or impoverished speech. This latter set of contents might moderate the clinical relevance, including the psychosis-predictive value, of APS/BIPS syndromes, thus accounting for the large heterogeneity in outcomes, including transition rates, of UHR samples (8, 11). Thus, as already called for with regard to the effect of age in children and young adolescents (60), future studies should examine if the reported associations of certain contents with age, country of assessment, functioning, and/or comorbid mental disorders moderate their related risk for psychosis and, possibly, for social and role functional disability related to CHR states. Knowledge of the influence of age and country, in particular, might support the development of awareness programs and CHR screeners that either well reflect the developmental and local pattern of CHR symptoms, or focus on a core set of contents that are applicable across a greater range of groups and countries.

## Data availability statement

The original contributions presented in the study are included in the article/Supplementary material, further inquiries can be directed to the corresponding author.

## Ethics statement

The studies involving human participants were reviewed and approved by Heinrich-Heine-Universität Düsseldorf. Written informed consent to participate in this study was provided by the participants' legal guardian/next of kin.

## Author contributions

NK, EM, and FS-L made substantial contributions to the conception or design of the work. NK and EM acquired the funding. CT, NO, and FS-L performed the analyses and interpreted the data for the study. CT and FS-L drafted the manuscript. All authors revisited it critically for important intellectual content, provided approval for publication of the content, and agreed to be accountable for all aspects of the study in ensuring that questions related to the accuracy or integrity of any part of the manuscript are appropriately investigated and resolved.

## The PRONIA consortium

The following members of the **PRONIA Consortium** performed the screening, recruitment, rating, examination, and follow-up of the study participants and were involved in implementing the examination protocols of the study, setting up its information technology infrastructure, and organizing the flow and quality control of the data analyzed in this study between the local study sites and the central study database: Shalaila Haas, Alkomiet Hasan, Claudius Hoff, Ifrah Khanyaree, Aylin Melo, Susanna Muckenhuber-Sternbauer, Yanis Köhler, Ömer Öztürk, Nora Penzel, David Popovic, Adrian Rangnick, Sebastian von Saldern, Rachele Sanfelici, Moritz Spangemacher, Ana Tupac, Maria Fernanda Urquijo-Castro, Johanna Weiske, Antonia Wosgien, Camilla Krämer, and Rachele Sanfilici (Department of Psychiatry and Psychotherapy, Ludwig-Maximilian-University, Munich, Germany); Karsten Blume, Dennis Hedderich, Dominika Julkowski, Nathalie Kaiser, Thorsten Lichtenstein, Ruth Milz, Alexandra Nikolaides, Tanja Pilgram, Mauro Seves, and Martina Wassen (Department of Psychiatry and Psychotherapy, University of Cologne, Cologne, Germany); Christina Andreou, Laura Egloff, Fabienne Harrisberger, Ulrike Heitz, Claudia Lenz, Letizia Leanza, Amatya Mackintosh, Renata Smieskova, Erich Studerus, Anna Walter, and Sonja Widmayer (Department of Psychiatry, Psychiatric University Hospital, University of Basel, Basel, Switzerland); Chris Day, Sian Lowri Griffiths, Mariam Iqbal, Mirabel Pelton, Pavan Mallikarjun, Alexandra Stainton, and Ashleigh Lin (Institute for Mental Health and School of Psychology, University of Birmingham, Birmingham, United Kingdom); Alexander Denissoff, Anu Ellilä, Tiina From, Markus Heinimaa, Tuula Ilonen, Päivi Jalo, Heikki Laurikainen, Antti Luutonen, Akseli Mäkela, Janina Paju, Henri Pesonen, Reetta-Liina Säilä, Anna Toivonen, and Otto Turtonen (Department of Psychiatry, University of Turku, Turku, Finland); Sonja Botterweck, Norman Kluthausen, Gerald Antoch, Julian Caspers, Hans-Jörg Wittsack, and Christian Schmidt-Kraepelin (Department of Psychiatry and Psychotherapy, Medical Faculty, Heinrich-Heine University Düsseldorf, Düsseldorf, Germany); Giuseppe Blasi, Giulio Pergola, Grazia Caforio, Leonardo Fazio, Tiziana Quarto, Barbara Gelao, Raffaella Romano, Ileana Andriola, Andrea Falsetti, Marina Barone, Roberta Passiatore, and Marina Sangiuliano (Department of Basic Medical Science, Neuroscience and Sense Organs, University of Bari Aldo Moro, Bari, Italy); Marian Surmann, Olga Bienek, Udo Dannlowski, and Georg Romer (Department of Psychiatry and Psychotherapy, University of Münster, Münster, Germany); Ana Beatriz Solana, Manuela Abraham, and Timo Schirmer (GE Global Research, Inc., Niskayuna, NY, United States); Carlo Altamura, Marika Belleri, Francesca Bottinelli, Adele Ferro, and Marta Re (Department of Neuroscience and Mental Health, Fondazione IRCCS Ca' Granda Ospedale Maggiore Policlinico, Workgroup of Paolo Brambilla, University of Milan, Milan, Italy); Emiliano Monzani and Maurizio Sberna (Programma 2000, Niguarda Hospital, Workgroup of Paolo Brambilla, University of Milan, Milan, Italy); Giampaolo Perna, Maria Nobile, and Alessandra Alciati (San Paolo Hospital, Workgroup of Paolo Brambilla, University of Milan, Milan, Italy); Armando D'Agostino and Lorenzo Del Fabro (Villa San Benedetto Menni, Albese con Cassano, Workgroup of Paolo Brambilla, University of Milan, Milan, Italy); Matteo Balestrieri, Carolina Bonivento, Giuseppe Cabras, and Franco Fabbro (Department of Medical Area, Workgroup of Paolo Brambilla, University of Udine, Udine, Italy); Marco Garzitto and Sara Piccin (IRCCS Scientific Institute E. Medea, Polo FVG, Workgroup of Paolo Brambilla, University of Udine, Udine, Italy); and Dr. Noethen and Dr. Degenhardt (Friedrich-Wilhelms-Universität Bonn, Bonn, Germany).
