# Supplementary material for: The heterogeneity of attenuated and brief limited psychotic symptoms: association of contents with age, sex, country, religion, comorbidities, and functioning
Source: Front Psychiatry. 2023 Jul 7;14:1209485. doi: 10.3389/fpsyt.2023.1209485 (PMC10361815; doi:10.3389/fpsyt.2023.1209485)
Supplement: Supplementary file 1 [file Data_Sheet_1.PDF]

Supplementary Material

The heterogeneity of attenuated psychotic symptoms: association of contents with age, sex, nationality, religion and comorbidities

Christian Theisen*, Marlene Rosen, Eva Meisenzahl, Nikolaos Koutsouleris, Theresa Lichtenstein, Stephan Ruhrmann, Joseph Kambeitz, Lana Kambeitz-Ilankovic, Anita Riecher-Rössler, Katharine Chisholm,,, Rachel Upthegrove, Linda A. Antonucci, Alessandro Bertolino, Alessandro Pigoni,, Raimo K. R. Salokangas, Christos Pantelis, Stephen J. Wood, Rebekka Lencer, Peter Falkai, Jarmo Hietala, Paolo Brambilla, André Schmidt , Christina Andreou, Stefan Borgwardt, Naweed Osman, Frauke Schultze-Lutter, for the PRONIA Consortium

*** Correspondence:** Christian.theisen@hhu.de

Contents

Supplementary Table 1. S3 Guidelines Schizophrenia of the German Association for Psychiatry, Psychotherapy and Psychosomatics (DGPPN, 2005) for the treatment of first-episode psychosis and schizophrenia (translated by first author). 4

Supplementary Table 2. Examples of statements in the case vignettes and the related extracted content of APS/BIPS. 5

Supplementary Table 3a. Sex comparisons of the frequency of attenuated and transient delusional ideas (SIPS-P1, SIPS-P2, SIPS-P3) in CHR patients (N=232). 7

Supplementary Table 3b. Sex comparisons of the frequency of perceptual abnormalities / hallucinations (SIPS-P4) in CHR patients (N=232). 10

Supplementary Table 3c. Sex comparisons of the frequency of signs of disorganized communication (SIPS-P5) in CHR patients (N=232). 11

Supplementary Table 4a. Country comparisons of the frequency of attenuated and transient delusional ideas (SIPS-P1, SIPS-P2, SIPS-P3) in CHR patients (N=232). 12

Supplementary Table 4b. Country comparisons of the frequency of perceptual abnormalities/hallucinations (SIPS-P4) in CHR patients (N=232). 16

Supplementary Table 4c. Country comparisons of the frequency of speech-disorganized symptoms (SIPS-P5) in CHR patients (N=232). 18

Supplementary Table 5a. Religion comparisons of the frequency of attenuated and transient delusional ideas (SIPS-P1, SIPS-P2, SIPS-P3) in CHR patients (N=232). 19

Supplementary Table 5b. Religion comparisons of the frequency of perceptual abnormalities/hallucinations (SIPS-P4) in CHR patients (N=232). 22

Supplementary Table 5c. Religion comparisons of the frequency of speech-disorganized symptoms (SIPS-P5) in CHR patients (N=232). 24

Supplementary Table 6a. Comparisons of the frequency of attenuated and transient delusional ideas (SIPS-P1, SIPS-P2, SIPS-P3) in CHR patients with and without mood disorder (N=232). 25

Supplementary Table 6b. Comparisons of the frequency of perceptual abnormalities/hallucinations (SIPS-P4) in CHR patients with and without mood disorder (N=232). 28

Supplementary Table 6c. Comparisons of the frequency of disorganized communication (SIPS-P5) in CHR patients with and without mood disorder (N=232). 29

Supplementary Table 7a. Comparisons of the frequency of attenuated and transient delusional ideas (SIPS-P1, SIPS-P2, SIPS-P3) in CHR patients with and without anxiety disorder (N=232). 30

Supplementary Table 7b. Comparisons of the frequency of perceptual abnormalities/hallucinations (SIPS-P4) in CHR patients with and without anxiety disorder (N=232). 33

Supplementary Table 7c. Comparisons of the frequency of signs of disorganized communication (SIPS-P5) in CHR patients with and without anxiety disorder (N=232). 34

Supplementary Table 8a. Comparisons of the frequency of attenuated and transient delusional ideas (SIPS-P1, SIPS-P2, SIPS-P3) in CHR patients with and without other disorders (N=232). 35

Supplementary Table 8b. Comparisons of the frequency of perceptual abnormalities/hallucinations (SIPS-P4) in CHR patients with and without other disorders (N=232). 38

Supplementary Table 8c. Comparisons of the frequency of signs of disorganized communication (SIPS-P5) in CHR patients with and without other disorders (N=232). 39

Supplementary Table 9a. Comparisons of the frequency of attenuated and transient delusional ideas (SIPS-P1, SIPS-P2, SIPS-P3) in CHR patients with and without somatization disorder (N=232). 40

Supplementary Table 9b. Comparisons of the frequency of perceptual abnormalities/hallucinations (SIPS-P4) in CHR patients with and without somatization disorder (N=232). 43

Supplementary Table 9c. Comparisons of the frequency of signs disorganized communication (SIPS-P5) in CHR patients with and without somatization disorder (N=232). 44

Supplementary Table 10a. Comparisons of the frequency of attenuated and transient delusional ideas (SIPS-P1, SIPS-P2, SIPS-P3) in CHR patients with and without OCD (N=232). 45

Supplementary Table 10b. Comparisons of the frequency of perceptual abnormalities/hallucinations (SIPS-P4) in CHR patients with and without OCD (N=232). ... 48

Supplementary Table 10c. Comparisons of the frequency of signs of disorganized communication (SIPS-P5) in CHR patients with and without OCD (N=232). 49

Supplementary Table 11a. Comparisons of age of CHR patients with and without the respective attenuated and transient delusional idea (SIPS-P1, SIPS-P2, SIPS-P3; N=232). ... 50

Supplementary Table 11b. Comparisons of age of CHR patients with and without the respective perceptual abnormalities/hallucinations (SIPS-P4; N=232). 53

Supplementary Table 11c. Comparisons of age of CHR patients with and without the respective sign of disorganized communication (SIPS-P5; N=232). 55

Supplementary Table 12a. Comparisons of social functioning (GF:S) of CHR patients with and without the respective attenuated and transient delusional idea (SIPS-P1, SIPS-P2, SIPS-P3; N=232). 56

Supplementary Table 12b. Comparisons of social functioning (GF:S) of CHR patients with and without the respective perceptual abnormalities/hallucinations (SIPS-P4; N=232). 59

Supplementary Table 12c. Comparisons of social functioning (GF:S) of CHR patients with and without the respective sign of disorganized communication (SIPS-P5; N=232). 61

Supplementary Table 13a. Comparisons of role functioning (GF:R) of CHR patients with and without the respective attenuated and transient delusional idea (SIPS-P1, SIPS-P2, SIPS-P3; N=232). 62

Supplementary Table 13b. Comparisons of role functioning (GF:R) of CHR patients with and without the respective perceptual abnormalities/hallucinations (SIPS-P4; N=232). 65

Supplementary Table 13c. Comparisons of role functioning (GF:R) of CHR patients with and without the respective sign of disorganized communication (SIPS-P5; N=232). 67

Supplementary Table 14. Comparison of the prevalence rates (n (%)) of delusional and hallucinatory contents reported for the APS subsample of the NAPLS-2 study (Marshall et al., 2014; N=444) and an US undergraduate sample divided by psychometric schizotypy (Trask et al., 2020; N=153) assessed with the Content of Attenuated Positive Symptoms (CAPS) codebook (Marshall et al., 2011), and our sample (N=232; of these 158 with APS/BIPS). 68

References 76

Supplementary Table 1. S3 Guidelines Schizophrenia of the German Association for Psychiatry, Psychotherapy and Psychosomatics (1) for the treatment of first-episode psychosis and schizophrenia (translated by first author).

**Agent**	**Recommended start dosage**	**DI[1]**	**Target dosage for first-episode psychosis patients (mg/d)**	**Target dosage for patients with multiple episodes (mg/d)**	**Recommended maximum dosage (mg/d)[2]**
Atypical antipsychotic drugs					
Amisulprid	200	(1)-2	100-300	400-800	1200
Aripiprazole	(10)-15	1	15-(30)	15-30	30
Clozapine[3]	25	2-(4)	100 -250	200-450	900
Olanzapine	5-10	1	5-15	5-20	20
Quetiapine	50	2	300-600	400-750	750
Risperidone	2	1-2	1-4	3-6 (10)	16
Ziprasidone	40	2	40-80	80-160	160
Typical antipsychotic drugs					
Fluphenanzin	0.4-10	2-3	2,4-10	10-20	20-(40)
Flupentixol	2-10	1-3	2-10	10-60	60
Haloperidol	1-10	(1)-2	1-4	3-15	100
Perazine	50-150	1-2	100-300	200-600	1000
Perphenanzin	4-24	1-3	6-36	12-42	56
Pimozide	1-4	2	1-4	2-12	16
Zotepine	25-50	2-(4)	50-150	75-150	450
Zuclopenthixol	2-50	1-3	2-10	25-50	75

[1] DI (dosing interval): Recommended distribution of the total dose mentioned throughout the day: one time point=1, two time points=2 etc., maximum doses may need to be spread over several time points.
[2] Highest approved dose as stated in the technical information. Especially for the newer antipsychotics, higher doses are often used in clinical practice ("off-label use") and positive experiences with them (casuistic) were reported.
[3] Clozapine is not usually used to treat first-episode psychotic disorders**.**

Supplementary Table 2. Examples of statements in the case vignettes and the related extracted content of APS/BIPS.

Description in case vignette	Extracted content
"I'm very jealous, in an irrational way. Often this causes frictions with my boyfriend. Sometimes, I realize that I'm exaggerating but at other times I don't. And this is worsening to a degree that I even attacked my boyfriend a couple of times, because I had been sure that he had been with another woman. Luckily, I can still always believe him after a while that he is true."	'Ideas of jealousy' (P1D)
"The world does not exist. It makes no sense, philosophically speaking, that it exists out of chaos. What I don't understand is why I have to exist when everything else is not real."	Nihilistic ideas about the non-existence of others' (P1D)
"I feel not alive, like a doll without feelings, like not being myself; not being here. I doubt for hours a day that I am really present and need much contact with other people to come back to normal. I know that, ultimately, this cannot be real but once I start feeling this way, I really doubt my existence for some time."	‚Nihilistic ideas about own non-existence' (P1D)
"Since 2 months, I am afraid of ghosts every night, although I know that they don't really exist. Yet, the idea that they might exist despite all I thought I knew and that they want to harm me keeps me awake every night."	'Belief in super-natural phenomena' (P1C) and ‚Ideas of being threatened / observed by super-natural/invisible beings' (P2)
"Since some months and several times in a day, I have the feeling that I am in the center of other people's attention. I feel like being watched. I think people simply notice that I am different. Sometimes I find this awkward but mostly I don't question this impression but am quite sure that I am right about this. Some of these times, I think that these people think negatively about me or might want to harm me."	‚Ideas of being the center of non-negative attention' (P1E); ‚Paranoid ideas of reference' (P2) and ‚Ideas that others intend to physically harm the patient' (P2)
"I think that people observe me and that they are against me or laugh at me or laugh at me or have negative thoughts about me or that they are using me. Later, I realize that this is not true, although sometimes I need someone to convince me of that. However, at certain times of the day, I often avoid some streets on my way home, because I could meet there certain students from school who I fear might laugh at me."	'Paranoid ideas of reference (gazes of passers-by)' (P2)
"People are untrustworthy, they watch me in the street and I am concerned they may harm me by saying or thinking negative things about me, although they don't know me."	‚Paranoid ideas of reference (gazes of passers-by)' and ‚Ideas that others intend to harm the patient (not physically)' (P2)
Patient is concerned that others want to use her or hurt her. She always feels suspicious of others and is almost convinced of this. She can hardly change her mind on this, even if others try to convince her. She has these concerns that have intensified within the past year with regard to acquaintances as well as friends and family members.	'Ideas that others would exploit the patient' and 'Ideas that others intend to physically harm the patient' (P2)
Patient thinks that he may become a famous artist (painter and rapper) one day. He thinks that he is particular gifted and that	‚Grandiose ideas with respect to own (natural)

Description in case vignette	Extracted content
people just have not realized this yet. Every day, he spends some time painting and singing but does not take lessons, and believes that he will influence and help other young people with his music and that he has very important messages to convey with his songs. He expects to sell his music to a broad audience and spends some time thinking about becoming famous. However, when questioned about it he expresses some doubts and says that he might not become famous and that “many talented people do not become famous”. Yet, he has already started to put away worthless possessions of his that might be worth a lot in future when he has become famous.	abilities‘ and ‚Grandiose ideas of becoming famous‘ (P3)
“I was standing at the platform when I heard women whispering; at first, I thought there were people around me but then I realized that no one else was there. They were talking so quietly that I couldn’t understand them”	‚Hearing of unintelligible voices‘ (P4B)
“I felt that someone was in my room, sleeping next to me in my bed. Yet, I was alone.”	‚Sensing a presence‘ (P4C)
“I felt I was touched and ran out of the room in panic because I could not see anyone.”	‚Sense of being touched‘ (P4D)
Patient reports that he would frequently see some movement in the corner of his eyes. The moving figures appear to be as big as a dog or even a human but, when he turns his head, nothing is there.	‚Seeing moving shadows in the corner of the eye‘ (P4C)

Supplementary Table 3a. Sex comparisons of the frequency of attenuated and transient delusional ideas (SIPS-P1, SIPS-P2, SIPS-P3) in CHR patients (N=232).

SIPS No.	Content	females (n=127)	males (n=105)	Statistics
P1B	Thought insertion	6 (4.7%)	4 (3.8%)	$\chi^2(1)=0.117$, p=1.0
P1B	Thought withdrawal	2 (1.6%)	0 (0.0%)	$\chi^2(1)=1.668$, p=.502
P1B	Audible thoughts (by others)	6 (4.7%)	6 (5.7%)	$\chi^2(1)=0.115$, p=.735
P1B	Experiences of mind being read	12 (9.4%)	5 (4.8%)	$\chi^2(1)=1.859$, p=.211
P1B	Thought broadcasting	4 (3.1%)	3 (2.9%)	$\chi^2(1)=0.017$, p=1.0
P1B	Experience of being controlled by external forces	4 (3.1%)	1 (1.0%)	$\chi^2(1)=1.316$, p=.381
P1C	Ideas that strangers know something about patient	1 (0.8%)	1 (1.0%)	$\chi^2(1)=0.018$, p=1.0
P1C	Ideas that own thoughts could become real	1 (0.8%)	0 (0.0%)	$\chi^2(1)=0.830$, p=1.0
P1C	Ideas that own actions would influence the surrounding	1 (0.8%)	0 (0.0%)	$\chi^2(1)=0.830$, p=1.0
P1C	Belief in supernatural phenomena (ghosts, telepathy, afterlife, etc.)	4 (3.1%)	4 (3.8%)	$\chi^2(1)=0.075$, p=1.0
P1C	Numbers have special meaning	3 (2.4%)	0 (0.0%)	$\chi^2(1)=2.513$, p=.254
P1C	Ideas that positive thoughts might cause bad things	1 (0.8%)	0 (0.0%)	$\chi^2(1)=0.0830$, p=1.0
P1C	Ideas of being directly affected by other persons feelings/actions	1 (0.8%)	1 (1.0%)	$\chi^2(1)=0.018$, p=1.0
P1C	Belief that everything is connected	1 (0.0%)	1 (1.0%)	$\chi^2(1)=1.215$, p=.453
P1C	Belief in conspiracy theories	1 (0.0%)	1 (1.0%)	$\chi^2(1)=1.215$, p=.453
P1C	Tendency to see relations between random events	2 (1.6%)	0 (0.0%)	$\chi^2(1)=1.668$, p=.502
P1C	Ideas that others take over the patient's self/personality	0 (0.0%)	1 (1.0%)	$\chi^2(1)=1.215$, p=.453
P1D	Belief in fate	1 (0.8%)	0 (0.0%)	$\chi^2(1)=0.830$, p=1.0
P1C	Unusual religious ideas	1 (0.0%)	1 (1.0%)	$\chi^2(1)=1.215$, p=.453
P1C	Unusual ideas about the world	1 (0.8%)	1 (1.0%)	$\chi^2(1)=0.018$, p=1.0
P1C	Ideas that things in the surrounding have a special meaning	2 (1.6%)	1 (1.0%)	$\chi^2(1)=0.174$, p=1.0
P1D	Unusual and unrealistic ideas about the own body	4 (3.1%)	2 (1.9%)	$\chi^2(1)=0.354$, p=.692
P1D	Hypochondriacal ideas	5 (3.9%)	9 (8.9%)	$\chi^2(1)=2.177$, p=.171
P1D	Ideas of being pregnant	1 (0.8%)	0 (0.0%)	$\chi^2(1)=0.830$, p=1.0
P1D	Nihilistic ideas about own non-existence	8 (6.3%)	9 (8.6%)	$\chi^2(1)=0.437$, p=.615
P1D	Ideas of the existence of another reality / universe	0 (0.0%)	2 (1.9%)	$\chi^2(1)=2.440$, p=.204
P1D	Nihilistic ideas about the non-existence of others	11 (8.7%)	14 (13.3%)	$\chi^2(1)=1.305$, p=.291
P1D	Ideas of vanishing from the world	1 (0.8%)	1 (1.0%)	$\chi^2(1)=0.018$, p=1.0

SIPS No.	Content	females (n=127)	males (n=105)	Statistics
P1D	Ideas of being part of a movie. computer game etc.	1 (0.8%)	2 (1.9%)	$\chi^2(1)=0.562$, p=.591
P1D	Ideas that a part of the soul is separated	1 (0.8%)	0 (0.0%)	$\chi^2(1)=0.830$, p=1.0
P1D	Ideas of not being a human being	0 (0.0%)	1 (1.0%)	$\chi^2(1)=1.215$, p=.453
P1D	Identity confusion (patient thinks s/he is someone else)	0 (0.0%)	1 (1.0%)	$\chi^2(1)=1.215$, p=.453
P1D	Nihilistic ideas of being dead / dying	0 (0.0%)	2 (1.9%)	$\chi^2(1)=2.440$, p=.204
P1D	Demarcation experiences	1 (0.8%)	0 (0.0%)	$\chi^2(1)=0.830$, p=1.0
P1D	Ideas of observing oneself from a birds-eye perspective	0 (0.0%)	2 (1.9%)	$\chi^2(1)=2.440$, p=.204
P1D	Exaggerated ideas of guilt	9 (7.1%)	6 (5.7%)	$\chi^2(1)=0.179$, p=.791
P1D	Ideas of jealousy	1 (0.8%)	3 (2.9%)	$\chi^2(1)=1.453$, p=.331
P1D	Erotomane ideas	0 (0.0%)	2 (1.9%)	$\chi^2(1)=2.440$, p=.204
P1E	Ideas of being the center of non-negative attention	12 (9.4%)	14 (13.3%)	$\chi^2(1)=0.872$, p=.406
P1E	Non-paranoid ideas of being especially addressed by random events (e.g., media)	11 (8.7%)	13 (12.4%)	$\chi^2(1)=0.857$p=.392
P2	General mistrust	7 (5.5%)	7 (6.7%)	$\chi^2(1)=.135$, p=.713
P2	Mistrust of friends	3 (2.4%)	5 (4.8%)	$\chi^2(1)=.994$, p=.473
P2	Paranoid ideas of reference (gazes of passers-by)	27 (21.3%)	30 (28.6%)	$\chi^2(1)=1.658$, p=.198
P2	Paranoid ideas of reference involving friends / family	6 (4.7%)	4 (3.8%)	$\chi^2(1)=.117$, p=.1.0
P2	Ideas that others wish the patient ill	1 (0.8%)	0 (0.0%)	$\chi^2(1)=.830$, p=1.0
P2	Ideas that others would exploit the patient	2 (1.6%)	2 (1.9%)	$\chi^2(1)=.037$, p=1.0
P2	Increased vigilance due to feeling unsafe	4 (3.1%)	4 (3.8%)	$\chi^2(1)=.075$, p=1.0
P2	Ideas of being threatened/observed by supernatural / invisible beings	5 (3.9%)	2 (1.9%)	$\chi^2(1)=.811$, p=.461
P2	Ideas of being observed anonymously (e.g. by cameras. internet etc.)	0 (0.0%)	1 (1.0%)	$\chi^2(1)=1.215$, p=.453
P2	Ideas of being under surveillance (not solely observation)	1 (0.8%)	4 (3.8%)	$\chi^2(1)=2.479$, p=.179
P2	Ideas of persecution	8 (6.3%)	1 (1.0%)	$\chi^2(1)=4.407$, p=.043
P2	Ideas of being excluded	4 (3.1%)	2 (1.9%)	$\chi^2(1)=.354$, p=.692
P2	Ideas that others intend to harm the patient (not physically)	15 (11.8%)	14 (13.3%)	$\chi^2(1)=.122$, p=.727
P2	Ideas that others intend to poison the patient	0 (0.0%)	2 (1.9%)	$\chi^2(1)=2.440$, p=.204
P2	Ideas that others intend to physically harm the patient	12 (9.4%)	13 (12.4%)	$\chi^2(1)=.514$, p=.473
P2	Ideas that supernatural beings intend to harm the patient	0 (0.0%)	1 (0.0%)	$\chi^2(1)=1.215$, p=.453
P2	Ideas of being at risk of falling victim to terror attacks or similar	1 (0.8%)	1 (1.0%)	$\chi^2(1)=.018$, p=1.0

SIPS No.	Content	females (n=127)	males (n=105)	Statistics
P3	Grandiose ideas with respect to own (natural) abilities 5	3 (2.4%)	7 (6.7%)	$\chi^2(1)=2.582$, p=.192
P3	Grandiose ideas of becoming famous	1 (0.8%)	4 (3.8%)	$\chi^2(1)=2.489$, p=.179
P3	Grandiose ideas of being chosen to fulfil a greater plan (e.g. by God)	2 (1.6%)	1 (1.0%)	$\chi^2(1)=.174$, p=1.0
P3	Grandiose ideas of becoming enlightened / a higher being	0 (0.0%)	1 (0.0%)	$\chi^2(1)=1.215$, p=.453
P3	Grandiose ideas with respect to own supernatural abilities	3 (2.4%)	1 (1.0%)	$\chi^2(1)=.674$, p=.629
P3	Grandiose ideas of being a god / higher being	0 (0.0%)	1 (1.0%)	$\chi^2(1)=1.215$, p=.453

Contents highlighted in grey have at least trend significance (p<0.100).
Numbers in **bold** signify cells with a standardized residuum ≥ |1.96|.

Supplementary Table 3b. Sex comparisons of the frequency of perceptual abnormalities / hallucinations (SIPS-P4) in CHR patients (N=232).

SIPS No.	Content	females (n=127)	males (n=105)	Statistics
P4B	Acoustic illusions	2 (1.6%)	1 (1.0%)	$\chi^2(1)$=.174, p=1.0
P4B	Hearing sounds made by non-living objects	8 (6.3%)	10 (9.5%)	$\chi^2(1)$=.835, p=.361
P4B	Hearing sounds made by living beings (humans, animals)	5 (3.9%)	3 (2.9%)	$\chi^2(1)$=.201, p=.732
P4B	Audible thoughts (not by others)	1 (0.8%)	0 (0.0%)	$\chi^2(1)$=.830, p=1.0
P4B	Hearing one's own name being called	8 (6.3%)	6 (5.7%)	$\chi^2(1)$=.035, p=.852
P4B	Hearing of unintelligible voices (e.g., murmur)	10 (7.9%)	3 (2.9%)	$\chi^2(1)$=2.735, p=.156
P4B	Hearing of dialoguing voices	2 (1.6%)	0 (0.0%)	$\chi^2(1)$=1.668, p=.502
P4B	Hearing of commenting voices	2 (1.6%)	4 (3.8%)	$\chi^2(1)$=1.139, p=.414
P4B	Hearing of imperative voices	4 (3.1%)	3 (2.9%)	$\chi^2(1)$=.017, p=1.0
P4B	Hearing of insulting voices	3 (2.4%)	6 (5.7%)	$\chi^2(1)$=1.732, p=.306
P4	Hearing of God's voice	1 (0.8%)	0 (0.0%)	$\chi^2(1)$=.830, p=1.0
P4C	Sensing a presence	10 (7.9%)	4 (3.8%)	$\chi^2(1)$=1.675, p=.196
P4C	Sensing the presence of deceased persons	0 (0.0%)	1 (1.0%)	$\chi^2(1)$=1.215, p=.453
P4	Seeing moving shadows in the corner of the eye	14 (11.0%)	6 (5.7%)	$\chi^2(1)$=2.057, p=.152
P4C	Visual illusions	2 (1.6%)	3 (2.9%)	$\chi^2(1)$=.448, p=.661
P4C	Illusions of objects moving	0 (0.0%)	1 (1.0%)	$\chi^2(1)$=1.215, p=.453
P4C	Dysmorphophobic illusions	0 (0.0%)	2 (1.9%)	$\chi^2(1)$=2.440, p=.204
P4C	Indistinct visual hallucinations	4 (3.1%)	1 (1.0%)	$\chi^2(1)$=1.316, p=.381
P4C	Distinct visual hallucinations	8 (6.3%)	3 (2.9%)	$\chi^2(1)$=1.508 p=.353
P4C	Seeing a person's shape	5 (3.9%)	3 (2.9%)	$\chi^2(1)$=.201, p=.732
P4C	Confusion of persons	0 (0.0%)	1 (1.0%)	$\chi^2(1)$=1.215, p=.453
P4D	Sense of being touched	4 (3.1%)	1 (1.0%)	$\chi^2(1)$=1.316, p=.381
P4D	Sense of changed body functions	2 (1.6%)	4 (3.8%)	$\chi^2(1)$=1.139, p=.414
P4D	Non-painful bodily sensation	11 (8.7%)	10 (9.5%)	$\chi^2(1)$=.052, p=.820
P4D	Painful bodily sensation	6 (4.7%)	2 (1.9%)	$\chi^2(1)$=1.373, p=.298
P4D	Sense of being infested by parasites	0 (0.0%)	1 (1.0%)	$\chi^2(1)$=1.215, p=.453
P4D	Sensing normally non-sensible body functions (e.g., blood flow)	0 (0.0%)	1 (1.0%)	$\chi^2(1)$=1.215, p=.453
P4E	Olfactory hallucinations	2 (1.6%)	3 (2.9%)	$\chi^2(1)$=.448, p=.661
P4	Gustatory hallucinations	0 (0.0%)	1 (1.0%)	$\chi^2(1)$=1.215, p=.453

Contents highlighted in grey have at least trend significance ($p<0.100$).
Numbers in **bold** signify cells with a standardized residuum ≥ |1.96|.

Supplementary Table 3c. Sex comparisons of the frequency of signs of disorganized communication (SIPS-P5) in CHR patients (N=232).

SIPS No.	Content	females (n=127)	males (n=105)	Statistics
P5	Communication is vague	1 (0.8%)	1 (1.0%)	$\chi^2(1)$=.018, p=1.0
P5	Poverty of speech	1 (0.8%)	2 (1.9%)	$\chi^2(1)$=.562, p=.591
P5	Neologisms	1 (0.8%)	1 (1.0%)	$\chi^2(1)$=.018, p=1.0
P5	Extremely short, non-elaborative speech	1 (0.8%)	0 (0.0%)	$\chi^2(1)$=.830, p=1.0
P5	Losing the thread of thoughts (self-experienced)	10 (7.9%)	5 (4.8%)	$\chi^2(1)$=.921, p=.337
P5	Losing the thread of thoughts (observed by others)	12 (9.4%)	6 (5.7%)	$\chi^2(1)$=10.120, p=.290
P5	Derailment (self-experienced)	1 (0.8%)	0 (0.0%)	$\chi^2(1)$=.830, p=1.0
P5	Derailment (observed by others)	5 (3.9%)	2 (1.9%)	$\chi^2(1)$=.811, p=.461
P5	Paralogia / alogia	1 (0.8%)	0 (0.0%)	$\chi^2(1)$=.830, p=1.0
P5	Tangentiality (observed by others)	3 (2.4%)	4 (3.8%)	$\chi^2(1)$=.411, p=.704
P5	Thought blockage by intrusion (self-experienced)	0 (0.0%)	1 (1.0%)	$\chi^2(1)$=1.215, p=.453
P5	Thought blockage by intrusion (observed by others)	1 (0.8%)	1 (1.0%)	$\chi^2(1)$=.018, p=1.0
P5	Thought intrusion (observed by others)	0 (0.0%)	1 (1.0%)	$\chi^2(1)$=1.215, p=.453
P5	Circumstantial speech	3 (2.4%)	2 (1.9%)	$\chi^2(1)$=.057, p=1.0
P5	Restricted / stereotyped thinking (observed by others)	1 (0.8%)	0 (0.0%)	$\chi^2(1)$=.830, p=1.0
P5	Stilted or pedantic speech	0 (0.0%)	2 (1.9%)	$\chi^2(1)$=2.440, p=.204
P5	Use of inadequate words	1 (0.8%)	0 (0.0%)	$\chi^2(1)$=.830, p=1.0

Contents highlighted in grey have at least trend significance (p<0.100).
Numbers in **bold** signify cells with a standardized residuum ≥ |1.96|.

Supplementary Table 4a. Country comparisons of the frequency of attenuated and transient delusional ideas (SIPS-P1, SIPS-P2, SIPS-P3) in CHR patients (N=232).

SIPS No.	Content	Ger-many (n=123)	Eng-land (n=19)	Switzer-land (n=21)	Fin-land (n=29)	Italy (n=40)	Statistics
P1B	Thought insertion	3 (2.4%)	1 (5.3%)	1 (4.8%)	3 (10.3%)	2 (5.0%)	$\chi^2(4)=3.703$, $p=.448$
P1B	Thought withdrawal	1 (0.8%)	0 (0.0%)	0 (0.0%)	1 (3.4%)	0 (0.0%)	$\chi^2(4)=2.969$, $p=.563$
P1B	Audible thoughts (by others)	5 (4.1%)	1 (5.3%)	2 (9.5%)	2 (6.9%)	2 (5.0%)	$\chi^2(4)=1.297$, $p=.862$
P1B	Experiences of mind being read	5 (4.1%)	1 (5.3%)	**5 (23.8%)**	4 (13.8%)	2 (5.0%)	$\chi^2(4)=12.552$, $p=.014$
P1B	Thought broadcasting	5 (4.1%)	1 (5.3%)	0 (0.0%)	1 (3.4%)	0 (0.0%)	$\chi^2(4)=2.705$, $p=.608$
P1B	Experience of being controlled by external forces	2 (1.6%)	0 (0.0%)	0 (0.0%)	2 (6.9%)	1 (2.5%)	$\chi^2(4)=4.159$, $p=.385$
P1C	Ideas that strangers know something about patient	2 (1.6%)	0 (0.0%)	0 (0.0%)	0 (0.0%)	0 (0.0%)	$\chi^2(4)=1.788$, $p=.775$
P1C	Ideas that own thoughts could become real	0 (0.0%)	0 (0.0%)	**1 (4.8%)**	0 (0.0%)	0 (0.0%)	$\chi^2(4)=10.091$, $p=.039$
P1C	Ideas that own actions would influence the surrounding	0 (0.0%)	0 (0.0%)	**1 (4.8%)**	0 (0.0%)	0 (0.0%)	$\chi^2(4)=10.091$, $p=.039$
P1C	Belief in supernatural phenomena (ghosts, telepathy, afterlife, etc.)	5 (4.1%)	0 (0.0%)	0 (0.0%)	0 (0.0%)	3 (7.5%)	$\chi^2(4)=4.577$, $p=.333$
P1C	Numbers have special meaning	1 (0.8%)	0 (0.0%)	0 (0.0%)	0 (0.0%)	**2 (5.0%)**	$\chi^2(4)=5.432$, $p=.246$
P1C	Ideas that positive thoughts might cause bad things 1	1 (0.8%)	0 (0.0%)	0 (0.0%)	0 (0.0%)	0 (0.0%)	$\chi^2(4)=.890$, $p=.926$
P1C	Ideas of being directly affected by other persons feelings/actions 3	0 (0.0%)	0 (0.0%)	1 (4.8%)	0 (0.0%)	1 (2.5%)	$\chi^2(4)=6.480$, $p=.166$
P1C	Belief that everything is connected	1 (0.8%)	0 (0.0%)	0 (0.0%)	0 (0.0%)	0 (0.0%)	$\chi^2(4)=.890$, $p=.926$
P1C	Belief in conspiracy theories	0 (0.0%)	0 (0.0%)	0 (0.0%)	0 (0.0%)	1 (2.5%)	$\chi^2(4)=4.821$, $p=.306$
P1C	Tendency to see relations between random events	1 (0.8%)	0 (0.0%)	0 (0.0%)	0 (0.0%)	1 (2.5%)	$\chi^2(4)=1.859$, $p=.762$
P1C	Ideas that others take over the patient's self/personality	0 (0.0%)	0 (0.0%)	**1 (4.8%)**	0 (0.0%)	0 (0.0%)	$\chi^2(4)=10.091$, $p=.039$
P1D	Belief in fate	0 (0.0%)	0 (0.0%)	0 (0.0%)	0 (0.0%)	**1 (2.5%)**	$\chi^2(4)=4.821$, $p=.306$
P1C	Unusual religious ideas	1 (0.8%)	0 (0.0%)	0 (0.0%)	0 (0.0%)	0 (0.0%)	$\chi^2(4)=.890$, $p=.926$
P1C	Unusual ideas about the world	1 (0.8%)	0 (0.0%)	0 (0.0%)	1 (3.4%)	0 (0.0%)	$\chi^2(4)=2.969$, $p=.563$

SIPS No.	Content	Germany (n=123)	England (n=19)	Switzerland (n=21)	Finland (n=29)	Italy (n=40)	Statistics
P1C	Ideas that things in the surrounding have a special meaning (no ideas of reference)	1 (0.8%)	0 (0.0%)	0 (0.0%)	0 (0.0%)	**2 (5.0%)**	$\chi^2(4)=5.432$, p=.246
P1D	Unusual and unrealistic ideas about the own body	4 (3.3%)	0 (0.0%)	0 (0.0%)	1 (3.4%)	1 (2.5%)	$\chi^2(4)=1.365$, p=.850
P1D	Hypochondriacal ideas	3 (2.4%)	0 (0.0%)	1 (4.8%)	4 (13.8%)	**6 (15.0%)**	$\chi^2(4)=12.883$, p=.012
P1E	Ideas of being pregnant	0 (0.0%)	**1 (5.3%)**	0 (0.0%)	0 (0.0%)	0 (0.0%)	$\chi^2(4)=11.259$, p=.024
P1D	Nihilistic ideas about own non-existence	12 (9.8%)	1 (5.3%)	2 (9.5%)	0 (0.0%)	2 (5.0%)	$\chi^2(4)=3.949$, p=.413
P1D	Ideas of the existence of another reality / universe	2 (1.6%)	0 (0.0%)	0 (0.0%)	0 (0.0%)	0 (0.0%)	$\chi^2(4)=1.788$, p=.775
P1D	Nihilistic ideas about the non-existence of others	18 (14.6%)	1 (5.3%)	3 (14.3%)	2 (6.9%)	1 (2.5%)	$\chi^2(4)=6.077$, p=.193
P1D	Ideas of vanishing from the world	0 (0.0%)	0 (0.0%)	1 (4.8%)	1 (3.4%)	0 (0.0%)	$\chi^2(4)=7.589$, p=.108
P1D	Ideas of being part of a movie, computer game etc.	1 (0.8%)	0 (0.0%)	1 (4.8%)	0 (0.0%)	1 (2.5%)	$\chi^2(4)=30.287$, p=.511
P1D	Ideas that a part of the soul is separated	1 (0.8%)	0 (0.0%)	0 (0.0%)	0 (0.0%)	0 (0.0%)	$\chi^2(4)=0.890$, p=.926
P1D	Ideas of not being a human being	1 (0.8%)	0 (0.0%)	0 (0.0%)	0 (0.0%)	0 (0.0%)	$\chi^2(4)=0.890$, p=.926
P1D	Identity confusion (patient thinks s/he is someone else)	0 (0.0%)	0 (0.0%)	**1 (4.8%)**	0 (0.0%)	0 (0.0%)	$\chi^2(4)=10.091$, p=.039
P1D	Nihilistic ideas of being dead / dying	2 (1.6%)	0 (0.0%)	0 (0.0%)	0 (0.0%)	0 (0.0%)	$\chi^2(4)=1.788$, p=.775
P1D	Demarcation experiences	1 (0.0%)	0 (0.0%)	0 (0.0%)	0 (0.0%)	0 (0.0%)	$\chi^2(4)=0.890$, p=.926
P1D	Ideas of observing oneself from a birds-eye perspective	2 (1.6%)	0 (0.0%)	0 (0.0%)	0 (0.0%)	0 (0.0%)	$\chi^2(4)=1.788$, p=.775
P1D	Exaggerated ideas of guilt	9 (7.3%)	0 (0.0%)	1 (3.4%)	1 (3.4%)	4 (10.0%)	$\chi^2(4)=2.842$, p=.588
P1D	Ideas of jealousy	0 (0.0%)	0 (0.0%)	0 (0.0%)	**2 (6.9%)**	2 (5.0%)	$\chi^2(4)=9.972$, p=.041
P1D	Erotomanic ideas 70 (0.0%)	1 (0.8%)	0 (0.0%)	0 (0.0%)	1 (3.4%)	0 (0.0%)	$\chi^2(4)=2.969$, p=.563
P1E	Ideas of being the center of non-negative attention	16 (13.0%)	0 (0.0%)	1 (4.8%)	2 (6.9%)	7 (17.5%)	$\chi^2(4)=5.809$, p=.214
P1E	Non-paranoid ideas of being especially addressed by random events	14 (11.4%)	1 (5.3%)	2 (9.5%)	2 (6.9%)	5 (12.5%)	$\chi^2(4)=1.259$, p=.868

SIPS No.	Content	Germany (n=123)	England (n=19)	Switzerland (n=21)	Finland (n=29)	Italy (n=40)	Statistics
P2	General mistrust	4 (3.3%)	0 (0.0%)	3 (14.3%)	2 (6.9%)	5 (12.5%)	$\chi^2(4)=8.408$, p=.078
P2	Mistrust of friends	1 (0.8%)	1 (5.3%)	1 (4.8%)	1 (3.4%)	**4 (10.0%)**	$\chi^2(4)=80.020$, p=.091
P2	Paranoid ideas of reference (gazes of passers-by)	24 (19.5%)	3 (15.8%)	6 (28.6%)	9 (31.0%)	15 (37.5%)	$\chi^2(4)=6.932$, p=.140
P2	Paranoid ideas of reference involving friends / family	2 (1.6%)	2 (10.5%)	0 (0.0%)	2 (6.9%)	4 (10.0%)	$\chi^2(4)=8.484$, p=.075
P2	Ideas that others wish the patient ill	0 (0.0%)	0 (0.0%)	0 (0.0%)	**1 (3.4%)**	0 (0.0%)	$\chi^2(4)=7.030$, p=.134
P2	Ideas that others would exploit the patient	0 (0.0%)	0 (0.0%)	0 (0.0%)	1 (3.4%)	**3 (7.5%)**	$\chi^2(4)=11.244$, p=.024
P2	Increased vigilance due to feeling unsafe	4 (3.3%)	0 (0.0%)	0 (0.0%)	2 (6.9%)	2 (5.0%)	$\chi^2(4)=2.768$, p=.597
P2	Ideas of being threatened/observed by supernatural / invisible beings	5 (4.1%)	1 (5.3%)	1 (4.8%)	0 (0.0%)	0 (0.0%)	$\chi^2(4)=3.154$, p=.532
P2	Ideas of being observed anonymously (e.g. by cameras, internet etc.)	1 (0.8%)	0 (0.0%)	0 (0.0%)	0 (0.0%)	0 (0.0%)	$\chi^2(4)=0.890$, p=.926
P2	Ideas of being under surveillance (not solely observation)	3 (2.4%)	0 (0.0%)	0 (0.0%)	0 (0.0%)	2 (5.0%)	$\chi^2(4)=3.102$, p=.541
P2	Ideas of persecution	7 (5.7%)	0 (0.0%)	0 (0.0%)	2 (6.9%)	0 (0.0%)	$\chi^2(4)=5.019$, p=.285
P2	Ideas of being excluded	3 (2.4%)	0 (0.0%)	**2 (9.0%)**	0 (0.0%)	0 (0.0%)	$\chi^2(4)=5.298$, p=.258
P2	Ideas that others intend to harm the patient (not physically)	13 (10.6%)	0 (0.0%)	3 (14.3%)	5 (17.2%)	8 (20.0%)	$\chi^2(4)=5.848$, p=.211
P2	Ideas that others intend to poison the patient	1 (0.8%)	0 (0.0%)	0 (0.0%)	1 (3.4%)	0 (0.0%)	$\chi^2(4)=2.969$, p=.563
P2	Ideas that others intend to physically harm the patient	8 (6.5%)	1 (5.3%)	4 (19.0%)	6 (20.7%)	6 (15.0%)	$\chi^2(4)=80.136$, p=.087
P2	Ideas that supernatural beings intend to harm the patient	1 (0.8%)	0 (0.0%)	0 (0.0%)	0 (0.0%)	0 (0.0%)	$\chi^2(4)=0.890$, p=.926
P2	Ideas of being at risk of falling victim to terror attacks or similar	1 (0.8%)	0 (0.0%)	0 (0.0%)	0 (0.0%)	1 (2.5%)	$\chi^2(4)=1.859$, p=.762
P3	Grandiose ideas with respect to own (natural) abilities 5	5 (4.1%)	0 (0.0%)	1 (4.8%)	2 (6.9%)	2 (5.0%)	$\chi^2(4)=1.401$, p=.844
P3	Grandiose ideas of becoming famous	1 (0.8%)	0 (0.0%)	1 (4.8%)	2 (6.9%)	1 (2.5%)	$\chi^2(4)=5.260$, p=.262

SIPS No.	Content	Germany (n=123)	England (n=19)	Switzerland (n=21)	Finland (n=29)	Italy (n=40)	Statistics
P3	Grandiose ideas of being chosen to fulfil a greater plan (e.g. by God)	1 (0.8%)	1 (5.3%)	1 (4.8%)	0 (0.0%)	0 (0.0%)	$\chi^2(4)=50.452$, p=.244
P3	Grandiose ideas of becoming enlightened / a higher being	1 (0.8%)	0 (0.0%)	0 (0.0%)	0 (0.0%)	0 (0.0%)	$\chi^2(4)=0.890$, p=.926
P3	Grandiose ideas with respect to own supernatural abilities	1 (0.8%)	0 (0.0%)	0 (0.0%)	1 (3.4%)	2 (5.0%)	$\chi^2(4)=4.346$, p=.361
P3	Grandiose ideas of being a god / higher being	0 (0.0%)	0 (0.0%)	**1 (4.8%)**	0 (0.0%)	0 (0.0%)	$\chi^2(4)=10.091$, p=.039

Contents highlighted in grey have at least trend significance (p<0.100).
Numbers in **bold** signify cells with a standardized residuum ≥ |1.96|.

Supplementary Table 4b. Country comparisons of the frequency of perceptual abnormalities/hallucinations (SIPS-P4) in CHR patients (N=232).

SIPS No.	Content	Ger-many (n=123)	Eng-land (n=19)	Switzer-land (n=21)	Fin-land (n=28)	Italy (n=40)	Statistics
P4B	Acoustic illusions	3 (2.4%)	0 (0.0%)	0 (0.0%)	0 (0.0%)	0 (0.0%)	$\chi^2(4)$=2.693, p=.610
P4B	Hearing sounds made by non-living objects	8 (6.5%)	1 (5.3%)	0 (0.0%)	1 (3.4%)	**8 (20.0%)**	$\chi^2(4)$=11.331, p=.023
P4B	Hearing sounds made by living beings (humans, animals)	3 (2.4%)	0 (0.0%)	1 (4.8%)	2 (6.9%)	2 (5.0%)	$\chi^2(4)$=2.489, p=647
P4B	Audible thoughts (not by others)	1 (0.8%)	0 (0.0%)	0 (0.0%)	0 (0.0%)	0 (0.0%)	$\chi^2(4)$=0.890, p=.926
P4B	Hearing one's own name being called	5 (4.1%)	2 (10.5%)	1 (4.8%)	0 (0.0%)	**6 (15.0%)**	$\chi^2(4)$=9.110, p=.058
P4B	Hearing of unintelligible voices (e.g., murmur)	5 (4.1%)	1 (5.3%)	0 (0.0%)	3 (10.3%)	4 (10.0%)	$\chi^2(4)$=4.495, p=.343
P4B	Hearing of dialoguing voices 9	1 (0.8%)	0 (0.0%)	0 (0.0%)	1 (3.4%)	0 (0.0%)	$\chi^2(4)$=2.969, p=.563
P4B	Hearing of commenting voices	5 (4.1%)	0 (0.0%)	0 (0.0%)	0 (0.0%)	1 (2.5%)	$\chi^2(4)$=2.901, p=.575
P4B	Hearing of imperative voices	5 (4.1%)	1 (5.3%)	0 (0.0%)	0 (0.0%)	1 (2.5%)	$\chi^2(4)$=2.381, p=.666
P4B	Hearing of insulting voices	4 (3.3%)	1 (5.3%)	0 (0.0%)	2 (6.9%)	2 (5.0%)	$\chi^2(4)$=1.918, p=.751
P4	Hearing of God's voice	0 (0.0%)	**1 (5.3%)**	0 (0.0%)	0 (0.0%)	0 (0.0%)	$\chi^2(4)$=11.259, p=.024
P4C	Sensing a presence	9 (7.3%)	1 (5.3%)	0 (0.0%)	3 (10.3%)	1 (2.5%)	$\chi^2(4)$=3.557 p=.469
P4C	Sensing the presence of deceased persons	0 (0.0%)	0 (0.0%)	**1 (4.8%)**	0 (0.0%)	0 (0.0%)	$\chi^2(4)$=100.09, p=.039
P4	Seeing moving shadows in the corner of the eye	12 (9.8%)	0 (0.0%)	3 (14.3%)	1 (3.4%)	4 (10.0%)	$\chi^2(4)$=3.931, p=.415
P4C	Visual illusions	4 (3.3%)	0 (0.0%)	0 (0.0%)	0 (0.0%)	1 (2.5%)	$\chi^2(4)$=2.244, p=.691
P4C	Illusions of objects moving	0 (0.0%)	0 (0.0%)	0 (0.0%)	0 (0.0%)	**1 (2.5%)**	$\chi^2(4)$=4.821, p=.306
P4C	Dysmorphophobic illusions	1 (0.8%)	0 (0.0%)	0 (0.0%)	0 (0.0%)	1 (2.5%)	$\chi^2(4)$=1.859, p=.762
P4C	Indistinct visual hallucinations	2 (1.6%)	1 (5.3%)	1 (4.8%)	1 (3.4%)	0 (0.0%)	$\chi^2(4)$=2.821, p=.588
P4C	Distinct visual hallucinations	2 (1.6%)	2 (10.5%)	0 (0.0%)	**5 (17.2%)**	2 (5.0%)	$\chi^2(4)$=15.135, p=.004
P4C	Seeing a person's shape	2 (1.6%)	1 (5.3%)	0 (0.0%)	**4 (13.8%)**	1 (2.5%)	$\chi^2(4)$=11.594, p=.021
P4C	Confusion of persons	0 (0.0%)	0 (0.0%)	0 (0.0%)	0 (0.0%)	**1 (2.5%)**	$\chi^2(4)$=4.821, p=.306
P4D	Sense of being touched	3 (2.4%)	0 (0.0%)	0 (0.0%)	0 (0.0%)	2 (5.0%)	$\chi^2(4)$=3.102, p=.541
P4D	Sense of changed body functions	3 (2.4%)	0 (0.0%)	1 (4.8%)	2 (6.9%)	0 (0.0%)	$\chi^2(4)$=4.110, p=.391

SIPS No.	Content	Ger-many (n=123)	Eng-land (n=19)	Switzer-land (n=21)	Fin-land (n=28)	Italy (n=40)	Statistics
P4D	Non-painful bodily sensation	13 (10.6%)	0 (0.0%)	1 (4.8%)	2 (6.9%)	5 (12.5%)	$\chi^2(4)=3.446$, p=.486
P4D	Painful bodily sensation	3 (2.4%)	0 (0.0%)	1 (4.8%)	0 (0.0%)	**4 (10.0%)**	$\chi^2(4)=7.357$, p=.118
P4D	Sense of being infested by parasites	1 (0.8%)	0 (0.0%)	0 (0.0%)	0 (0.0%)	0 (0.0%)	$\chi^2(4)=0.890$, p=.926
P4D	Sensing normally non-sensible body functions (e.g., blood flow)	1 (0.8%)	0 (0.0%)	0 (0.0%)	0 (0.0%)	0 (0.0%)	$\chi^2(4)=0.890$, p=.926
P4E	Olfactory hallucinations	3 (2.4%)	0 (0.0%)	0 (0.0%)	0 (0.0%)	2 (5.0%)	$\chi^2(4)=3.102$, p=.541
P4E	Gustatory hallucinations	0 (0.0%)	0 (0.0%)	0 (0.0%)	0 (0.0%)	**1 (2.5%)**	$\chi^2(4)=4.821$, p=.306

Contents highlighted in grey have at least trend significance (p<0.100).
Numbers in **bold** signify cells with a standardized residuum ≥ |1.96|.

Supplementary Table 4c. Country comparisons of the frequency of speech-disorganized symptoms (SIPS-P5) in CHR patients (N=232).

SIPS No.	Content	Germany (n=123)	England (n=19)	Switzerland (n=21)	Finland (n=28)	Italy (n=40)	Statistics
P5	Communication is vague	2 (1.6%)	0 (0.0%)	0 (0.0%)	0 (0.0%)	0 (0.0%)	$\chi^2(4)=1.788$, p=.775
P5	Poverty of speech	1 (0.8%)	0 (0.0%)	0 (0.0%)	1 (3.4%)	1 (2.5%)	$\chi^2(4)=2.258$, p=.688
P5	Neologisms	1 (0.8%)	0 (0.0%)	0 (0.0%)	1 (3.4%)	0 (0.0%)	$\chi^2(4)=2.969$, p=.563
P5	Extremely short, non-elaborative speech	1 (0.8%)	0 (0.0%)	0 (0.0%)	0 (0.0%)	0 (0.0%)	$\chi^2(4)=0.890$, p=.926
P5	Losing the thread of thoughts (self-experienced)	9 (7.3%)	0 (0.0%)	0 (0.0%)	1 (3.4%)	5 (12.5%)	$\chi^2(4)=5.758$, p=.218
P5	Losing the thread of thoughts (observed by others)	11 (8.9%)	0 (0.0%)	0 (0.0%)	1 (3.4%)	6 (15.0%)	$\chi^2(4)=7.289$, p=.121
P5	Derailment (self-experienced)	0 (0.0%)	0 (0.0%)	0 (0.0%)	**1 (3.4%)**	0 (0.0%)	$\chi^2(4)=7.030$, p=.134
P5	Derailment (observed by others)	2 (1.6%)	0 (0.0%)	0 (0.0%)	2 (6.9%)	3 (7.5%)	$\chi^2(4)=6.296$, p=.178
P5	Paralogia / alogia	1 (0.8%)	0 (0.0%)	0 (0.0%)	0 (0.0%)	0 (0.0%)	$\chi^2(4)=0.890$, p=.926
P5	Tangentiality (observed by others)	4 (3.3%)	2 (10.5%)	0 (0.0%)	0 (0.0%)	1 (2.5%)	$\chi^2(4)=5.276$, p=260.
P5	Thought blockage by intrusion (self-experienced)	1 (0.8%)	0 (0.0%)	0 (0.0%)	0 (0.0%)	0 (0.0%)	$\chi^2(4)=0.890$, p=.926
P5	Thought blockage by intrusion (observed by others)	2 (1.6%)	0 (0.0%)	0 (0.0%)	0 (0.0%)	0 (0.0%)	$\chi^2(4)=1.788$, p=.775
P5	Thought intrusion (observed by others)	1 (0.8%)	0 (0.0%)	0 (0.0%)	0 (0.0%)	0 (0.0%)	$\chi^2(4)=0.890$, p=.926
P5	Circumstantial speech	5 (4.1%)	0 (0.0%)	0 (0.0%)	0 (0.0%)	0 (0.0%)	$\chi^2(4)=4.528$, p=.339
P5	Restricted / stereotyped thinking (observed by others)	1 (0.8%)	0 (0.0%)	0 (0.0%)	0 (0.0%)	0 (0.0%)	$\chi^2(4)=0.890$, p=.926
P5	Stilted or pedantic speech	2 (1.6%)	0 (0.0%)	0 (0.0%)	0 (0.0%)	0 (0.0%)	$\chi^2(4)=1.788$, p=.775
P5	Use of inadequate words	0 (0.0%)	0 (0.0%)	0 (0.0%)	**1 (3.4%)**	0 (0.0%)	$\chi^2(4)=7.030$, p=.134

Numbers in **bold** signify cells with a standardized residuum ≥ |1.96|.

Supplementary Table 5a. Religion comparisons of the frequency of attenuated and transient delusional ideas (SIPS-P1, SIPS-P2, SIPS-P3) in CHR patients (N=232).

SIPS No.	Content	Atheist (n=93)	Christian (n=121)	Other (n=18)	Statistics
P1B	Thought insertion	5 (5.4%)	5 (4.1%)	0 (0.0%)	$\chi^2(2)$=1.076, p=.584
P1B	Thought withdrawal	2 (2.2%)	0 (0.0%)	0 (0.0%)	$\chi^2(2)$=30.015, p=.221
P1B	Audible thoughts (by others)	8 (8.6%)	4 (3.3%)	0 (0.0%)	$\chi^2(2)$=4.072, p=.131
P1B	Experiences of mind being read	8 (8.6%)	9 (7.4%)	0 (0.0%)	$\chi^2(2)$=1.648, p=.439
P1B	Thought broadcasting	4 (4.3%)	3 (2.5%)	0 (0.0%)	$\chi^2(2)$=1.203, p=.548
P1B	Experience of being controlled by external forces	1 (1.1%)	4 (3.3%)	0 (0.0%)	$\chi^2(2)$=1.670, p=.434
P1C	Ideas that strangers know something about patient	0 (0.0%)	2 (1.7%)	0 (0.0%)	$\chi^2(2)$=1-851, p=.396
P1C	Ideas that own thoughts could become real	0 (0.0%)	1 (0.8%)	0 (0.0%)	$\chi^2(2)$=0.921, p=.631
P1C	Ideas that own actions would influence the surrounding	0 (0.0%)	0 (0.0%)	**1 (5.6%)**	$\chi^2(2)$=11.940, p=.003
P1C	Belief in supernatural phenomena (ghosts, telepathy, afterlife, etc.)	5 (5.4%)	1 (0.8%)	2 (11.1%)	$\chi^2(2)$=6.711, p=.035
P1C	Numbers have special meaning	3 (3.2%)	0 (0.0%)	0 (0.0%)	$\chi^2(2)$=4.543, p=.103
P1C	Ideas that positive thoughts might cause bad things	1 (1.1%)	0 (0.0%)	0 (0.0%)	$\chi^2(2)$=1.501, p=.472
P1C	Ideas of being directly affected by other persons feelings/actions	1 (1.1%)	1 (0.8%)	0 (0.0%)	$\chi^2(2)$=0.208, p=.901
P1C	Belief that everything is connected	1 (1.1%)	0 (0.0%)	0 (0.0%)	$\chi^2(2)$=1.501, p=.472
P1C	Belief in conspiracy theories	0 (0.0%)	0 (0.0%)	**1 (5.6%)**	$\chi^2(2)$=11.940, p=.003
P1C	Tendency to see relations between random events	0 (0.0%)	1 (0.8%)	**1 (5.6%)**	$\chi^2(2)$=5.450, p=.066
P1C	Ideas that others take over the patient's self/personality	1 (1.1%)	0 (0.0%)	0 (0.0%)	$\chi^2(2)$=1.501, p=.472
P1D	Belief in fate	0 (0.0%)	0 (0.0%)	**1 (5.6%)**	$\chi^2(2)$=11.940, p=.003
P1C	Unusual religious ideas	0 (0.0%)	1 (0.8%)	0 (0.0%)	$\chi^2(2)$=0.921, p=.631
P1C	Unusual ideas about the world	1 (1.1%)	1 (0.8%)	0 (0.0%)	$\chi^2(2)$=0.208, p=.901
P1C	Ideas that things in the surrounding have a special meaning (no ideas of reference)	1 (1.1%)	2 (1.7%)	0 (0.0%)	$\chi^2(2)$=0.393, p=.822

SIPS No.	Content	Atheist (n=93)	Christian (n=121)	Other (n=18)	Statistics
P1D	Unusual and unrealistic ideas about the own body	2 (2.2%)	4 (3.3%)	0 (0.0%)	$\chi^2(2)=0.797$, p=.671
P1D	Hypochondriacal ideas	5 (5.4%)	9 (7.4%)	0 (0.0%)	$\chi^2(2)=1.647$, p=.439
P1D	Ideas of being pregnant	1 (1.1%)	0 (0.0%)	0 (0.0%)	$\chi^2(2)=1.501$, p=.472
P1D	Nihilistic ideas about own non-existence	10 (10.8%)	6 (5.0%)	1 (5.6%)	$\chi^2(2)=2.690$, p=.261
P1D	Ideas of the existence of another reality / universe	0 (0.0%)	2 (1.7%)	0 (0.0%)	$\chi^2(2)=1.851$, p=.396
P1D	Nihilistic ideas about the non-existence of others	9 (9.7%)	15 (12.4%)	1 (5.6%)	$\chi^2(2)=0.958$, p=.620
P1D	Ideas of vanishing from the world	0 (0.0%)	2 (1.7%)	0 (0.0%)	$\chi^2(2)=1.851$, p=.396
P1D	Ideas of being part of a movie, computer game etc.	3 (3.2%)	0 (0.0%)	0 (0.0%)	$\chi^2(2)=4.543$, p=.103
P1D	Ideas that a part of the soul is separated	0 (0.0%)	1 (0.8%)	0 (0.0%)	$\chi^2(2)=0.921$, p=.631
P1D	Ideas of not being a human being	1 (1.1%)	0 (0.0%)	0 (0.0%)	$\chi^2(2)=1.501$, p=.472
P1D	Identity confusion (patient thinks s/he is someone else)	1 (1.1%)	0 (0.0%)	0 (0.0%)	$\chi^2(2)=1.501$, p=.472
P1D	Nihilistic ideas of being dead / dying	0 (0.0%)	2 (1.7%)	0 (0.0%)	$\chi^2(2)=1.851$, p=.396
P1D	Demarcation experiences	0 (0.0%)	1 (0.8%)	0 (0.0%)	$\chi^2(2)=0.921$, p=.631
P1D	Ideas of observing oneself from a birds-eye perspective	1 (1.1%)	1 (0.8%)	0 (0.0%)	$\chi^2(2)=0.208$, p=.901
P1D	Exaggerated ideas of guilt	4 (4.3%)	11 (9.1%)	0 (0.0%)	$\chi^2(2)=3.344$, p=.188
P1D	Ideas of jealousy	1 (1.1%)	3 (2.5%)	0 (0.0%)	$\chi^2(2)=0.954$, p=.621
P1D	Erotomanic ideas	0 (0.0%)	2 (1.7%)	0 (0.0%)	$\chi^2(2)=1.851$, p=.396
P1E	Ideas of being the center of non-negative attention	11 (11.8%)	15 (12.4%)	0 (0.0%)	$\chi^2(2)=2.480$, p=.289
P1E	Non-paranoid ideas of being especially addressed by random events (e.g. media)	12 (12.9%)	12 (9.9%)	0 (0.0%)	$\chi^2(2)=2.757$, p=.252
P2	General mistrust	7 (7.5%)	6 (5.0%)	1 (5.6%)	$\chi^2(2)=0.620$, p=.731
P2	Mistrust of friends	4 (4.3%)	4 (3.3%)	0 (0.0%)	$\chi^2(2)=0.853$, p=.653
P2	Paranoid ideas of reference (gazes of passers-by)	23 (24.7%)	33 (27.03%)	1 (5.6%)	$\chi^2(2)=3.990$, p=.136
P2	Paranoid ideas of reference involving friends / family	3 (3.2%)	7 (5.8%)	0 (0.0%)	$\chi^2(2)=1.714$, p=.424
P2	Ideas that others wish the patient ill	0 (0.0%)	1 (0.8%)	0 (0.0%)	$\chi^2(2)=0.921$, p=.631

SIPS No.	Content	Atheist (n=93)	Christian (n=121)	Other (n=18)	Statistics
P2	Ideas that others would exploit the patient	2 (2.2%)	2 (1.7%)	0 (0.0%)	$\chi^2(2)=0.419$, p=.811
P2	Increased vigilance due to feeling unsafe	4 (4.3%)	3 (3.3%)	0 (0.0%)	$\chi^2(2)=0.853$, p=.653
P2	Ideas of being threatened/observed by supernatural / invisible beings	2 (2.2%)	4 (3.3%)	1 (5.6%)	$\chi^2(2)=0.669$, p=.716
P2	Ideas of being observed anonymously (e.g. by cameras, internet etc.)	1 (0.0%)	0 (0.0%)	0 (0.0%)	$\chi^2(2)=1.501$, p=.472
P2	Ideas of being under surveillance (not solely observation)	3 (3.2%)	2 (1.7%)	0 (0.0%)	$\chi^2(2)=1.047$, p=.593
P2	Ideas of persecution	4 (4.3%)	4 (3.3%)	1 (5.6%)	$\chi^2(2)=00.287$, p=.866
P2	Ideas of being excluded	2 (2.2%)	3 (2.5%)	1 (5.6%)	$\chi^2(2)=0.706$, p=.703
P2	Ideas that others intend to harm the patient (not physically)	11 (11.8%)	16 (13.2%)	2 (11.1%)	$\chi^2(2)=0.128$, p=.983
P2	Ideas that others intend to poison the patient	0 (0.0%)	2 (1.7%)	0 (0.0%)	$\chi^2(2)=1.851$, p=.396
P2	Ideas that others intend to physically harm the patient	14 (15.1%)	11 (9.1%)	0 (0.0%)	$\chi^2(2)=4.301$, p=.116
P2	Ideas that supernatural beings intend to harm the patient	0 (0.0%)	1 (0.8%)	0 (0.0%)	$\chi^2(2)=0.921$, p=.631
P2	Ideas of being at risk of falling victim to terror attacks or similar	1 (1.1%)	1 (0.8%)	0 (0.0%)	$\chi^2(2)=0.208$, p=.901
P3	Grandiose ideas with respect to own (natural) abilities	4 (4.3%)	6 (5.0%)	0 (0.0%)	$\chi^2(2)=0.934$, p=.627
P3	Grandiose ideas of becoming famous	1 (1.1%)	3 (2.5%)	1 (5.6%)	$\chi^2(2)=1.562$, p=.458
P3	Grandiose ideas of being chosen to fulfil a greater plan (e.g., by God)	0 (0.0%)	3 (2.5%)	0 (0.0%)	$\chi^2(2)=2.788$, p=.248
P3	Grandiose ideas of becoming enlightened / a higher being	1 (1.1%)	0 (0.0%)	0 (0.0%)	$\chi^2(2)=1.501$, p=.472
P3	Grandiose ideas with respect to own supernatural abilities	1 (1.1%)	2 (1.7%)	1 (5.6%)	$\chi^2(2)=1.794$, p=.408
P3	Grandiose ideas of being a god / higher being	1 (1.1%)	0 (0.0%)	0 (0.0%)	$\chi^2(2)=1.501$, p=.472

Contents highlighted in grey have at least trend significance ($p<0.100$).
Numbers in **bold** signify cells with a standardized residuum ≥ |1.96|.

Supplementary Table 5b. Religion comparisons of the frequency of perceptual abnormalities/hallucinations (SIPS-P4) in CHR patients (N=232).

SIPS No.	Content	Atheist (n=93)	Christian (n=121)	Other (n=18)	Statistics
P4B	Acoustic illusions	0 (0.0%)	3 (2.5%)	0 (0.0%)	$\chi^2(2)$=2.788, p=.248
P4B	Hearing sounds made by non-living objects	8 (8.6%)	7 (5.8%)	3 (16.7%)	$\chi^2(2)$=2.747, p=.253
P4B	Hearing sounds made by living beings (humans, animals)	4 (4.3%)	3 (2.5%)	1 (5.6%)	$\chi^2(2)$=0.784, p=.676
P4B	Audible thoughts (not by others)	1 (1.1%)	0 (0.0%)	0 (0.0%)	$\chi^2(2)$=1.501, p=.472
P4B	Hearing one's own name being called	5 (5.4%)	7 (5.8%)	2 (11.1%)	$\chi^2(2)$=0.02, p=.637
P4B	Hearing of unintelligible voices (e.g. murmur)	5 (5.4%)	8 (6.6%)	0 (0.0%)	$\chi^2(2)$=1.310, p=.519
P4B	Hearing of dialoguing voices 9	2 (2.2%)	0 (0.0%)	0 (0.0%)	$\chi^2(2)$=30.015, p=.221
P4B	Hearing of commenting voices	1 (1.1%)	5 (4.1%)	0 (0.0%)	$\chi^2(2)$=2.469, p=.291
P4B	Hearing of imperative voices	1 (1.1%)	6 (5.0%)	0 (0.0%)	$\chi^2(2)$=3.317, p=.190
P4B	Hearing of insulting voices	3 (3.2%)	6 (5.0%)	0 (0.0%)	$\chi^2(2)$=1.211, p=.546
P4	Hearing of God's voice	1 (1.1%)	0 (0.0%)	0 (0.0%)	$\chi^2(2)$=1.501, p=.472
P4C	Sensing a presence	2 (2.2%)	11 (9.1%)	1 (5.6%)	$\chi^2(2)$=4.475, p=.107
P4C	Sensing the presence of deceased persons 8	1 (1.1%)	0 (0.0%)	0 (0.0%)	$\chi^2(2)$=1.501, p=.472
P4	Seeing moving shadows in the corner of the eye	9 (9.7%)	11 (9.1%)	0 (0.0%)	$\chi^2(2)$=1.864, p=.394
P4C	Visual illusions	1 (1.1%)	3 (2.5%)	1 (5.6%)	$\chi^2(2)$=1.562, p=.458
P4C	Illusions of objects moving	0 (0.0%)	1 (0.8%)	0 (0.0%)	$\chi^2(2)$=0.921, p=.631
P4C	Dysmorphophobic illusions	0 (0.0%)	2 (1.7%)	0 (0.0%)	$\chi^2(2)$=1.851, p=.396
P4C	Indistinct visual hallucinations	1 (1.1%)	4 (3.3%)	0 (0.0%)	$\chi^2(2)$=1.670, p=.434
P4C	Distinct visual hallucinations	6 (6.5%)	5 (4.1%)	0 (0.0%)	$\chi^2(2)$=1.598, p=.450
P4C	Seeing a person's shape	2 (2.2%)	6 (5.0%)	0 (0.0%)	$\chi^2(2)$=1.942, p=.379
P4C	Confusion of persons	0 (0.0%)	1 (0.8%)	0 (0.0%)	$\chi^2(2)$=0.921, p=.631
P4D	Sense of being touched	3 (3.2%)	2 (1.7%)	0 (0.0%)	$\chi^2(2)$=1.047, p=.593
P4D	Sense of changed body functions	1 (1.1%)	5 (4.1%)	0 (0.0%)	$\chi^2(2)$=2.469, p=.291
P4D	Non-painful bodily sensation	7 (7.5%)	13 (10.7%)	1 (5.6%)	$\chi^2(2)$=0.951, p=.622
P4D	Painful bodily sensation	4 (4.3%)	2 (1.7%)	2 (11.1%)	$\chi^2(2)$=4.549, p=.103
P4D	Sense of being infested by parasites	0 (0.0%)	1 (0.8%)	0 (0.0%)	$\chi^2(2)$=0.921, p=.631
P4D	Sensing normally non-sensible body functions (e.g. blood flow)	1 (1.1%)	0 (0.0%)	0 (0.0%)	$\chi^2(2)$=1.501, p=.472

SIPS No.	Content	Atheist (n=93)	Christian (n=121)	Other (n=18)	Statistics
P4E	Olfactory hallucinations	1 (1.1%)	4 (3.3%)	0 (0.0%)	$\chi^2(2)=1.670$, p=.434
P4E	Gustatory hallucinations	0 (0.0%)	1 (0.0%)	0 (0.0%)	$\chi^2(2)=0.921$, p=.631

Contents highlighted in grey have at least trend significance (p<0.100).
Numbers in **bold** signify cells with a standardized residuum ≥ |1.96|.

Supplementary Table 5c. Religion comparisons of the frequency of speech-disorganized symptoms (SIPS-P5) in CHR patients (N=232).

SIPS No.	Content	Atheist (n=93)	Christian (n=121)	Other (n=18)	Statistics
P5	Communication is vague	0 (0.0%)	2 (1.7%)	0 (0.0%)	$\chi^2(2)=1.851$, p=.396
P5	Poverty of speech	2 (2.2%)	1 (0.8%)	0 (0.0%)	$\chi^2(2)=0.978$, p=.613
P5	Neologisms	0 (0.0%)	2 (1.7%)	0 (0.0%)	$\chi^2(2)=1.851$, p=.396
P5	Extremely short, non-elaborative speech	0 (0.0%)	1 (0.8%)	0 (0.0%)	$\chi^2(2)=0.921$, p=.631
P5	Losing the thread of thoughts (self-experienced)	7 (7.5%)	5 (4.1%)	3 (16.7%)	$\chi^2(2)=4.360$, p=.113
P5	Losing the thread of thoughts (observed by others)	8 (8.6%)	6 (5.0%)	**4 (22.2%)**	$\chi^2(2)=6.680$, p=.035
P5	Derailment (self-experienced)	0 (0.0%)	1 (0.8%)	0 (0.0%)	$\chi^2(2)=0.921$, p=.631
P5	Derailment (observed by others)	3 (3.2%)	3 (2.5%)	1 (5.6%)	$\chi^2(2)=0.530$, p=.767
P5	Paralogia / alogia	0 (0.0%)	1 (0.8%)	0 (0.0%)	$\chi^2(2)=0.921$, p=.631
P5	Tangentiality (observed by others)	2 (2.2%)	5 (4.1%)	0 (0.0%)	$\chi^2(2)=1.313$, p=.519
P5	Thought blockage by intrusion (self-experienced)	0 (0.0%)	1 (0.8%)	0 (0.0%)	$\chi^2(2)=0.921$, p=.631
P5	Thought blockage by intrusion (observed by others)	1 (1.1%)	1 (0.8%)	0 (0.0%)	$\chi^2(2)=.208$, p=.901
P5	Thought intrusion (observed by others)	0 (0.0%)	1 (0.8%)	0 (0.0%)	$\chi^2(2)=0.921$ p=.631
P5	Circumstantial speech	1 (1.1%)	2 (1.7%)	**2 (11.1%)**	$\chi^2(2)=70.506$, p=.023
P5	Restricted / stereotyped thinking (observed by others)	0 (0.0%)	0 (0.0%)	**1 (5.6%)**	$\chi^2(2)=11.940$, p=.003
P5	Stilted or pedantic speech	1 (1.1%)	1 (0.8%)	0 (0.0%)	$\chi^2(2)=0.208$, p=.901
P5	Use of inadequate words	0 (0.0%)	1 (0.8%)	0 (0.0%)	$\chi^2(2)=0.921$, p=.631

Contents highlighted in grey have at least trend significance (p<0.100).
Numbers in **bold** signify cells with a standardized residuum ≥ |1.96|.

Supplementary Table 6a. Comparisons of the frequency of attenuated and transient delusional ideas (SIPS-P1, SIPS-P2, SIPS-P3) in CHR patients with and without mood disorder (N=232).

SIPS No.	Content	Mood disorder present (n=117)	Mood disorder absent (n=155)	Statistics
P1B	Thought insertion	4 (3.4%)	6 (5.2%)	χ²(1)=0.455, p=.537
P1B	Thought withdrawal	0 (0.0%)	2 (1.7%)	χ²(1)=2.052, p=.245
P1B	Audible thoughts (by others)	7 (6.0%)	5 (4.3%)	χ²(1)=0.316, p=.574
P1B	Experiences of mind being read	7 (6.0%)	10 (8.7%)	χ²(1)=0.628, p=.428
P1B	Thought broadcasting	5 (4.3%)	2 (1.7%)	χ²(1)=1.273, p=.446
P1B	Experience of being controlled by external forces	1 (0.9%)	4 (3.5%)	χ²(1)=1.893, p=.211
P1C	Ideas that strangers know something about patient	1 (0.9%)	1 (0.9%)	χ²(1)=0,0 p=1.0
P1C	Ideas that own thoughts could become real	0 (0.0%)	1 (0.9%)	χ²(1)=1.022, p=.496
P1C	Ideas that own actions would influence the surrounding	0 (0.0%)	1 (0.9%)	χ²(1)=1.022, p=.496
P1C	Belief in supernatural phenomena (ghosts, telepathy, afterlife, etc.)	**8 (6.8%)**	0 (0.0%)	χ²(1)=8.144, p=.007
P1C	Numbers have special meaning	0 (0.0%)	3 (2.6%)	χ²(1)=3.092, p=.120
P1C	Ideas that positive thoughts might cause bad things 1	0 (0.0%)	1 (0.9%)	χ²(1)=1.022, p=.496
P1C	Ideas of being directly affected by other persons feelings/actions 3	0 (0.0%)	2 (1.7%)	χ²(1)=2.052, p=.245
P1C	Belief that everything is connected	0 (0.0%)	1 (0.9%)	χ²(1)=1.022, p=.496
P1C	Belief in conspiracy theories	1 (0.9%)	0 (0.0%)	χ²(1)=0.987, p=1.0
P1C	Tendency to see relations between random events	1 (0.9%)	1 (0.9%)	χ²(1)=0 p=1.0
P1C	Ideas that others take over the patient's self/personality	0 (0.0%)	1 (0.9%)	χ²(1)=1.022, p=.496
P1D	Belief in fate	1 (0.9%)	0 (0.0%)	χ²(1)=0.987, p=1.0
P1C	Unusual religious ideas	1 (0.9%)	0 (0.0%)	χ²(1)=0.987, p=1.0
P1C	Unusual ideas about the world	1 (0.9%)	1 (0.9%)	χ²(1)=0, p=1.0
P1C	Ideas that things in the surrounding have a special meaning (no ideas of reference)	1 (0.9%)	2 (1.7%)	χ²(1)=0.335, p=.620
P1D	Unusual and unrealistic ideas about the own body	2 (1.7%)	4 (3.5%)	χ²(1)=0.720, p=.444
P1D	Hypochondriacal ideas	6 (5.1%)	8 (7.0%)	χ²(1)=0.342, p=.593
P1D	Ideas of being pregnant	1 (0.9%)	0 (0.0%)	χ²(1)=0.987, p=1.0
P1D	Nihilistic ideas about own non-existence	12 (10.3%)	5 (4.3%)	χ²(1)=2.982, p=.129
P1D	Ideas of the existence of another reality / universe	1 (0.9%)	1 (0.9%)	χ²(1)=0,0 p=1.0

SIPS No.	Content	Mood disorder present (n=117)	Mood disorder absent (n=155)	Statistics
P1D	Nihilistic ideas about the non-existence of others	15 (12.8%)	10 (8.7%)	$\chi^2(1)=1.026$ p=.311
P1D	Ideas of vanishing from the world	0 (0.0%)	2 (1.7%)	$\chi^2(1)=2.052$, p=.245
P1D	Ideas of being part of a movie, computer game etc.	1 (0.9%)	2 (1.7%)	$\chi^2(1)=1.368$, p=.554
P1D	Ideas that a part of the soul is separated	0 (0.0%)	1 (0.9%)	$\chi^2(1)=1.022$, p=.496
P1D	Ideas of not being a human being	0 (0.0%)	1 (0.9%)	$\chi^2(1)=1.022$, p=.496
P1D	Identity confusion (patient thinks s/he is someone else)	0 (0.0%)	1 (0.9%)	$\chi^2(1)=1.022$, p=.496
P1D	Nihilistic ideas of being dead/dying	2 (1.7%)	0 (0.0%)	$\chi^2(1)=1.983$, p=.498
P1D	Demarcation experiences	0 (0.0%)	1 (0.9%)	$\chi^2(1)=1.022$, p=.496
P1D	Ideas of observing oneself from a birds-eye perspective	2 (1.7%)	0 (0.0%)	$\chi^2(1)=1.983$, p=.498
P1D	Exaggerated ideas of guilt	5 (4.3%)	10 (8.7%)	$\chi^2(1)=1.875$, p=.192
P1D	Ideas of jealousy	2 (1.7%)	2 (1.7%)	$\chi^2(1)=0$, p=1.0
P1D	Erotomanic ideas	1 (0.9%)	1 (0.9%)	$\chi^2(1)=0$, p=1.0
P1E	Ideas of being the center of non-negative attention	11 (9.4%)	15 (13.0%)	$\chi^2(1)=0.773$, p=.379
P1E	Non-paranoid ideas of being especially addressed by random events (e.g., media)	10 (8.5%)	14 (12.2%)	$\chi^2(1)=0.823$, p=.364
P2	General mistrust	8 (6.8%)	6 (5.2%)	$\chi^2(1)=0.268$, p=.604
P2	Mistrust of friends	4 (3.4%)	4 (3.5%)	$\chi^2(1)=0.001$, p=1.0
P2	Paranoid ideas of reference (gazes of passers-by)	24 (20.5%)	33 (28.7%)	$\chi^2(1)=2.095$, p=.148
P2	Paranoid ideas of reference involving friends / family	1 (0.9%)	0 (0.0%)	$\chi^2(1)=1.745$, p=.213
P2	Ideas that others wish the patient ill	0 (0.0%)	2 (1.7%)	$\chi^2(1)=0.987$, p=1.0
P2	Ideas that others would exploit the patient	1 (0.9%)	3 (2.6%)	$\chi^2(1)=1.053$, p=.367
P2	Increased vigilance due to feeling unsafe	4 (3.4%)	4 (3.5%)	$\chi^2(1)=0.001$, p=1.0
P2	Ideas of being threatened/ observed by supernatural / invisible beings	5 (4.3%)	2 (1.7%)	$\chi^2(1)=1.273$, p=.446
P2	Ideas of being observed anonymously (e.g., by cameras, internet etc.)	0 (0.0%)	1 (0.9%)	$\chi^2(1)=1.022$, p=.496
P2	Ideas of being under surveillance (not solely observation)	3 (2.6%)	2 (1.7%)	$\chi^2(1)=0.187$, p=1.0

SIPS No.	Content	Mood disorder present (n=117)	Mood disorder absent (n=155)	Statistics
P2	Ideas of persecution	6 (5.1%)	3 (2.6%)	$\chi^2(1)=0.987$, p=.499
P2	Ideas of being excluded	2 (1.7%)	4 (3.5%)	$\chi^2(1)=0.720$, p=.444
P2	Ideas that others intend to harm the patient (not physically)	11 9.4%)	18 (15.7%)	$\chi^2(1)=2.072$, p=.150
P2	Ideas that others intend to poison the patient	2 (1.7%)	0 (0.0%)	$\chi^2(1)=1.983$, p=.498
P2	Ideas that others intend to physically harm the patient	14 (12.0%)	11 (9.6%)	$\chi^2(1)=0.348$, p=.555
P2	Ideas that supernatural beings intend to harm the patient	1 (0.9%)	0 (0.0%)	$\chi^2(1)=0.987$, p=1.0
P2	Ideas of being at risk of falling victim to terror attacks or similar	0 (0.0%)	2 (1.7%)	$\chi^2(1)=1.983$, p=.498
P3	Grandiose ideas with respect to own (natural) abilities	5 (4.3%)	5 (4.3%)	$\chi^2(1)=0.001$, p=1.0
P3	Grandiose ideas of becoming famous	3 (2.6%)	2 (1.7%)	$\chi^2(1)=0.187$, p=1.0
P3	Grandiose ideas of being chosen to fulfil a greater plan (e.g., by God)	1 (0.9%)	2 (1.7%)	$\chi^2(1)=0.355$, p=.620
P3	Grandiose ideas of becoming enlightened / a higher being	0 (0.0%)	1 (0.9%)	$\chi^2(1)=1.022$, p=.496
P3	Grandiose ideas with respect to own supernatural abilities	3 (2.6%)	1 (0.9%)	$\chi^2(1)=0.983$, p=.622
P3	Grandiose ideas of being a god / higher being	1 (0.9%)	0 (0.0%)	$\chi^2(1)=0.987$, p=1.0

Contents highlighted in grey have at least trend significance (p<0.100).
Numbers in **bold** signify cells with a standardized residuum ≥ |1.96|.

Supplementary Table 6b. Comparisons of the frequency of perceptual abnormalities/ hallucinations (SIPS-P4) in CHR patients with and without mood disorder (N=232).

SIPS No.	Content	Mood disorder present (n=117)	Mood disorder absent (n=115)	Statistics
P4B	Acoustic illusions	2 (1.7%)	1 (0.9%)	$\chi^2(1)=0.320$, p=1.0
P4B	Hearing sounds made by non-living objects	9 (7.7%)	9 (7.8%)	$\chi^2(1)=0.001$, p=1.0
P4B	Hearing sounds made by living beings (humans, animals)	4 (3.4%)	4 (3.5%)	$\chi^2(1)=0.001$, p=1.0
P4B	Audible thoughts (not by others)	1 (0.9%)	0 (0.0%)	$\chi^2(1)=0.987$, p=1.0
P4B	Hearing one's own name being called	6 (5.1%)	8 (7.0%)	$\chi^2(1)=0.342$, p=.593
P4B	Hearing of unintelligible voices (e.g., murmur)	8 (6.8%)	5 (4.3%)	$\chi^2(1)=0.680$, p=.410
P4B	Hearing of dialoguing voices	2 (1.7%)	0 (0.0%)	$\chi^2(1)=1.983$, p=.498
P4B	Hearing of commenting voices	1 (0.9%)	5 (4.3%)	$\chi^2(1)=2.809$, p=.118
P4B	Hearing of imperative voices	1 (0.9%)	6 (5.2%)	$\chi^2(1)=3.772$, p=.065
P4B	Hearing of insulting voices	4 (3.4%)	5 (4.3%)	$\chi^2(1)=0.134$, p=.747
P4	Hearing of God's voice	1 (0.9%)	0 (0.0%)	$\chi^2(1)=0.987$, p=1.0
P4C	Sensing a presence	10 (8.5%)	4 (3.5%)	$\chi^2(1)=2.628$, p=.105
P4C	Sensing the presence of deceased persons	0 (0.0%)	1 (0.9%)	$\chi^2(1)=1.022$, p=.496
P4	Seeing moving shadows in the corner of the eye	10 (8.5%)	10 (8.7%)	$\chi^2(1)=0.002$, p=.968
P4C	Visual illusions	5 (4.3%)	0 (0.0%)	$\chi^2(1)=5.023$, p=.060
P4C	Illusions of objects moving	0 (0.0%)	1 (0.9%)	$\chi^2(1)=1.022$, p=.496
P4C	Dysmorphophobic illusions	1 (0.9%)	1 (0.9%)	$\chi^2(1)=0$, p=1.0
P4C	Indistinct visual hallucinations	3 (2.6%)	2 (1.7%)	$\chi^2(1)=0.187$, p=1.0
P4C	Distinct visual hallucinations	5 (4.3%)	6 (5.2%)	$\chi^2(1)=0.114$, p=.735
P4C	Seeing a person's shape	4 (3.4%)	4 (3.5%)	$\chi^2(1)=0.001$ p=1.0
P4C	Confusion of persons	1 (0.9%)	0 (0.0%)	$\chi^2(1)=0.987$, p=1.0
P4D	Sense of being touched	3 (2.6%)	2 (1.7%)	$\chi^2(1)=0.187$, p=1.0
P4D	Sense of changed body functions	1 (0.9%)	5 (4.3%)	$\chi^2(1)=2.809$, p=.118
P4D	Non-painful bodily sensation	7 (6.0%)	14 (12.2%)	$\chi^2(1)=2.700$, p=.114
P4D	Painful bodily sensation	3 (2.6%)	5 (4.3%)	$\chi^2(1)=0.554$, p=.497
P4D	Sense of being infested by parasites	0 (0.0%)	1 (0.9%)	$\chi^2(1)=1.022$, p=.496
P4D	Sensing normally non-sensible body functions (e.g., blood flow)	0 (0.0%)	1 (0.9%)	$\chi^2(1)=1.022$, p=.496
P4E	Olfactory hallucinations	3 (2.6%)	2 (1.7%)	$\chi^2(1)=0.187$, p=1.0
P4	Gustatory hallucinations	0 (0.0%)	1 (0.9%)	$\chi^2(1)=1.022$, p=.496

Contents highlighted in grey have at least trend significance ($p<0.100$).
Numbers in **bold** signify cells with a standardized residuum ≥ |1.96|.

Supplementary Table 6c. Comparisons of the frequency of disorganized communication (SIPS-P5) in CHR patients with and without mood disorder (N=232).

SIPS No.	Content	Mood disorder present (n=117)	Mood disorder absent (n=115)	Statistics
P5	Communication is vague	1 (0.9%)	1 (0.9%)	$\chi^2(1)=0$, $p=1.0$
P5	Poverty of speech	3 (2.6%)	0 (0.0%)	$\chi^2(1)=2.987$, $p=.247$
P5	Neologisms	2 (1.7%)	0 (0.0%)	$\chi^2(1)=1.983$, $p=.498$
P5	Extremely short, non-elaborative speech	1 (0.9%)	0 (0.0%)	$\chi^2(1)=0.987$, $p=1.0$
P5	Losing the thread of thoughts (self-experienced)	7 (6.0%)	8 (7.0%)	$\chi^2(1)=0.091$, $p=.763$
P5	Losing the thread of thoughts (observed by others)	9 (7.7%)	9 (7.8%)	$\chi^2(1)=0.001$, $p=.970$
P5	Derailment (self-experienced)	0 (0.0%)	1 (0.9%)	$\chi^2(1)=1.022$, $p=.496$
P5	Derailment (observed by others)	1 (0.9%)	6 (5.2%)	$\chi^2(1)=3.772$, $p=.065$
P5	Paralogia / alogia	0 (0.0%)	1 (0.9%)	$\chi^2(1)=1.022$, $p=.496$
P5	Tangentiality (observed by others)	4 (3.4%)	3 (2.6%)	$\chi^2(1)=0.130$, $p=1.0$
P5	Thought blockage by intrusion (self-experienced)	1 (0.9%)	0 (0.0%)	$\chi^2(1)=0.987$, $p=1.0$
P5	Thought blockage by intrusion (observed by others)	2 (1.7%)	0 (0.0%)	$\chi^2(1)=1.983$, $p=.498$
P5	Thought intrusion (observed by others)	1 (0.9%)	0 (0.0%)	$\chi^2(1)=0.987$, $p=1.0$
P5	Circumstantial speech	3 (2.6%)	2 (1.7%)	$\chi^2(1)=0.187$, $p=1.0$
P5	Restricted / stereotyped thinking (observed by others)	1 (0.0%)	0 (0.0%)	$\chi^2(1)=0.987$, $p=1.0$
P5	Stilted or pedantic speech	1 (0.9%)	1 (0.9%)	$\chi^2(1)=0$, $p=1.0$
P5	Use of inadequate words	1 (0.9%)	0 (0.0%)	$\chi^2(1)=0.987$, $p=1.0$

Contents highlighted in grey have at least trend significance ($p<0.100$).
Numbers in **bold** signify cells with a standardized residuum ≥ |1.96|.

Supplementary Table 7a. Comparisons of the frequency of attenuated and transient delusional ideas (SIPS-P1, SIPS-P2, SIPS-P3) in CHR patients with and without anxiety disorder (N=232).

SIPS No.	Content	Anxiety disorder present (n=72)	Anxiety disorder absent (n=160)	Statistics
P1B	Thought insertion	4 (5.6%)	6 (3.8%)	$\chi^2(1)=0.392$, p=.505
P1B	Thought withdrawal	1 (1.4%)	1 (0.6%)	$\chi^2(1)=0.339$, p=.525
P1B	Audible thoughts (by others)	4 (5.6%)	8 (5.0%)	$\chi^2(1)=0.031$, p=1.0
P1B	Experiences of mind being read	9 (12.5%)	8 (5.0%)	$\chi^2(1)=4.113$, p=.056
P1B	Thought broadcasting	2 (2.8%)	5 (3.1%)	$\chi^2(1)=0.020$, p=1.0
P1B	Experience of being controlled by external forces	2 (2.8%)	3 (1.9%)	$\chi^2(1)=0.192$, p=.647
P1C	Ideas that strangers know something about patient	1 (1.4%)	1 (0.6%)	$\chi^2(1)=0.339$, p=.525
P1C	Ideas that own thoughts could become real	1 (1.4%)	0 (0.0%)	$\chi^2(1)=2.232$, p=.310
P1C	Ideas that own actions would influence the surrounding	1 (1.4%)	0 (0.0%)	$\chi^2(1)=2.232$, p=.310
P1C	Belief in supernatural phenomena (ghosts, telepathy, afterlife, etc.)	2 (2.8%)	6 (3.8%)	$\chi^2(1)=.141$, p=1.0
P1C	Numbers have special meaning	1 (1.4%)	2 (1.3%)	$\chi^2(1)=.008$, p=1.0
P1C	Ideas that positive thoughts might cause bad things	0 (0.0%)	1 (0.6%)	$\chi^2(1)=0.452$, p=1.0
P1C	Ideas of being directly affected by other persons feelings/actions	0 (0.0%)	2 (1.3%)	$\chi^2(1)=0.908$, p=1.0
P1C	Belief that everything is connected	1 (1.4%)	0 (0.0%)	$\chi^2(1)=2.232$, p=.310
P1C	Belief in conspiracy theories	0 (0.0%)	1 (0.6%)	$\chi^2(1)=0.452$, p=1.0
P1C	Tendency to see relations between random events	0 (1.3%)	2 (1.3%)	$\chi^2(1)=0.908$, p=1.0
P1C	Ideas that others take over the patient's self/personality	0 (0.0%)	1 (0.6%)	$\chi^2(1)=0.452$, p=1.0
P1D	Belief in fate	0 (0.0%)	1 (0.6%)	$\chi^2(1)=0.452$, p=1.0
P1C	Unusual religious ideas	1 (1.4%)	0 (0.0%)	$\chi^2(1)=2.232$, p=.310
P1C	Unusual ideas about the world	1 (1.4%)	1 (0.6%)	$\chi^2(1)=0.339$, p=.525
P1C	Ideas that things in the surrounding have a special meaning (no ideas of reference)	2 (2.8%)	1 (0.6%)	$\chi^2(1)=1.803$, p=.228
P1D	Unusual and unrealistic ideas about the own body	1 (1.4%)	5 (3.1%)	$\chi^2(1)=.594$, p=.669
P1D	Hypochondriacal ideas	6 (8.3%)	8 (5.0%)	$\chi^2(1)=.973$, p=.374
P1D	Ideas of being pregnant	0 (0.0%)	1 (0.6%)	$\chi^2(1)=0.452$, p=1.0
P1D	Nihilistic ideas about own non-existence	6 (8.3%)	11 (6.9%)	$\chi^2(1)=.156$, p=.786
P1D	Ideas of the existence of another reality / universe	1 (1.4%)	1 (0.6%)	$\chi^2(1)=0.339$, p=.525

SIPS No.	Content	Anxiety disorder present (n=72)	Anxiety disorder absent (n=160)	Statistics
P1D	Nihilistic ideas about the non-existence of others	9 (12.5%)	16 (10.0%)	$\chi^2(1)=.323$, p=.648
P1D	Ideas of vanishing from the world	1 (1.4%)	1 (0.6%)	$\chi^2(1)=0.339$, p=.525
P1D	Ideas of being part of a movie, computer game etc.	0 (0.0%)	3 (1.9%)	$\chi^2(1)=1.368$, p=.554
P1D	Ideas that a part of the soul is separated	0 (0.0%)	1 (0.5%)	$\chi^2(1)=0.452$, p=1.0
P1D	Ideas of not being a human being	1 (1.4%)	0 (0.0%)	$\chi^2(1)=2.232$, p=.310
P1D	Identity confusion (patient thinks s/he is someone else)	0 (0.0%)	1 (0.6%)	$\chi^2(1)=0.452$, p=1.0
P1D	Nihilistic ideas of being dead / dying	1 (1.4%)	1 (0.6%)	$\chi^2(1)=0.339$, p=.525
P1D	Demarcation experiences	0 (0.0%)	1 (0.6%)	$\chi^2(1)=0.452$, p=1.0
P1D	Ideas of observing oneself from a birds-eye perspective	0 (0.0%)	2 (1.3%)	$\chi^2(1)=0.908$, p=1.0
P1D	Exaggerated ideas of guilt	7 (9.7%)	8 (5.0%)	$\chi^2(1)=1.831$, p=.246
P1D	Ideas of jealousy	0	4 (2.5%)	$\chi^2(1)=1.832$, p=.313
P1D	Erotomanic ideas	1 (1.4%)	0 (0.6%)	$\chi^2(1)=0.339$, p=.525
P1E	Ideas of being the center of non-negative attention	3 (4.2%)	23 (14.4%)	$\chi^2(1)=5.200$, p=.024
P1E	Non-paranoid ideas of being especially addressed by random events (e.g., media)	5 (6.9%)	19 (11.9%)	$\chi^2(1)=1.302$, p=.352
P2	General mistrust	6 (8.3%)	8 (5.0%)	$\chi^2(1)=.973$, p=.374
P2	Mistrust of friends	2 (2.8%)	6 (3.8%)	$\chi^2(1)=.141$, p=1.0
P2	Paranoid ideas of reference (gazes of passers-by)	18 (25.0%)	39 (24.4%)	$\chi^2(1)=.010$, p=.919
P2	Paranoid ideas of reference involving friends / family	4 (5.6%)	6 (3.8%)	$\chi^2(1)=0.392$, p=.505
P2	Ideas that others wish the patient ill	0 (0.0%)	1 (0.6%)	$\chi^2(1)=0.452$, p=1.0
P2	Ideas that others would exploit the patient	2 (2.8%)	2 (1.3%)	$\chi^2(1)=0.684$, p=.590
P2	Increased vigilance due to feeling unsafe	3 (4.2%)	5 (3.1%)	$\chi^2(1)=0.162$, p=.706
P2	Ideas of being threatened/observed by supernatural / invisible beings	2 (2.8%)	5 (3.1%)	$\chi^2(1)=0.020$, p=1.0
P2	Ideas of being observed anonymously (e.g., by cameras, internet etc.)	0 (0.0%)	1 (0.6%)	$\chi^2(1)=0.452$, p=1.0
P2	Ideas of being under surveillance (not solely observation)	1 (1.4%)	4 (2.5%)	$\chi^2(1)=0.291$, p=1.0
P2	Ideas of persecution	2 (2.8%)	7 (4.4%)	$\chi^2(1)=0.340$, p=.724

SIPS No.	Content	Anxiety disorder present (n=72)	Anxiety disorder absent (n=160)	Statistics
P2	Ideas of being excluded	2 (2.8%)	4 (2.5%)	$\chi^2(1)=0.015$, p=1.0
P2	Ideas that others intend to harm the patient (not physically)	14 (19.4%)	15 (9.4%)	$\chi^2(1)=4.603$ p=.051
P2	Ideas that others intend to poison the patient	2 (2.8%)	0 (0.0%)	$\chi^2(1)=4.1483$, p=.095
P2	Ideas that others intend to physically harm the patient	8 (11.1%)	17 (10.6%)	$\chi^2(1)=0.012$, p=1.0
P2	Ideas that supernatural beings intend to harm the patient	0 (0.0%)	1 (0.6%)	$\chi^2(1)=0.482$, p=1.0
P2	Ideas of being at risk of falling victim to terror attacks or similar	1 (1.4%)	1 (0.6%)	$\chi^2(1)=0.339$, p=.525
P3	Grandiose ideas with respect to own (natural) abilities	0 (0.0%)	10 (6.3%)	$\chi^2(1)=4.703$, p=.034
P3	Grandiose ideas of becoming famous	0 (0.0%)	5 (3.1%)	$\chi^2(1)=2.300$, p=.328
P3	Grandiose ideas of being chosen to fulfil a greater plan (e.g., by God)	2 (2.8%)	1 (0.6%)	$\chi^2(1)=1.803$, p=.228
P3	Grandiose ideas of becoming enlightened / a higher being	0 (0.0%)	1 (0.6%)	$\chi^2(1)=0.452$, p=1.0
P3	Grandiose ideas with respect to own supernatural abilities	0 (0.0%)	4 (2.5%)	$\chi^2(1)=1.832$, p=.313
P3	Grandiose ideas of being a god / higher being	0 (0.0%)	1 (0.6%)	$\chi^2(1)=0.452$, p=1.0

Contents highlighted in grey have at least trend significance ($p<0.100$).
Numbers in **bold** signify cells with a standardized residuum ≥ |1.96|.

Supplementary Table 7b. Comparisons of the frequency of perceptual abnormalities/ hallucinations (SIPS-P4) in CHR patients with and without anxiety disorder (N=232).

SIPS No.	Content	Anxiety disorder present (n=72)	Anxiety disorder absent (n=160)	Statistics
P4B	Acoustic illusions	2 (2.8%)	1 (0.6%)	$\chi^2(1)=1.803$, p=.228
P4B	Hearing sounds made by non-living objects	7 (9.7%)	11 (6.9%)	$\chi^2(1)=0.562$, p=.439
P4B	Hearing sounds made by living beings (humans, animals)	2 (2.8%)	6 (3.8%)	$\chi^2(1)=0.141$, p=1.0
P4B	Audible thoughts (not by others)	0 (0.0%)	1 (0.6%)	$\chi^2(1)=0.452$, p=1.0
P4B	Hearing one's own name being called	2 (2.8%)	12 (7.5)	$\chi^2(1)=1.953$, p=.236
P4B	Hearing of unintelligible voices (e.g., murmur)	**8 (11.1%)**	5 (3.1%)	$\chi^2(1)=5.987$, p=.026
P4B	Hearing of dialoguing voices	0 (0.0%)	2 (1.3%)	$\chi^2(1)=00.908$, p=1.0
P4B	Hearing of commenting voices	2 (2.8%)	4 (2.5%)	$\chi^2(1)=0.015$, p=1.0
P4B	Hearing of imperative voices	4 (5.6%)	3 (1.9%)	$\chi^2(1)=2.299$, p=.208
P4B	Hearing of insulting voices	3 (4.2%)	6 (3.8%)	$\chi^2(1)=0.023$, p=1.0
P4	Hearing of God's voice	0 (0.0%)	1 (0.6%)	$\chi^2(1)=0.452$, p=1.0
P4C	Sensing a presence	5 (6.9%)	9 (5.6%)	$\chi^2(1)=0.152$, p=.767
P4C	Sensing the presence of deceased persons	0 (0.0%)	1 (0.6%)	$\chi^2(1)=0.452$, p=1.0
P4	Seeing moving shadows in the corner of the eye	4 (5.6%)	16 (10.0%)	$\chi^2(1)=1.245$, p=.321
P4C	Visual illusions	1 (1.4%)	4 (2.5%)	$\chi^2(1)=0.291$, p=1.0
P4C	Illusions of objects moving	0 (0.0%)	1 (0.6%)	$\chi^2(1)=0.452$, p=1.0
P4C	Dysmorphophobic illusions	1 (1.4%)	1 (0.6%)	$\chi^2(1)=0.339$, p=.525
P4C	Indistinct visual hallucinations	3 (4.2%)	2 (1.3%)	$\chi^2(1)=2.003$, p=.175
P4C	Distinct visual hallucinations	6 (8.3%)	5 (3.1%)	$\chi^2(1)=20.987$, p=.100
P4C	Seeing a person's shape	3 (4.2%)	5 (3.1%)	$\chi^2(1)=0.162$ p=.706
P4C	Confusion of persons	0 (0.0%)	1 (0.6%)	$\chi^2(1)=0.452$, p=1.0
P4D	Sense of being touched	1 (1.4%)	4 (2.5%)	$\chi^2(1)=0.291$, p=1.0
P4D	Sense of changed body functions	3 (4.2%)	3 (1.9%)	$\chi^2(1)=1.035$, p=.378
P4D	Non-painful bodily sensation	7 (9.7%)	14 (8.8%)	$\chi^2(1)=0.057$, p=.808
P4D	Painful bodily sensation	1 (1.4%)	7 (4.4%)	$\chi^2(1)=1.330$, p=.440
P4D	Sense of being infested by parasites	1 (1.4%)	0 (0.0%)	$\chi^2(1)=2.232$, p=.310
P4D	Sensing normally non-sensible body functions (e.g. blood flow)	0 (0.0%)	1 (0.6%)	$\chi^2(1)=0.452$, p=1.0
P4E	Olfactory hallucinations	2 (2.8%)	3 (1.9%)	$\chi^2(1)=0.192$, p=.647
P4	Gustatory hallucinations	0 (0.0%)	1 (0.6%)	$\chi^2(1)=0.452$, p=1.0

Contents highlighted in grey have at least trend significance (p<0.100).
Numbers in **bold** signify cells with a standardized residuum ≥ |1.96|.

Supplementary Table 7c. Comparisons of the frequency of signs of disorganized communication (SIPS-P5) in CHR patients with and without anxiety disorder (N=232).

SIPS No.	Content	Anxiety disorders present (n=72)	Anxiety disorder absent (n=160)	Statistics
P5	Communication is vague	1 (1.4%)	1 (0.6%)	$\chi^2(1)=0.339$, p=.525
P5	Poverty of speech	1 (1.4%)	2 (1.3%)	$\chi^2(1)=.008$, p=1.0
P5	Neologisms	1 (1.4%)	1 (0.6%)	$\chi^2(1)=0.339$, p=.525
P5	Extremely short, non-elaborative speech	1 (1.4%)	0 (0.0%)	$\chi^2(1)=2.232$, p=.310
P5	Losing the thread of thoughts (self-experienced)	6 (8.3%)	9 (5.6%)	$\chi^2(1)=0.602$, p=.564
P5	Losing the thread of thoughts (observed by others)	7 (9.7%)	11 (6.9%)	$\chi^2(1)=0.562$, p=.439
P5	Derailment (self-experienced)	1 (1.4%)	0	$\chi^2(1)=2.232$, p=.310
P5	Derailment (observed by others)	1 (1.4%)	6 (3.8%)	$\chi^2(1)=0.941$, p=.441
P5	Paralogia / alogia	1 (1.4%)	0 (0.0%)	$\chi^2(1)=2.232$, p=.310
P5	Tangentiality (observed by others)	3 (4.2%)	4 (2.5%)	$\chi^2(1)=0.471$, p=.680
P5	Thought blockage by intrusion (self-experienced)	0 (0.0%)	1 (0.6%)	$\chi^2(1)=0.452$, p=1.0
P5	Thought blockage by intrusion (observed by others)	0 (0.0%)	2 (1.3%)	$\chi^2(1)=0.908$, p=1.0
P5	Thought intrusion (observed by others)	0 (0.0%)	1 (0.6%)	$\chi^2(1)=0.452$, p=1.0
P5	Circumstantial speech	1 (1.4%)	4 (2.5%)	$\chi^2(1)=0.291$, p=1.0
P5	Restricted / stereotyped thinking (observed by others)	0 (0.0%)	1 (0.6%)	$\chi^2(1)=0.452$, p=1.0
P5	Stilted or pedantic speech	0 (0.0%)	2 (1.3%)	$\chi^2(1)=0.908$, p=1.0
P5	Use of inadequate words	0 (0.0%)	1 (0.6%)	$\chi^2(1)=0.452$, p=1.0

Contents highlighted in grey have at least trend significance ($p<0.100$).
Numbers in **bold** signify cells with a standardized residuum ≥ |1.96|.

Supplementary Table 8a. Comparisons of the frequency of attenuated and transient delusional ideas (SIPS-P1, SIPS-P2, SIPS-P3) in CHR patients with and without other disorders (N=232).

SIPS No.	Content	Other disorders present (n=21)	Other disorders absent (n=211)	Statistics
P1B	Thought insertion	2 (9.5%)	8 (3.6%)	$\chi^2(1)$=1.522, p=.226
P1B	Thought withdrawal	0 (0.0%)	2 (0.9%)	$\chi^2(1)$=0.201, p=1.0
P1B	Audible thoughts (by others)	0 (0.0%)	12 (5.7%)	$\chi^2(1)$=1.259, p=.608
P1B	Experiences of mind being read	2 (9.5%)	15 (7.1%)	$\chi^2(1)$=0.164, p=.657
P1B	Thought broadcasting	0 (0.0%)	7 (3.3%)	$\chi^2(1)$=0.718, p=1.0
P1B	Experience of being controlled by external forces	**2 (9.5%)**	3 (1.4%)	$\chi^2(1)$=5.945, p=.066
P1C	Ideas that strangers know something about patient	0 (0.0%)	2 (0.9%)	$\chi^2(1)$=0.201, p=1.0
P1C	Ideas that own thoughts could become real	0 (0.0%)	1 (0.5%)	$\chi^2(1)$=0.100, p=1.0
P1C	Ideas that own actions would influence the surrounding	0 (0.0%)	1 (0.5%)	$\chi^2(1)$=0.100, p=1.0
P1C	Belief in supernatural phenomena (ghosts, telepathy, afterlife, power of the universe etc.)	0 (0.0%)	8 (3.8%)	$\chi^2(1)$=0.825, p=1.0
P1C	Numbers have special meaning	0 (0.0%)	3 (1.4%)	$\chi^2(1)$=0.302, p=1.0
P1C	Ideas that positive thoughts might cause bad things	0 (0.0%)	1 (0.5%)	$\chi^2(1)$=0.100, p=1.0
P1C	Ideas of being directly affected by other persons feelings/actions	0 (0.0%)	2 (0.9%)	$\chi^2(1)$=.201, p=1.0
P1C	Belief that everything is connected	0 (0.0%)	1 (0.5%)	$\chi^2(1)$=0.100, p=1.0
P1C	Belief in conspiracy theories	0 (0.0%)	1 (0.5%)	$\chi^2(1)$=0.100, p=1.0
P1C	Tendency to see relations between random events	0 (0.0%)	2 (0.9%)	$\chi^2(1)$=.201, p=1.0
P1C	Ideas that others take over the patient's self/personality	0 (0.0%)	1 (0.5%)	$\chi^2(1)$=0.100, p=1.0
P1D	Belief in fate	0 (0.0%)	1 (0.6%)	$\chi^2(1)$=0.100, p=1.0
P1C	Unusual religious ideas	**1 (4.8%)**	1 (0.6%)	$\chi^2(1)$=100.091, p=.091
P1C	Unusual ideas about the world	0 (0.0%)	2 (0.9%)	$\chi^2(1)$=0.201, p=1.0
P1C	Ideas that things in the surrounding have a special meaning (no ideas of reference)	1 (4.8%)	2 (0.9%)	$\chi^2(1)$=2.177, p=.249
P1D	Unusual and unrealistic ideas about the own body	0 (0.0%)	6 (2.8%)	$\chi^2(1)$=0.613, p=1.0
P1D	Hypochondriacal ideas	2 (9.5%)	12 (5.7%)	$\chi^2(1)$=.496, p=.368
P1D	Ideas of being pregnant	0 (0.0%)	1 (0.6%)	$\chi^2(1)$=0.100, p=1.0
P1D	Nihilistic ideas about own non-existence	1 (4.8%)	16 (7.6%)	$\chi^2(1)$=0.224, p=1.0
P1D	Ideas of the existence of another reality / universe	0 (0.0%)	2 (0.9%)	$\chi^2(1)$=0.201, p=1.0

SIPS No.	Content	Other disorders present (n=21)	Other disorders absent (n=211)	Statistics
P1D	Nihilistic ideas about the non-existence of others	0 (0.0%)	25 (11.8%)	$\chi^2(1)$=2.789., p=.140
P1D	Ideas of vanishing from the world	0 (0.0%)	2 (0.9%)	$\chi^2(1)$=0.201, p=1.0
P1D	Ideas of being part of a movie, computer game etc.	0 (0.0%)	3 (1.4%)	$\chi^2(1)$=0.302, p=1.0
P1D	Ideas that a part of the soul is separated	0 (0.0%)	1 (0.5%)	$\chi^2(1)$=0.100, p=1.0
P1D	Ideas of not being a human being	0 (0.0%)	1 (0.5%)	$\chi^2(1)$=0.100, p=1.0
P1D	Identity confusion (patient thinks s/he is someone else)	0 (0.0%)	1 (0.5%)	$\chi^2(1)$=0.100, p=1.0
P1D	Nihilistic ideas of being dead / dying	1 (4.8%)	1 (0.5%)	$\chi^2(1)$=4.109, p=.173
P1D	Demarcation experiences	0 (0.0%)	1 (0.5%)	$\chi^2(1)$=0.100, p=1.0
P1D	Ideas of observing oneself from a birds-eye perspective	1 (4.8%)	1 (0.5%)	$\chi^2(1)$=4.109, p=.173
P1D	Exaggerated ideas of guilt	1 (4.8%)	14 (6.6%)	$\chi^2(1)$=0.111, p=1.0
P1D	Ideas of jealousy	0 (0.0%)	4 (1.9%)	$\chi^2(1)$=0.405, p=1.0
P1D	Erotomanic ideas	0 (0.0%)	2 (0.9%)	$\chi^2(1)$=0.201., p=1.0
P1E	Ideas of being the center of non-negative attention	1 (4.8%)	25 (11.8%)	$\chi^2(1)$=0.964, p=.482
P1E	Non-paranoid ideas of being especially addressed by random events (e.g. media)	3 (14.3%)	21 (10.0%)	$\chi^2(1)$=0.387, p=.463
P2	General mistrust	1 (4.8%)	13 (6.2%)	$\chi^2(1)$=0.066, p=1.0
P2	Mistrust of friends	0 (0.0%)	8 (3.8%)	$\chi^2(1)$=0.825., p=1.0
P2	Paranoid ideas of reference (gazes of passers-by)	6 (28.6%)	51 (24.2%)	$\chi^2(1)$=0.200, p=.655
P2	Paranoid ideas of reference involving friends / family	**3 (14.2%)**	7 (3.3%)	$\chi^2(1)$=5.571, p=.051
P2	Ideas that others wish the patient ill	0 (0.0%)	1 (0.5%)	$\chi^2(1)$=0.100, p=1.0
P2	Ideas that others would exploit the patient	1 (4.8%)	3 (1.4%)	$\chi^2(1)$=1.258, p=.318
P2	Increased vigilance due to feeling unsafe	1 (4.8%)	7 (3.3%)	$\chi^2(1)$=0.120, p=.538
P2	Ideas of being threatened/observed by supernatural / invisible beings	0 (0.0%)	7 (3.3%)	$\chi^2(1)$=0.718, p=1.0
P2	Ideas of being observed anonymously (e.g. by cameras, internet etc.)	0 (0.0%)	1 (0.5%)	$\chi^2(1)$=0.100, p=1.0
P2	Ideas of being under surveillance (not solely observation)	0 (0.0%)	5 (2.4%)	$\chi^2(1)$=0.509, p=1.0
P2	Ideas of persecution	1 (4.8%)	8 (3.8%)	$\chi^2(1)$=0.048., p=.581
P2	Ideas of being excluded	0 (0.0%)	6 (2.8%)	$\chi^2(1)$=0.613, p=1.0

SIPS No.	Content	Other disorders present (n=21)	Other disorders absent (n=211)	Statistics
P2	Ideas that others intend to harm the patient (not physically)	2 (9.5%)	27 (12.8%)	$\chi^2(1)=0.187$, p=1.0
P2	Ideas that others intend to poison the patient	0 (0.0%)	2 (0.9%)	$\chi^2(1)=0.201$, p=1.0
P2	Ideas that others intend to physically harm the patient	3 (14.3%)	22 (10.4%)	$\chi^2(1)=0.296$, p=.481
P2	Ideas that supernatural beings intend to harm the patient	0 (0.0%)	1 (0.5%)	$\chi^2(1)=0.100$, p=1.0
P2	Ideas of being at risk of falling victim to terror attacks or similar	0 (0.0%)	2 (0.9%)	$\chi^2(1)=0.201$, p=1.0
P3	Grandiose ideas with respect to own (natural) abilities	1 (4.8%)	9 (4.3%)	$\chi^2(1)=0.011$, p=1.0
P3	Grandiose ideas of becoming famous	1 (4.8%)	4 (1.9%)	$\chi^2(1)=0.744$, p=380
P3	Grandiose ideas of being chosen to fulfil a greater plan (e.g., by God)	1 (4.8%)	2 (0.9%)	$\chi^2(1)=.2.177$ p=.249
P3	Grandiose ideas of becoming enlightened / a higher being	0 (0.0%)	1 (0.5%)	$\chi^2(1)=0.100$, p=1.0
P3	Grandiose ideas with respect to own supernatural abilities	0 (0.0%)	4 (1.9%)	$\chi^2(1)=0.405$, p=1.0
P3	Grandiose ideas of being a god / higher being	0 (0.0%)	1 (0.5%)	$\chi^2(1)=0.100$, p=1.0

Contents highlighted in grey have at least trend significance ($p<0.100$).
Numbers in **bold** signify cells with a standardized residuum ≥ |1.96|.

Supplementary Table 8b. Comparisons of the frequency of perceptual abnormalities/ hallucinations (SIPS-P4) in CHR patients with and without other disorders (N=232).

SIPS No.	Content	Other disorders present (n=21)	Other disorders absent (n=211)	Statistics
P4B	Acoustic illusions	0 (0.0%)	3 (1.4%)	$\chi^2(1)=0.302$, p=1.0
P4B	Hearing sounds made by non-living objects	0 (0.0%)	18 (8.5%)	$\chi^2(1)=1.942$, p=.383
P4B	Hearing sounds made by living beings (humans, animals)	0 (0.0%)	8 (3.8%)	$\chi^2(1)=0.825$, p=1.0
P4B	Audible thoughts (not by others)	0 (0.0%)	1 (0.5%)	$\chi^2(1)=0.100$, p=1.0
P4B	Hearing one's own name being called	0 (0.0%)	14 (6.6%)	$\chi^2(1)=1.483$, p=.622
P4B	Hearing of unintelligible voices (e.g., murmur)	2 (9.5%)	11 (5.2%)	$\chi^2(1)=0.671$, p=.333
P4B	Hearing of dialoguing voices	0 (0.0%)	2 (0.9%)	$\chi^2(1)=0.201$, p=1.0
P4B	Hearing of commenting voices	0 (0.0%)	6 (2.8%)	$\chi^2(1)=0.613$, p=1.0
P4B	Hearing of imperative voices	1 (4.8%)	6 (2.8%)	$\chi^2(1)=0.240$, p=.490
P4B	Hearing of insulting voices	1 (4.8%)	8 (3.8%)	$\chi^2(1)=0.048$, p=.581
P4	Hearing of God's voice	0 (0.0%)	1 (0.5%)	$\chi^2(1)=0.100$, p=1.0
P4C	Sensing a presence	2 (9.5%)	12 (5.7%)	$\chi^2(1)=0.496$, p=.368
P4C	Sensing the presence of deceased persons	0 (0.0%)	1 (0.5%)	$\chi^2(1)=0.100$, p=1.0
P4	Seeing moving shadows in the corner of the eye	2 (9.5%)	18 (8.5%)	$\chi^2(1)=0.024$, p=.699
P4C	Visual illusions	0 (0.0%)	5 (2.4%)	$\chi^2(1)=0.509$, p=1.0
P4C	Illusions of objects moving	0 (0.0%)	1 (0.5%)	$\chi^2(1)=0.100$, p=1.0
P4C	Dysmorphophobic illusions	0 (0.0%)	2 (0.9%)	$\chi^2(1)=0.201$, p=1.0
P4C	Indistinct visual hallucinations	1 (4.8%)	4 (1.9%)	$\chi^2(1)=0.744$, p=.380
P4C	Distinct visual hallucinations	0 (0.0%)	11 (5.2%)	$\chi^2(1)=1.149$, p=.605
P4C	Seeing a person's shape	0 (0.0%)	8 (3.8%)	$\chi^2(1)=0.825$, p=1.0
P4C	Confusion of persons	0 (0.0%)	1 (0.5%)	$\chi^2(1)=0.100$, p=1.0
P4D	Sense of being touched	1 (4.8%)	4 (1.9%)	$\chi^2(1)=0.744$, p=.380
P4D	Sense of changed body functions	0 (0.0%)	6 (2.8%)	$\chi^2(1)=0.613$, p=1.0
P4D	Non-painful bodily sensation	4 (19.0%)	17 (8.1%)	$\chi^2(1)=2.802$, p=.106
P4D	Painful bodily sensation	**3 (14.3%)**	5 (2.4%)	$\chi^2(1)=8.145$, p=.027
P4D	Sense of being infested by parasites	0 (0.0%)	1 (0.5%)	$\chi^2(1)=0.100$, p=1.0
P4D	Sensing normally non-sensible body functions (e.g. blood flow)	0 (0.0%)	1 (0.5%)	$\chi^2(1)=0.100$, p=1.0
P4E	Olfactory hallucinations	0 (0.0%)	5 (2.4%)	$\chi^2(1)=0.509$, p=1.0
P4	Gustatory hallucinations	0 (0.0%)	1 (0.5%)	$\chi^2(1)=0.100$, p=1.0

Contents highlighted in grey have at least trend significance (p<0.100).
Numbers in **bold** signify cells with a standardized residuum ≥ |1.96|.

Supplementary Table 8c. Comparisons of the frequency of signs of disorganized communication (SIPS-P5) in CHR patients with and without other disorders (N=232).

SIPS No.	Content	Other disorders present (n=21)	Other disorders absent (n=211)	Statistics
P5	Communication is vague	0 (0.0%)	2 (0.9%)	$\chi^2(1)=0.201$, p=1.0
P5	Poverty of speech	0 (0.0%)	3 (1.4%)	$\chi^2(1)=0.302$, p=1.0
P5	Neologisms	1 (4.8%)	1 (0.5%)	$\chi^2(1)=4.109$, p=.173
P5	Extremely short, non-elaborative speech	0 (0.0%)	1 (0.5%)	$\chi^2(1)=0.100$, p=1.0
P5	Losing the thread of thoughts (self-experienced)	1 (4.8%)	14 (6.6%)	$\chi^2(1)=0.111$, p=1.0
P5	Losing the thread of thoughts (observed by others)	1 (4.8%)	17 (8.1%)	$\chi^2(1)=0.290$, p=1.0
P5	Derailment (self-experienced)	0 (0.0%)	1 (0.5%)	$\chi^2(1)=0.100$, p=1.0
P5	Derailment (observed by others)	1 (4.8%)	6 (2.8%)	$\chi^2(1)=0.240$, p=.490
P5	Paralogia / alogia	0 (0.0%)	1 (0.5%)	$\chi^2(1)=0.100$, p=1.0
P5	Tangentiality (observed by others)	2 (9.5%)	5 (2.4%)	$\chi^2(1)=3.341$, p=.124
P5	Thought blockage by intrusion (self-experienced)	0 (0.0%)	1 (0.5%)	$\chi^2(1)=0.100$, p=1.0
P5	Thought blockage by intrusion (observed by others)	1 (4.8%)	1 (0.5%)	$\chi^2(1)=4.109$, p=.173
P5	Thought intrusion (observed by others)	0 (0.0%)	1 (0.5%)	$\chi^2(1)=0.100$, p=1.0
P5	Circumstantial speech	0 (0.0%)	5 (2.4%)	$\chi^2(1)=0.509$, p=1.0
P5	Restricted / stereotyped thinking (observed by others)	0 (0.0%)	1 (0.5%)	$\chi^2(1)=0.100$, p=1.0
P5	Stilted or pedantic speech	0 (0.0%)	2 (0.9%)	$\chi^2(1)=0.201$, p=1.0
P5	Use of inadequate words	0 (0.0%)	1 (0.5%)	$\chi^2(1)=0.100$, p=1.0

Contents highlighted in grey have at least trend significance (p<0.100).
Numbers in **bold** signify cells with a standardized residuum ≥ |1.96|.

Supplementary Table 9a. Comparisons of the frequency of attenuated and transient delusional ideas (SIPS-P1, SIPS-P2, SIPS-P3) in CHR patients with and without somatization disorder (N=232).

SIPS No.	Content	Somatization disorder present (n=13)	Somatization disorder absent (n=219)	Statistics
P1B	Thought insertion	2 (15.4%)	8 (3.7%)	$\chi^2(1)=4.095$, p=.101
P1B	Thought withdrawal	**1 (7.7%)**	1 (0.5%)	$\chi^2(1)=7.518$, p=.109
P1B	Audible thoughts (by others)	1 (7.7%)	11 (5.0%)	$\chi^2(1)=0.178$, p=.508
P1B	Experiences of mind being read	1 (7.7%)	16 (7.3%)	$\chi^2(1)=0.003$, p=1.0
P1B	Thought broadcasting	0 (0.0%)	7 (3.2%)	$\chi^2(1)=0.428$, p=1.0
P1B	Experience of being controlled by external forces	1 (7.7%)	4 (1.8%)	$\chi^2(1)=2.002$, p=.252
P1	Ideas that strangers know something about patient	0 (0.0%)	2 (0.9%)	$\chi^2(1)=0.120$, p=1.0
P1C	Ideas that own thoughts could become real	0 (0.0%)	1 (0.5%)	$\chi^2(1)=0.060$, p=1.0
P1C	Ideas that own actions would influence the surrounding	0 (0.0%)	1 (0.5%)	$\chi^2(1)=0.060$, p=1.0
P1C	Belief in supernatural phenomena (ghosts, telepathy, afterlife, power of the universe etc.)	0 (0.0%)	8 (3.7%)	$\chi^2(1)=0.492$, p=1.0
P1C	Numbers have special meaning	0 (0.0%)	3 (1.4%)	$\chi^2(1)=0.180$, p=1.0
P1C	Ideas that positive thoughts might cause bad things	0 (0.0%)	1 (0.5%)	$\chi^2(1)=0.060$, p=1.0
P1C	Ideas of being directly affected by other persons feelings/actions	0 (0.0%)	2 (0.5%)	$\chi^2(1)=0.120$, p=1.0
P1C	Belief that everything is connected	0 (0.0%)	1 (0.5%)	$\chi^2(1)=0.060$, p=1.0
P1C	Belief in conspiracy theories	0 (0.0%)	1 (0.5%)	$\chi^2(1)=0.060$, p=1.0
P1C	Tendency to see relations between random events	0 (0.0%)	1 (0.5%)	$\chi^2(1)=0.120$, p=1.0
P1C	Ideas that others take over the patient's self/personality	0 (0.0%)	1 (0.5%)	$\chi^2(1)=0.060$, p=1.0
P1D	Belief in fate	0 (0.0%)	1 (0.5%)	$\chi^2(1)=0.060$, p=1.0
P1C	Unusual religious ideas	0 (0.0%)	1 (0.5%)	$\chi^2(1)=0.060$, p=1.0
P1C	Unusual ideas about the world	0 (0.0%)	2 (0.9%)	$\chi^2(1)=0.120$, p=1.0
P1C	Ideas that things in the surrounding have a special meaning (no ideas of reference)	0 (0.0%)	3 (1.4%)	$\chi^2(1)=0.180$, p=1.0
P1D	Unusual and unrealistic ideas about the own body	0 (0.0%)	6 (2.7%)	$\chi^2(1)=0.366$, p=1.0
P1D	Hypochondriacal ideas	2 (15.4%)	12 (5.5%)	$\chi^2(1)=2.123$, p=.180
P1D	Ideas of being pregnant	**1 (7.7%)**	0 (0.0%)	$\chi^2(1)=16.919$, p=.056
P1D	Nihilistic ideas about own non-existence	0 (0.0%)	17 (7.8%)	$\chi^2(1)=1.089$, p=.606

SIPS No.	Content	Somatization disorder present (n=13)	Somatization disorder absent (n=219)	Statistics
P1D	Ideas of the existence of another reality / universe	0 (0.0%)	2 (0.9%)	$\chi^2(1)=0.120$, p=1.0
P1D	Nihilistic ideas about the non-existence of others	1 (7.7%)	24 (11.0%)	$\chi^2(1)=0.136$, p=1.0
P1D	Ideas of vanishing from the world	0 (0.0%)	2 (0.9%)	$\chi^2(1)=0.120$, p=1.0
P1D	Ideas of being part of a movie, computer game etc.	0 (0.0%)	3 (0.5%)	$\chi^2(1)=0.180$, p=1.0
P1D	Ideas that a part of the soul is separated	0 (0.0%)	1 (0.5%)	$\chi^2(1)=0.060$, p=1.0
P1D	Ideas of not being a human being	0 (0.0%)	1 (0.5%)	$\chi^2(1)=0.060$, p=1.0
P1D	Identity confusion (patient thinks s/he is someone else)	0 (0.0%)	1 (0.5%)	$\chi^2(1)=0.060$, p=1.0
P1D	Nihilistic ideas of being dead / dying	0 (0.0%)	2 (0.9%)	$\chi^2(1)=0.120$, p=1.0
P1D	Demarcation experiences	0 (0.0%)	1 (0.5%)	$\chi^2(1)=0.060$, p=1.0
P1D	Ideas of observing oneself from a birds-eye perspective	0 (0.0%)	2 (0.9%)	$\chi^2(1)=0.120$, p=1.0
P1D	Exaggerated ideas of guilt	1 (7.7%)	14 (6.4%)	$\chi^2(1)=0.034$, p=.059
P1D	Ideas of jealousy	1 (7.7%)	3 (1.4%)	$\chi^2(1)=2.895$, p=.207
P1D	Erotomanic ideas	0 (0.0%)	2 (0.9%)	$\chi^2(1)=0.120$, p=1.0
P1E	Ideas of being the center of non-negative attention	1 (7.7%)	25 (11.4%)	$\chi^2(1)=0.171$, p=1.0
P1E	Non-paranoid ideas of being especially addressed by random events (e.g. media)	1 (7.7%)	23 (10.5%)	$\chi^2(1)=0.104$, p=1.0
P2	General mistrust	0 (0.0%)	14 (6.4%)	$\chi^2(1)=0.884$, p=1.0
P2	Mistrust of friends	0 (0.0%)	8 (3.7%)	$\chi^2(1)=0.492$, p=1.0
P2	Paranoid ideas of reference (gazes of passers-by)	0 (0.0%)	57 (26.0%)	$\chi^2(1)=4.486$, p=.042
P2	Paranoid ideas of reference involving friends / family	0 (0.0%)	10 (4.6%)	$\chi^2(1)=0.620$, p=1.0
P2	Ideas that others wish the patient ill	0 (0.0%)	1 (0.5%)	$\chi^2(1)=0.060$, p=1.0
P2	Ideas that others would exploit the patient	0 (0.0%)	4 (1.8%)	$\chi^2(1)=0.242$, p=1.0
P2	Increased vigilance due to feeling unsafe	0 (0.0%)	8 (3.7%)	$\chi^2(1)=0.492$, p=1.0
P2	Ideas of being threatened/observed by supernatural / invisible beings	0 (0.0%)	7 (3.2%)	$\chi^2(1)=0.428$, p=1.0
P2	Ideas of being observed anonymously (e.g. by cameras, internet etc.)	0 (0.0%)	1 (0.5%)	$\chi^2(1)=0.060$, p=1.0
P2	Ideas of being under surveillance (not solely observation)	0 (0.0%)	5 (2.3%)	$\chi^2(1)=0.303$, p=1.0
P2	Ideas of persecution	0 (0.0%)	9 (4.1%)	$\chi^2(1)=0.556$, p=1.0
P2	Ideas of being excluded	0 (0.0%)	6 (2.7%)	$\chi^2(1)=0.366$, p=1.0

SIPS No.	Content	Somati-zation disorder present (n=13)	Somati-zation disorder absent (n=219)	Statistics
P2	Ideas that others intend to harm the patient (not physically)	1 (7.7%)	28 (12.8%)	$\chi^2(1)=0.291$, p=1.0
P2	Ideas that others intend to poison the patient	0 (0.0%)	2 (0.9%)	$\chi^2(1)=0.120$, p=1.0
P2	Ideas that others intend to physically harm the patient	1 (7.7%)	24 (11.0%)	$\chi^2(1)=0.136$, p=1.0
P2	Ideas that supernatural beings intend to harm the patient	**1 (7.7%)**	0 (0.0%)	$\chi^2(1)=16.919$, p=.056
P2	Ideas of being at risk of falling victim to terror attacks or similar	0 (0.0%)	2 (0.9%)	$\chi^2(1)=0.120$, p=1.0
P3	Grandiose ideas with respect to own (natural) abilities	1 (7.7%)	9 (4.1%)	$\chi^2(1)=0.382$, p=.445
P3	Grandiose ideas of becoming famous	0 (0.0%)	5 (2.3%)	$\chi^2(1)=0.303$, p=1.0
P3	Grandiose ideas of being chosen to fulfil a greater plan (e.g. by God)	0 (0.0%)	3 (1.4%)	$\chi^2(1)=0.180$, p=1.0
P3	Grandiose ideas of becoming enlightened / a higher being	0 (0.0%)	1 (0.5%)	$\chi^2(1)=0.060$, p=1.0
P3	Grandiose ideas with respect to own supernatural abilities	0 (0.0%)	4 (1.8%)	$\chi^2(1)=0.242$, p=1.0
P3	Grandiose ideas of being a god / higher being	0 (0.0%)	1 (0.5%)	$\chi^2(1)=0.060$, p=1.0

Contents highlighted in grey have at least trend significance (p<0.100).
Numbers in **bold** signify cells with a standardized residuum ≥ |1.96|.

Supplementary Table 9b. Comparisons of the frequency of perceptual abnormalities/ hallucinations (SIPS-P4) in CHR patients with and without somatization disorder (N=232).

SIPS No.	Content	Somatization disorder present (n=13)	Somatization disorder absent (n=219)	Statistics
P4B	Acoustic illusions	0 (0.0%)	3 (1.4%)	$\chi^2(1)=0.180$, p=1.0
P4B	Hearing sounds made by non-living objects	0 (0.0%)	18 (8.2%)	$\chi^2(1)=1.158$, p=.606
P4B	Hearing sounds made by living beings (humans, animals)	0 (0.0%)	8 (3.7%)	$\chi^2(1)=0.492$, p=1.0
P4B	Audible thoughts (not by others)	0 (0.0%)	1 (0.5%)	$\chi^2(1)=0.060$, p=1.0
P4B	Hearing one's own name being called	0 (0.0%)	14 (6.4%)	$\chi^2(1)=0.884$, p=1.0
P4B	Hearing of unintelligible voices (e.g. murmur)	1 (7.7%)	12 (5.5%)	$\chi^2(1)=0.114$, p=.537
P4B	Hearing of dialoguing voices	0 (0.0%)	2 (0.9%)	$\chi^2(1)=0.120$, p=1.0
P4B	Hearing of commenting voices	0 (0.0%)	6 (2.7%)	$\chi^2(1)=0.366$, p=1.0
P4B	Hearing of imperative voices	0 (0.0%)	7 (3.2%)	$\chi^2(1)=0.428$, p=1.0
P4B	Hearing of insulting voices	0 (0.0%)	9 (4.1%)	$\chi^2(1)=0.556$, p=1.0
P4	Hearing of God's voice	0 (0.0%)	1 (0.5%)	$\chi^2(1)=0.060$, p=1.0
P4C	Sensing a presence	1 (7.7%)	13 (5.9%)	$\chi^2(1)=0.067$, p=.565
P4C	Sensing the presence of deceased persons 8	0 (0.0%)	1 (0.5%)	$\chi^2(1)=0.060$, p=1.0
P4	Seeing moving shadows in the corner of the eye	2 (15.4%)	18 (8.2%)	$\chi^2(1)=0.800$, p=.311
P4C	Visual illusions	0 (0.0%)	5 (2.3%)	$\chi^2(1)=0.303$, p=1.0
P4C	Illusions of objects moving	0 (0.0%)	1 (0.5%)	$\chi^2(1)=0.060$, p=1.0
P4C	Dysmorphophobic illusions	0 (0.0%)	2 (0.9%)	$\chi^2(1)=0.120$, p=1.0
P4C	Indistinct visual hallucinations	0 (0.0%)	5 (2.3%)	$\chi^2(1)=0.303$, p=1.0
P4C	Distinct visual hallucinations	2 (15.4%)	9 (4.1%)	$\chi^2(1)=3.454$, p=.120
P4C	Seeing a person's shape	1 (7.7%)	7 (3.2%)	$\chi^2(1)=.745$, p=.374
P4C	Confusion of persons	0 (0.0%)	1 (0.5%)	$\chi^2(1)=0.060$, p=1.0
P4D	Sense of being touched	1 (7.7%)	4 (1.8%)	$\chi^2(1)=2.002$, p=.252
P4D	Sense of changed body functions	**2 (15.4%)**	4 (1.8%)	$\chi^2(1)=8.954$, p=.038
P4D	Non-painful bodily sensation	3 (23.1%)	18 (8.2%)	$\chi^2(1)=3.291$, p=.101
P4D	Painful bodily sensation	1 (7.7%)	7 (3.2%)	$\chi^2(1)=.745$, p=.374
P4D	Sense of being infested by parasites	0 (0.0%)	1 (0.5%)	$\chi^2(1)=0.060$, p=1.0
P4D	Sensing normally non-sensible body functions (e.g. blood flow)	0 (0.0%)	1 (0.5%)	$\chi^2(1)=0.060$, p=1.0
P4E	Olfactoric hallucinations	0 (0.0%)	5 (0.5%)	$\chi^2(1)=.303$, p=1.0
P4	Gustatoric hallucinations	0 (0.0%)	1 (0.5%)	$\chi^2(1)=0.060$, p=1.0

Contents highlighted in grey have at least trend significance (p<0.100).
Numbers in **bold** signify cells with a standardized residuum ≥ |1.96|.

Supplementary Table 9c. Comparisons of the frequency of signs disorganized communication (SIPS-P5) in CHR patients with and without somatization disorder (N=232).

SIPS No.	Content	Somatization disorder present (n=13)	Somatization disorder absent (n=219)	Statistics
P5 3	Communication is vague	0 (0.0%)	2 (0.9%)	$\chi^2(1)=0.120$, p=1.0
P5	Poverty of speech	**1 (7.6%)**	2 (0.9%)	$\chi^2(1)=4.418$, p=.160
P5	Neologisms	**1 (7.6%)**	1 (0.5%)	$\chi^2(1)=7.518$, p=.109
P5	Extremely short, non-elaborative speech	0 (0.0%)	1 (0.5%)	$\chi^2(1)=0.060$, p=1.0
P5	Losing the thread of thoughts (self-experienced)	2 (15.4%)	13 (5.9%)	$\chi^2(1)=1.812$, p=.201
P5	Losing the thread of thoughts (observed by others)	2 (15.4%)	16 (7.3%)	$\chi^2(1)=1.119$, p=.266
P5	Derailment (self-experienced)	0 (0.0%)	1 (0.5%)	$\chi^2(1)=0.060$, p=1.0
P5	Derailment (observed by others)	0 (0.0%)	7 (3.2%)	$\chi^2(1)=0.428$, p=1.0
P5	Paralogia / alogia	0 (0.0%)	1 (0.5%)	$\chi^2(1)=0.060$, p=1.0
P5	Tangentiality (observed by others)	1 (7.7%)	6 (2.7%)	$\chi^2(1)=1.029$, p=.336
P5	Thought blockage by intrusion (self-experienced)	0 (0.0%)	1 (0.5%)	$\chi^2(1)=0.060$, p=1.0
P5	Thought blockage by intrusion (observed by others)	0 (0.0%)	2 (0.9%)	$\chi^2(1)=0.120$, p=1.0
P5	Thought intrusion (observed by others)	0 (0.0%)	1 (0.5%)	$\chi^2(1)=0.060$, p=1.0
P5	Circumstantial speech	0 (0.0%)	5 (2.3%)	$\chi^2(1)=.303$, p=1.0
P5	Restricted / stereotyped thinking (observed by others)	0 (0.0%)	1 (0.5%)	$\chi^2(1)=0.060$, p=1.0
P5	Stilted or pedantic speech	0 (0.0%)	2 (0.9%)	$\chi^2(1)=0.120$, p=1.0
P5	Use of inadequate words	0 (0.0%)	1 (0.5%)	$\chi^2(1)=0.060$, p=1.0

Contents highlighted in grey have at least trend significance (p<0.100).
Numbers in **bold** signify cells with a standardized residuum ≥ |1.96|.

Supplementary Table 10a. Comparisons of the frequency of attenuated and transient delusional ideas (SIPS-P1, SIPS-P2, SIPS-P3) in CHR patients with and without OCD (N=232).

SIPS No.	Content	OCD present (n=20)	OCD absent (n=212)	Statistics
P1B	Thought insertion	1 (5.0%)	9 (4.2%)	$\chi^2(1)=0.025$, p=.602
P1B	Thought withdrawal	**1 (5.0%)**	1 (0.5%)	$\chi^2(1)=4.385$, p=.165
P1B	Audible thoughts (by others)	2 (10.0%)	10 (4.7%)	$\chi^2(1)=1.040$, p=.277
P1B	Experiences of mind being read	2 (10.0%)	15 (7.1%)	$\chi^2(1)=0.203$, p=.647
P1B	Thought broadcasting	0 (0.0%)	7 (3.3%)	$\chi^2(1)=0.681$, p=1.0
P1B	Experience of being controlled by external forces	0 (0.0%)	1 (0.5%)	$\chi^2(1)=2.002$, p=.252
P1C	Ideas that strangers know something about patient	0 (0.0%)	2 (0.9%)	$\chi^2(1)=0.190$, p=1.0
P1C	Ideas that own thoughts could become real	0 (0.0%)	1 (0.5%)	$\chi^2(1)=0.095$, p=1.0
P1C	Ideas that own actions would influence the surrounding	0 (0.0%)	1 (0.5%)	$\chi^2(1)=0.095$, p=1.0
P1C	Belief in supernatural phenomena (ghosts, telepathy, afterlife, power of the universe etc.)	1 (5.0%)	7 (3.3%)	$\chi^2(1)=0.158$, p=.519
P1C	Numbers have special meaning	0 (0.0%)	3 (1.4%)	$\chi^2(1)=0.287$, p=1.0
P1C	Ideas that positive thoughts might cause bad things	0 (0.0%)	1 (0.5%)	$\chi^2(1)=0.095$, p=1.0
P1C	Ideas of being directly affected by other persons feelings/actions	0 (0.0%)	2 (0.9%)	$\chi^2(1)=0.190$, p=1.0
P1C	Belief that everything is connected	**1 (5.0%)**	0 (0.0%)	$\chi^2(1)=10.646$, p=.086
P1C	Belief in conspiracy theories	**1 (5.0%)**	0 (0.0%)	$\chi^2(1)=10.646$, p=.086
P1C	Tendency to see relations between random events	**1 (5.0%)**	1 (0.5%)	$\chi^2(1)=4.385$, p=.165
P1C	Ideas that others take over the patient's self/personality	0	1 (0.5%)	$\chi^2(1)=0.095$, p=1.0
P1D	Belief in fate	**1 (5.0%)**	0 (0.0%)	$\chi^2(1)=10.646$, p=.086
P1C	Unusual religious ideas	0 (0.0%)	1 (0.5%)	$\chi^2(1)=0.095$, p=1.0
P1C	Unusual ideas about the world	0 (0.0%)	2 (0.9%)	$\chi^2(1)=0.190$, p=1.0
P1C	Ideas that things in the surrounding have a special meaning (no ideas of reference)	0 (0.0%)	3 (1.4%)	$\chi^2(1)=0.287$, p=1.0
P1D	Unusual and unrealistic ideas about the own body	1 (5.0%)	5 (2.4%)	$\chi^2(1)=0.506$, p=.421
P1D	Hypochondriacal ideas	3 (15.0%)	11 (5.2%)	$\chi^2(1)=3.310$, p=.108
P1D	Ideas of being pregnant	0 (0.0%)	1 (0.5%)	$\chi^2(1)=0.095$, p=1.0
P1D	Nihilistic ideas about own non-existence	3 (15.0%)	14 (6.6%)	$\chi^2(1)=1.897$, p=.170
P1D	Ideas of the existence of another reality / universe	0 (0.0%)	2 (0.9%)	$\chi^2(1)=0.190$, p=1.0

SIPS No.	Content	OCD present (n=20)	OCD absent (n=212)	Statistics
P1D	Nihilistic ideas about the non-existence of others	3 (15.0%)	22 (10.4%)	$\chi^2(1)=0.406$, p=.460
P1D	Ideas of vanishing from the world	0 (0.0%)	2 (0.9%)	$\chi^2(1)=0.190$, p=1.0
P1D	Ideas of being part of a movie, computer game etc.	0 (0.0%)	3 (1.4%)	$\chi^2(1)=0.287$, p=1.0
P1D	Ideas that a part of the soul is separated	0 (0.0%)	1 (0.5%)	$\chi^2(1)=0.095$, p=1.0
P1D	Ideas of not being a human being	**1 (5.0%)**	0 (0.0%)	$\chi^2(1)=10.646$, p=.086
P1D	Identity confusion (patient thinks s/he is someone else)	0 (0.0%)	1 (0.5%)	$\chi^2(1)=0.095$, p=1.0
P1D	Nihilistic ideas of being dead / dying	**1 (5.0%)**	1 (0.5%)	$\chi^2(1)=4.385$, p=.165
P1D	Demarcation experiences	0 (0.0%)	1 (0.5%)	$\chi^2(1)=0.095$, p=1.0
P1D	Ideas of observing oneself from a birds-eye perspective	**1 (5.0%)**	1 (0.5%)	$\chi^2(1)=4.385$, p=.165
P1D	Exaggerated ideas of guilt	2 (10.0%)	13 (6.1%)	$\chi^2(1)=0.452$, p=.625
P1D	Ideas of jealousy	0 (0.0%)	4 (1.9%)	$\chi^2(1)=0.384$, p=1.0
P1D	Erotomanic ideas	0 (0.0%)	2 (0.9%)	$\chi^2(1)=0.190$, p=1.0
P1E	Ideas of being the center of non-negative attention	3 (15.0%)	23 (10.8%)	$\chi^2(1)=0.316$, p=.477
P1E	Non-paranoid ideas of being especially addressed by random events (e.g. media)	1 (5.0%)	23 (10.8%)	$\chi^2(1)=0.674$, p=.703
P2	General mistrust	1 (5.0%)	13 (6.1%)	$\chi^2(1)=0.041$, p=1.0
P2	Mistrust of friends	0 (0.0%)	8 (3.8%)	$\chi^2(1)=0.782$, p=1.0
P2	Paranoid ideas of reference (gazes of passers-by)	2 (10.0%)	55 (25.9%)	$\chi^2(1)=2.507$, p=.172
P2	Paranoid ideas of reference involving friends / family	0 (0.0%)	10 (4.7%)	$\chi^2(1)=0.986$, p=1.0
P2	Ideas that others wish the patient ill	0 (0.0%)	1 (0.5%)	$\chi^2(1)=0.095$, p=1.0
P2	Ideas that others would exploit the patient	0 (0.0%)	4 (1.9%)	$\chi^2(1)=0.384$, p=1.0
P2	Increased vigilance due to feeling unsafe	0 (0.0%)	8 (3.8%)	$\chi^2(1)=0.782$, p=1.0
P2	Ideas of being threatened/observed by supernatural / invisible beings	0 (0.0%)	7 (3.3%)	$\chi^2(1)=0.681$, p=1.0
P2	Ideas of being observed anonymously (e.g., by cameras, internet etc.)	**1 (5.0%)**	1 (0.5%)	$\chi^2(1)=10.646$, p=.086
P2	Ideas of being under surveillance (not solely observation)	1 (5.0%)	4 (1.9%)	$\chi^2(1)=0.840$, p=.365
P2	Ideas of persecution	2 (10.0%)	7 (3.3%)	$\chi^2(1)=2.199$, p=.176
P2	Ideas of being excluded	0 (0.0%)	6 (2.8%)	$\chi^2(1)=0.581$, p=1.0

SIPS No.	Content	OCD present (n=20)	OCD absent (n=212)	Statistics
P2	Ideas that others intend to harm the patient (not physically)	3 (15.0%)	26 (12.3%)	$\chi^2(1)=0.125$, p=.723
P2	Ideas that others intend to poison the patient	0 (0.0%)	2 (0.9%)	$\chi^2(1)=0.190$, p=1.0
P2	Ideas that others intend to physically harm the patient	2 (10.0%)	23 (10.8%)	$\chi^2(1)=0.014$, p=1.0
P2	Ideas that supernatural beings intend to harm the patient	0 (0.0%)	1 (0.5%)	$\chi^2(1)=0.095$, p=1.0
P2	Ideas of being at risk of falling victim to terror attacks or similar	0 (0.0%)	2 (0.9%)	$\chi^2(1)=0.190$, p=1.0
P3	Grandiose ideas with respect to own (natural) abilities	1 (5.0%)	9 (4.2%)	$\chi^2(1)=0.025$, p=.602
P3	Grandiose ideas of becoming famous	**2 (10.0%)**	3 (1.4%)	$\chi^2(1)=6.387$, p=.060
P3	Grandiose ideas of being chosen to fulfil a greater plan (e.g., by God)	0 (0.0%)	3 (1.4%)	$\chi^2(1)=0.287$, p=1.0
P3	Grandiose ideas of becoming enlightened / a higher being	0 (0.0%)	1 (0.5%)	$\chi^2(1)=0.095$, p=1.0
P3	Grandiose ideas with respect to own supernatural abilities	1 (5.0%)	3 (1.4%)	$\chi^2(1)=1.386$, p=.304
P3	Grandiose ideas of being a god / higher being	0 (0.0%)	1 (0.5%)	$\chi^2(1)=0.095$, p=1.0

Contents highlighted in grey have at least trend significance (p<0.100).
Numbers in **bold** signify cells with a standardized residuum ≥ |1.96|.

Supplementary Table 10b. Comparisons of the frequency of perceptual abnormalities/hallucinations (SIPS-P4) in CHR patients with and without OCD (N=232).

SIPS No.	Content	OCD present (n=13)	OCD absent (n=219)	Statistics
P4B	Acoustic illusions	0 (0.0%)	3 (1.4%)	$\chi^2(1)$=0.287, p=1.0
P4B	Hearing sounds made by non-living objects	2 (10.0%)	16 (7.5%)	$\chi^2(1)$=0.154, p=.659
P4B	Hearing sounds made by living beings (humans, animals)	0 (0.0%)	8 (3.8%)	$\chi^2(1)$=0.782, p=1.0
P4B	Audible thoughts (not by others)	0 (0.0%)	1 (0.5%)	$\chi^2(1)$=0.095, p=1.0
P4B	Hearing one's own name being called	2 (10.0%)	12 (5.7)	$\chi^2(1)$=0.607, p=.344
P4B	Hearing of unintelligible voices (e.g. murmur)	1 (5.0%)	12 (5.7%)	$\chi^2(1)$=0.015, p=1.0
P4B	Hearing of dialoguing voices	0 (0.0%)	2 (0.9%)	$\chi^2(1)$=0.190, p=1.0
P4B	Hearing of commenting voices	0 (0.0%)	6 (2.8%)	$\chi^2(1)$=0.581, p=1.0
P4B	Hearing of imperative voices	0 (0.0%)	7 (3.3%)	$\chi^2(1)$=0.681, p=1.0
P4B	Hearing of insulting voices	1 (5.0%)	8 (3.8%)	$\chi^2(1)$=0.074, p=.562
P4	Hearing of God's voice	0 (0.0%)	1 (0.5%)	$\chi^2(1)$=0.095, p=1.0
P4C	Sensing a presence	1 (5.0%)	13 (6.1%)	$\chi^2(1)$=0.041, p=1.0
P4C	Sensing the presence of deceased persons	0 (0.0%)	1 (0.5%)	$\chi^2(1)$=0.095, p=1.0
P4	Seeing moving shadows in the corner of the eye	2 (10.0%)	18 (8.5%)	$\chi^2(1)$=0.053, p=.685
P4C	Visual illusions	**2 (10.0%)**	3 (1.4%)	$\chi^2(1)$=6.387, p=.060
P4C	Illusions of objects moving	0 (0.0%)	1 (0.5%)	$\chi^2(1)$=0.095, p=1.0
P4C	Dysmorphophobic illusions	0 (0.0%)	2 (0.9%)	$\chi^2(1)$=0.190, p=1.0
P4C	Indistinct visual hallucinations	0 (0.0%)	5 (2.4%)	$\chi^2(1)$=0.482, p=1.0
P4C	Distinct visual hallucinations	1 (5.0%)	10 (4.7%)	$\chi^2(1)$=0.003, p=1.0
P4C	Seeing a person's shape	0 (5.0%)	8 (3.8%)	$\chi^2(1)$=0.782 p=1.0
P4C	Confusion of persons	0 (0.0%)	1 (0.5%)	$\chi^2(1)$=0.095, p=1.0
P4D	Sense of being touched	0 (0.0%)	5 (2.4%)	$\chi^2(1)$=0.482, p=1.0
P4D	Sense of changed body functions	1 (5.0%)	5 (2.4%)	$\chi^2(1)$=0.506, p=.421
P4D	Non-painful bodily sensation	3 (15.0%)	18 (8.5%)	$\chi^2(1)$=0.941, p=.403
P4D	Painful bodily sensation	2 (10.0%)	6 (2.8%)	$\chi^2(1)$=2.822, p=.144
P4D	Sense of being infested by parasites	0 (0.0%)	1 (0.5%)	$\chi^2(1)$=0.095, p=1.0
P4D	Sensing normally non-sensible body functions (e.g., blood flow)	0 (0.0%)	1 (0.5%)	$\chi^2(1)$=0.095, p=1.0
P4E	Olfactoric hallucinations	1 (5.0%)	4 (1.9%)	$\chi^2(1)$=0.849, p=.365
P4	Gustatoric hallucinations	0 (0.0%)	1 (0.5%)	$\chi^2(1)$=0.095, p=1.0

Contents highlighted in grey have at least trend significance ($p<0.100$).
Numbers in **bold** signify cells with a standardized residuum ≥ |1.96|.

Supplementary Table 10c. Comparisons of the frequency of signs of disorganized communication (SIPS-P5) in CHR patients with and without OCD (N=232).

SIPS No.	Content	OCD present (n=13)	OCD absent (n=219)	Statistics
P5	Communication is vague	0 (0.0%)	2 (0.9%)	$\chi^2(1)=0.190$, $p=1.0$
P5	Poverty of speech	1 (5.0%)	2 (0.9%)	$\chi^2(1)=2.356$, $p=.238$
P5	Neologisms	0 (0.0%)	2 (0.9%)	$\chi^2(1)=0.190$, $p=1.0$
P5	Extremely short, non-elaborative speech	0 (0.0%)	1 (0.5%)	$\chi^2(1)=0.095$, $p=1.0$
P5	Losing the thread of thoughts (self-experienced)	6 (30.0%)	9 (4.2%)	$x^2(1)=20.045$, $p<.001$
P5	Losing the thread of thoughts (observed by others)	**6 (30.0%)**	12 (5.7%)	$\chi^2(1)=15.128$, $p=.002$
P5	Derailment (self-experienced)	0 (0.0%)	1 (0.5%)	$\chi^2(1)=0.095$, $p=1.0$
P5	Derailment (observed by others)	0 (0.0%)	7 (3.3%)	$\chi^2(1)=0.681$, $p=1.0$
P5	Paralogia / alogia	0 (0.0%)	1 (0.5%)	$\chi^2(1)=0.095$, $p=1.0$
P5	Tangentiality (observed by others)	0 (0.0%)	7 (3.3%)	$\chi^2(1)=0.681$, $p=1.0$
P5	Thought blockage by intrusion (self-experienced)	0 (0.0%)	1 (0.5%)	$\chi^2(1)=0.095$, $p=1.0$
P5	Thought blockage by intrusion (observed by others)	0 (0.0%)	2 (0.9%)	$\chi^2(1)=0.190$, $p=1.0$
P5	Thought intrusion (observed by others)	0 (0.0%)	1 (0.5%)	$\chi^2(1)=0.095$, $p=1.0$
P5	Circumstantial speech	0 (0.0%)	5 (2.4%)	$\chi^2(1)=0.482$, $p=1.0$
P5	Restricted / stereotyped thinking (observed by others)	0 (0.0%)	1 (0.5%)	$\chi^2(1)=0.095$, $p=1.0$
P5	Stilted or pedantic speech	0 (0.0%)	2 (0.9%)	$\chi^2(1)=0.190$, $p=1.0$
P5	Use of inadequate words	0 (0.0%)	1 (0.5%)	$\chi^2(1)=0.095$, $p=1.0$

Contents highlighted in grey have at least trend significance ($p<0.100$).
Numbers in **bold** signify cells with a standardized residuum $\geq |1.96|$.

Supplementary Table 11a. Comparisons of age of CHR patients with and without the respective attenuated and transient delusional idea (SIPS-P1, SIPS-P2, SIPS-P3; N=232).

SIPS No.	Content	With idea (mean±SD; (median))	Without idea (mean±SD; (median))	Statistics
P1B	Thought insertion	24.20 ± 7.376 (21.0)	23.20 ± 5.202 (22.0)	U=1101.5, p=.967 r=-0.003
P1B	Thought withdrawal	23.50 ± 6.364 (21.0)	23.24 ± 5.303 (22.0)	U=224.0, p=.949 r=-0.004
P1B	Audible thoughts (by others)	20.42 ± 4.641 (19.0)	23.40 ± 5.296 (22.0)	U=766.5 p=.014 r=-0.161
P1B	Experiences of mind being read	20.41 ± 2.671 (20.0)	23.47 ± 5.391 (22.0)	U=1221.0, p=.022 r=-0.150
P1B	Thought broadcasting	22.29 ± 6.473 (19.0)	23.27 ± 5.271 (22.0)	U=603.0 p=.290 r=-0.070
P1B	Experience of being controlled by external forces	20.80 ± 1.643 (20.0)	23.30 ± 5.338 (22.0)	U=447.5, p=.417 r=-0.053
P1D	Ideas that strangers know something about patient	19.50 ± 0.707 (19.5)	23.27 ± 5.308 (22.0)	U=122.0, p=.251 r=-0.075
P1C	Ideas that own thoughts could become real	20.0 ± 0.000 (20.0)	23.26 ± 5.304 (22.0)	U=77.0 P=.672 r=-0.038
P1C	Ideas that own actions would influence the surrounding	22.0 ± 0.000 (22.0)	23.25 ± 5.307 (22.0)	U=110.5 p=.957 r=-0.005
P1C	Belief in supernatural phenomena (ghosts, telepathy, afterlife, etc.)	25.38 ± 6.948 (23.5)	23.17 ± 5.232 (22.0)	U=727.5, p=.365 r=-0.060
P1C	Numbers have special meaning	21.67 ± 5.686 (20.0)	23.26 ± 5.302 (22.0)	U=277.0, p=.563 r=-0.038
P1C	Ideas that positive thoughts might cause bad things	18.0 ± 0.000 (18.0)	23.26 ± 5.297 (22.0)	U=22.0, p=.198 r=-0.092
P1C	Ideas of being directly affected by other persons feelings/actions	18.00 ± 4.243 (18.0)	23.29 ± 5.289 (22.0)	U=101.5, p=.172 r=-0.090
P1C	Belief that everything is connected	21.00 ± 0.000 (21.0)	23.25 ± 5.306 (22.0)	U=101.0, p=.879 r=-0.014
P1C	Belief in conspiracy theories	28.00 ± 0.000 (28.0)	23.22 ± 5.299 (22.0)	U=40.0, p=.353 r=-0.074
P1C	Tendency to see relations between random events	23.50 ± 2.121 (23.5)	23.24 ± 5.318 (22.0)	U=178.5, p=.584 r=-0.036
P1C	Ideas that others take over the patient's self/personality	34.00 ± 0.000 (34.0)	23.19 ± 5.260 (22.0)	U=10.5, p=.095 r=-0.103
P1D	Belief in fate	21.00 ± 0.000 (21.0)	23.25 ± 5.306 (22.0)	U=101.0, p=.879 r=-0.014
P1C	Unusual religious ideas	20.0 ± 0.000 (20.0)	23.26 ± 5.304 (22.0)	U=77.0, p=.672 r=-0.038
P1C	Unusual ideas about the world	25.50 ± 6.364 (25.5)	23.22 ± 5.299 (22.0)	U=160.0, p=.457 r=-0.049
P1C	Ideas that things in the surrounding have a special meaning (no ideas of reference)	21.00 ± 2.000 (21.0)	23.27 ± 5.322 (22.0)	U=281.0, p=.587 r=-0.036

SIPS No.	Content	With idea (mean±SD; (median))	Without idea (mean±SD; (median))	Statistics
P1D	Unusual and unrealistic ideas about the own body	24.20 ± 1.633 (21.33)	23.29 ± 5.352 (22.0)	U=603.5, p=.645 r=-0.303
P1D	Hypochondriacal ideas	25.07 ± 7.195 (24.50)	23.12 ± 5.151 (22.0)	U=1292.0, p=.335 r=-0.063
P1D	Ideas of being pregnant	19.0 ± 0.000 (19.0)	23.26 ± 5.301 (22.0)	U=46.0, p=.405 r=-0.068
P1D	Nihilistic ideas about own non-existence	22.18 ± 5.247 (20.0)	23.33 ± 5.304 (22.0)	U=1569.6 p=.331 r=-0.064
P1D	Ideas of the existence of another reality / universe	20.50 ± 0.707 (20.5)	23.27 ± 5.313 (22.0)	U=177.0 p=.574 r=-0.037
P1D	Nihilistic ideas about the non-existence of others	20.88 ± 4.076 (20.0)	23.54 ± 5.363 (22.0)	U=1799.0, p=.013 r=-0.164
P1D	Ideas of vanishing from the world	21.0 ± 2.828 (21.0)	23.26 ± 5.312 (22.0)	U=182.0, p=.610 r=-0.033
P1D	Ideas of being part of a movie, computer game etc.	20.67 ± 3.512 (21.0)	23.28 ± 5.313 (22.0)	U=261.5, p=.476 r=-0.049
P1D	Ideas that a part of the soul is separated	20.0 ± 0.000 (20.0)	23.26 ± 5.304 (22.0)	U=77.0, p=.672 r=-0.038
P1D	Ideas of not being a human being	21.0 ± 0.000 (21.0)	23.25 ± 5.306 (22.0)	U=101.0, p=.879 r=-0.014
P1D	Identity confusion (patient thinks s/he is someone else)	34.0 ± 0.000 (34.0)	23.19 ± 5.260 (22.0)	U=10.5, p=.095 r=-0.103
P1D	Nihilistic ideas of being dead / dying	20.0 ± 0.000 (20.0)	23.27 ± 5.311 (22.0)	U=1530, p=.414 r=-0.054
P1D	Demarcation experiences	15.0 ± 0.000 (15.0)	23.28 ± 5.280 (22.0)	U=1.5, p=.017 r=-0.112
P1D	Ideas of observing oneself from a birds-eye perspective	23.50 ± 4.950 (23.5)	23.24 ± 5.310 (22.0)	U=203.5, p=.778 r=-0.018
P1D	Exaggerated ideas of guilt	21.67 ± 3.619 (22.0)	23.35 ± 5.382 (22.0)	U=1448.0, p=.474 r=-0.047
P1D	Ideas of jealousy	28.25 ± 7.411 (26.5)	23.15 ± 5.232 (22.0)	U=233.0, p=.093 r=-0.110
P1D	Erotomanic ideas	22.50 ± 0.707 (22.5)	23.25 ± 5.319 (22.0)	U=203.5, p=.778 r=-0.018
P1E	Ideas of being the center of non-negative attention	22.38 ± 6.670 (19.5)	23.35 ± 5.107 (22.0)	U=2127.0, p=.086 r=-0.113
P1E	Non-paranoid ideas of being especially addressed by random events (e.g., media)	23.54 ± 5.687 (22.0)	23.21 ± 5.263 (22.0)	U=2383.0, p=.716 r=-0.024
P2	General mistrust	22.93 ± 4.428 (22.5)	23.26 ± 5.356 (22.0)	U=1504.0, p=.928 r=-0.006
P2	Mistrust of friends	20.38 ± 3.739 (20.5)	23.34 ± 5.321 (22.0)	U=645.5, p=.178 r=-0.089

SIPS No.	Content	With idea (mean±SD; (median))	Without idea (mean±SD; (median))	Statistics
P2	Paranoid ideas of reference (gazes of passers-by)	21.65 ± 4.760 (21.0)	23.76 ± 5.371 (22.0)	U=3782.5, p=.006 r=-0.180
P2	Paranoid ideas of reference involving friends / family	24.00 ± 6.799 (22.0)	23.21 ± 5.236 (22.0)	U=1046.0, p=.757 r=-0.020
P2	Ideas that others wish the patient ill	22.0 ± 0.000 (22.0)	23.25 ± 5.307 (22.0)	U=110.5, p=.957 r=-0.005
P2	Ideas that others would exploit the patient	28.0 ± 7.572 (25.0)	23.16 ± 5.233 (22.0)	U=234.0, p=.094 r=-0.110
P2	Increased vigilance due to feeling unsafe	24.0 ± 7.010 (21.5)	23.21 ± 5.244 (22.0)	U=879.5, p=.929 r=-0.006
P2	Ideas of being threatened/observed by supernatural / invisible beings	21.0 ± 4.655 (20.0)	23.31 ± 5.309 (22.0)	U=595.0, p=.270 r=-0.072
P2	Ideas of being observed anonymously (e.g., by cameras, internet etc.)	22.0 ± 0.000 (22.0)	23.25 ± 5.307 (22.0)	U=110.5, p=.957 r=-0.005
P2	Ideas of being under surveillance (not solely observation)	27.0 ± 8.062 (26.0)	23.16 ± 5.216 (22.0)	U=403.0, p=.266 r=-0.073
P2	Ideas of persecution	23.33 ± 5.657 (23.0)	23.24 ± 5.295 (22.0)	U=956.5, p=.811 r=-0.016
P2	Ideas of being excluded	22.67 ± 2.338 (22.0)	23.26 ± 5.355 (22.0)	U=615.0, p=.697 r=-0.026
P2	Ideas that others intend to harm the patient (not physically)	24.76 ± 5.944 (23.0)	23.02 ± 5.177 (21.0)	U=2331.5, p=.069 r=-0.119
P2	Ideas that others intend to poison the patient	25.0 ± 2.828 (25.0)	23.23 ± 5.314 (22.0)	U=143.5, p=.358 r=-0.060
P2	Ideas that others intend to physically harm the patient	22.88 ± 5.681 (21.0)	23.29 ± 5.261 (22.0)	U=2411.0, p=.576 r=-0.037
P2	Ideas that supernatural beings intend to harm the patient	19.0 ± 0.000 (19.0)	23.26 ± 5.301 (22.0)	U=46.0, p=.405 r=-0.068
P2	Ideas of being at risk of falling victim to terror attacks or similar	29.50 ± 0.707 (29.5)	23.19 ± 5.287 (22.0)	U=64.5, p=.079 r=-0.115
P3	Grandiose ideas with respect to own (natural) abilities	24.30 ± 6.848 (23.5)	23.19 ± 5.231 (22.0)	U=1020.5, p=.665 r=-0.028
P3	Grandiose ideas of becoming famous	24.20 ± 3.633 (23.0)	23.22 ± 5.331 (22.0)	U=428.5, p=.347 r=-0.062
P3	Grandiose ideas of being chosen to fulfil a greater plan (e.g., by God)	19.67 ± 0.577 (20.0)	23.29 ± 5.315 (22.0)	U=197.0, p=.203 r=-0.084
P3	Grandiose ideas of becoming enlightened / a higher being	24.0 ± 0.000 (24.0)	23.24 ± 5.308 (22.0)	U=79.5, p=.690 r=-0.035
P3	Grandiose ideas with respect to own supernatural abilities	26.50 ± 7.141 (28.5)	23.18 ± 5.262 (22.0)	U=321.0, p=.309 r=-0.067
P3	Grandiose ideas of being a god / higher being	17.00 ± 0.000 (17.0)	23.27 ± 5.292 (22.0)	U=12.0, p=.112 r=-0.102

Contents highlighted in grey have at least trend significance (p<0.100).

Supplementary Table 11b. Comparisons of age of CHR patients with and without the respective perceptual abnormalities/hallucinations (SIPS-P4; N=232).

SIPS No.	Content	With idea (mean±SD; (median))	Without idea (mean±SD; (median))	Statistics
P4B	Acoustic illusions	30.67 ± 4.163 (32.0)	23.14 ± 5.247 (22.0)	U=88.0, p=.026 r=-0.146
P4B	Hearing sounds made by non-living objects	22.11 ± 4.689 (21.0)	23.34 ± 5.343 (22.0)	U=1667.0, p=.342 r=-0.062
P4B	Hearing sounds made by living beings (humans, animals)	23.25 ± 6.182 (22.0)	23.24 ± 5.278 (22.0)	U=873.5, p=.904 r=-0.008
P4B	Audible thoughts (not by others)	18.00 ± 0.000 (18.0)	23.26 ± 5.297 (22.0)	U=22.0, p=.161 r=-0.092
P4B	Hearing one's own name being called	20.93 ± 4.763 (19.0)	23.39 ± 5.304 (22.0)	U=1057.0, p=.053 r=-0.127
P4B	Hearing of unintelligible voices (e.g. murmur)	22.69 ± 5.202 (22.0)	23.27 ± 5.312 (22.0)	U=1345.0, p=.739 r=-0.022
P4B	Hearing of dialoguing voices	27.00 ± 12.728 (27.0)	23.21 ± 5.241 (22.0)	U=214.0, p=.865 r=-0.011
P4B	Hearing of commenting voices	21.33 ± 2.582 (21.5)	23.29 ± 5.344 (22.0)	U=573.0, p=.518 r=-0.042
P4B	Hearing of imperative voices	22.00 ± 5.538 (20.0)	23.14 ± 5.247 (22.0)	U=628.5, p=.361 r=-0.060
P4B	Hearing of insulting voices	23.22 ± 6.888 (21.0)	23.24 ± 5.242 (22.0)	U=901.5, p=.604 r=-0.034
P4	Hearing of God's voice	16.00 ± 0.000 (16.0)	23.27 ± 5.286 (22.0)	U=6.0, p=.101 r=-0.108
P4C	Sensing a presence	20.93 ± 4.698 (20.0)	23.39 ± 5.308 (22.0)	U=1067.5, p=.059 r=-0.124
P4C	Sensing the presence of deceased persons	27.00 ± 0.000 (27.0)	23.23 ± 5.302 (22.0)	U=50.5, p=.440 r=-0.064
P4	Seeing moving shadows in the corner of the eye	21.20 ± 3.792 (20.5)	23.43 ± 5.384 (22.0)	U=1636.5, p=.091, r=-0.111
P4C	Visual illusions	22.20 ± 6.723 (20.0)	23.26 ± 5.277 (22.0)	U=432.5, p=.361 r=-0.060
P4C	Illusions of objects moving	25.00 ± 0.000 (25.0)	23.23 ± 5.307 (22.0)	U=69.0, p=.486 r=-0.046
P4C	Dysmorphophobic illusions	23.00 ± 2.82 (23.0)	23.24 ± 5.316 (22.0)	U=198.0, p=.734 R=-0.022
P4C	Indistinct visual hallucinations	19.60 ± .548 (20.0)	23.32 ± 5.326 (22.0)	U=313.0, p=.085 r=-0.113
P4C	Distinct visual hallucinations	22.18 ± 4.020 (21.0)	23.29 ± 5.345 (22.0)	U=1108.0, p=.619 r=-0.033
P4C	Seeing a person's shape	21.88 ± 4.224 (21.0)	23.29 ± 5.332 (22.0)	U=776.0, p=.518 r=-0.042
P4C	Confusion of persons	30.00 ± 0.000 (30.0)	23.10 ± 5.289 (22.0)	U=31.0, p=.276 r=-0.083

SIPS No.	Content	With idea (mean±SD; (median))	Without idea (mean±SD; (median))	Statistics
P4D	Sense of being touched	18.80 ± 1.304 (19.0)	23.24 ± 5.310 (22.0)	U=224.0, p=.020 r=-0.152
P4D	Sense of changed body functions	25.50 ± 6.979 (24.0)	23.18 ± 5.252 (22.0)	U=515.0, p=.313 r=-0.066
P4D	Non-painful bodily sensation	23.62 ± 4.674 (24.0)	23.20 ± 5.363 (22.0)	U=1959.5, p=.381 r=-0.058
P4D	Painful bodily sensation	20.87 ± 3.603 (20.5)	23.33 ± 5.333 (22.0)	U=668.5, p=.221 r=-0.080
P4D	Sense of being infested by parasites	21.00 ± 0.000 (21.0)	23.25 ± 5.306 (22.0)	U=101.0, p=.879 r=-0.014
P4D	Sensing normally non-sensible body functions (e.g., blood flow)	24.00 ± 0.000 (24.0)	23.24 ± 5.308 (22.0)	U=79.5, p=.590 r=-0.035
P4E	Olfactory hallucinations	20.40 ± 1.673 (20.0)	23.30 ± 5.333 (22.0)	U=397.0, p=.249 r=-0.076
P4	Gustatory hallucinations	21.00 ± 0.000 (21.0)	23.25 ± 5.306 (22.0)	U=101.0, p=.828 r=-0.014

Contents highlighted in grey have at least trend significance (p<0.100).

Supplementary Table 11c. Comparisons of age of CHR patients with and without the respective sign of disorganized communication (SIPS-P5; N=232).

SIPS No.	Content	With idea (mean±SD; (median))	Without idea (mean±SD; (median))	Statistics
P5 3	Communication is vague	21.00 ± 1.414 (21.0)	23.26 ± 5.315 (22.0)	U=196.5, p=.722 r=-0.023
P5	Poverty of speech	32.00 ± 11.269 (38.0)	23.13 ± 5.127 (22.0)	U=187.0, p=.174 r=-0.089
P5	Neologisms	28.00 ± 14.142 (28.0)	23.20 ± 5.218 (22.0)	U=211.5, p=.844 r=-0.129
P5	Extremely short, non-elaborative speech	39.00 ± 0.000 (39.0)	23.17 ± 5.205 (22.0)	U=1.5, p=.088 r=-0.112
P5	Losing the thread of thoughts (self-experienced)	24.20 ± 5.157 (21.0)	23.18 ± 5.157 (22.0)	U=1587.0, p=.872 r=-0.011
P5	Losing the thread of thoughts (observed by others)	25.22 ± 7.765 (22.0)	23.07 ± 5.025 (22.0)	U=1690.5, p=.387 r=-0.057
P5	Derailment (self-experienced)	23.00 ± 0.000 (23.0)	23.24 ± 5.308 (22.0)	U=94.0, p=.747 r=-0.021
P5	Derailment (observed by others)	25.43 ± 6.680 (22.0)	23.17 ± 5.252 (22.0)	U=606.0, p=.298 r=-0.069
P5	Paralogia / alogia	26.00 ± 0.000 (26.0)	23.23 ± 5.305 (22.0)	U=61.0, p=.414 r=-0.068
P5	Tangentiality (observed by others)	24.14 ± 7.515 (23.0)	23.21 ± 5.234 (22.0)	U=760.5, p=.877 r=-0.010
P5	Thought blockage by intrusion (self-experienced)	19.00 ± 0.000 (19.0)	23.26 ± 5.301 (22.0)	U=46.0, p=.298 r=-0.068
P5	Thought blockage by intrusion (observed by others)	24.50 ± 7.778 (24.5)	23.23 ± 5.293 (22.0)	U=215.0, p=.873 r=-0.010
P5	Thought intrusion (observed by others)	19.00 ± 0.000 (19.0)	23.26 ± 5.301 (22.0)	U=46.0, p=.298 r=-0.020
P5	Circumstantial speech	23.80 ± 6.458 (22.0)	23.23 ± 5.285 (22.0)	U=541.0, p=.858 r=-0.012
P5	Restricted / stereotyped thinking (observed by others)	22.00 ± 0.000 (22.0)	23.25 ± 5.307 (22.0)	U=110.5, p=.940 r=-0.005
P5	Stilted or pedantic speech	19.50 ± .707 (19.5)	23.27 ± 5.308 (22.0)	U=122.0, p=.251 r=-0.075
P5	Use of inadequate words	22.00 ± 0.000 (22.0)	23.25 ± 5.307 (22.0)	U=110.5, p=.940 r=-0.005

Contents highlighted in grey have at least trend significance (p<0.100).

Supplementary Table 12a. Comparisons of social functioning (GF:S) of CHR patients with and without the respective attenuated and transient delusional idea (SIPS-P1, SIPS-P2, SIPS-P3; N=232).

SIPS No.	Content	With idea (mean±SD; (median))	Without idea (mean±SD; (median))	Statistics
P1B	Thought insertion	5.00 ± 2.108 (6.0)	6.36 ± 1.397 (6.0)	U=689.0, p=.037 r=-0.137
P1B	Thought withdrawal	4.50 ± 2.121 (4.5)	6.32 ± 1.445 (6.0)	U=94.5, p=.141 r=-0.097
P1B	Audible thoughts (by others)	6.33 ± 1.073 (6.0)	6.3 ± 1.475 (6.0)	U=1260.0, p=.785 r=-0.018
P1B	Experiences of mind being read	6.53 ± 1.505 (7.0)	6.28 ± 1.453 (6.0)	U=1611.0, p=.404 r=-0.055
P1B	Thought broadcasting	6.71 ± 1.113 (7.0)	6.29 ± 1.464 (6.0)	U=672.0, p=.497 r=-0.045
P1B	Experience of being controlled by external forces	6.20 ± 1.483 (6.0)	6.30 ± 1.457 (6.0)	U=538.5, p=.841 r=-0.013
P1E	Ideas that strangers know something about patient	4.50 ± 0.707 (4.5)	6.32 ± 1.451 (6.0)	U=55.0, p=.058 r=-0.125
P1C	Ideas that own thoughts could become real	6.00 ± 0.000 (6.0)	6.30 ± 1.458 (6.0)	U=87.0, p=.662 r=-0.029
P1C	Ideas that own actions would influence the surrounding	8.00 ± 0.000 (8.0)	6.29 ± 1.454 (6.0)	U=27.0, p=.174 r=-0.011
P1C	Belief in supernatural phenomena (ghosts, telepathy, afterlife, power of the universe etc.)	6.25 ± 1.282 (6.0)	6.30 ± 1.463 (6.0)	U=82.0, p=.675 r=-0.028
P1C	Numbers have special meaning	4.67 ± 1.455 (5.0)	6.32 ± 1.445 (6.0)	U=130.5, p=.058 r=-0.124
P1C	Ideas that positive thoughts might cause bad things	8.00 ± 0.000 (8.0)	6.29± 1.454 (6.0)	U=27.0, p=.174 r=-0.089
P1C	Ideas of being directly affected by other persons feelings/actions	8.00 ± 0.000 (8.0)	6.29 ± 1.452 (6.0)	U=53.0, p=.054 r=-0.126
P1C	Belief that everything is connected	7.00 ± 0.000 (7.0)	6.30 ± 1.457 (6.0)	U=79.0, p=.575 r=-0.037
P1C	Belief in conspiracy theories	5.00 ± 0.000 (5.0)	6.31 ± 1.455 (6.0)	U=38.0, p=.234 r=-0.078
P1C	Tendency to see relations between random events	4.50 ± 3.536 (4.5)	6.32 ± 1.432 (6.0)	U=152.5, p=.399 r=-0.055
P1C	Ideas that others take over the patient's self/personality	8.00 ± 0.000 (8.0)	6.29 ± 1.454 (6.0)	U=27.0, p=.174 r=-0.089
P1D	Belief in fate	8.00 ± 0.000 (8.0)	6.29 ± 1.454 (6.0)	U=27.0, p=.174 r=-0.011
P1C	Unusual religious ideas	8.00 ± 0.000 (8.0)	6.29 ± 1.454 (6.0)	U=27.0, p=.174 r=-0.089
P1C	Unusual ideas about the world	7.50 ± 0.707 (7.5)	6.28 ± 1.456 (6.0)	U=105.0, p=.174 r=-0.089
P1C	Ideas that things in the surrounding have a special meaning (no ideas of reference)	6.33 ± 0.577 (6.0)	6.30 ± 1.463 (6.0)	U=323.0, p=.855 r=-0.012

SIPS No.	Content	With idea (mean±SD; (median))	Without idea (mean±SD; (median))	Statistics
P1D	Unusual and unrealistic ideas about the own body	6.00 ± 2.280 (6.5)	6.31 ± 1.433 (6.0)	U=671.5, p=.967 r=-0.003
P1D	Hypochondriacal ideas	6.50 ± 1.401 (6.0)	6.29 ± 1.460 (6.0)	U=1469.0, p=.810 r=-0.016
P1D	Ideas of being pregnant	8.00 ± 0.000 (8.0)	6.29 ± 1.454 (6.0)	U=27.0, p=.174 r=-0.011
P1D	Nihilistic ideas about own non-existence	6.47 ± 1.179 (6.0)	6.29 ± 1.476 (6.0)	U=1750.5, p=.766 r=-0.019
P1D	Ideas of the existence of another reality / universe	6.00 ± 1.414 (6.0)	6.30 ± 1.458 (6.0)	U=189.0, p=.656 r=-0.030
P1D	Nihilistic ideas about the non-existence of others	6.48 ± 1.005 (7.0)	6.28 ± 1.500 (6.0)	U=2449.5, p=.655 r=-0.030
P1D	Ideas of vanishing from the world	6.50 ± 2.121 (6.5)	6.30 ± 1.454 (6.0)	U=219.0, p=.095 r=-0.008
P1D	Ideas of being part of a movie, computer game etc.	4.67 ± 2.309 (6.0)	6.32 ± 1.436 (6.0)	U=172.5, p=.128 r=-0.100
P1D	Ideas that a part of the soul is separated	8.00 ± 0.000 (8.0)	6.29 ± 1.454 (6.0)	U=27.0, p=.174 r=-0.011
P1D	Ideas of not being a human being	7.00 ± 0.000 (7.0)	6.30 ± 1.457 (6.0)	U=79.0, p=.575 r=-0.038
P1D	Identity confusion (patient thinks s/he is someone else)	8.00 ± 0.000 (8.0)	6.29 ± 1.454 (6.0)	U=27.0, p=.174 r=-0.011
P1D	Nihilistic ideas of being dead / dying	7.00 ± 1.414 (7.0)	6.30 ± 1.457 (6.0)	U=170.0, p=.514 r=-0.043
P1D	Demarcation experiences	8.00 ± 0.000 (8.0)	6.29 ± 1.454 (6.0)	U=27.0, p=.174 r=-0.090
P1D	Ideas of observing oneself from a birds-eye perspective	6.00 ± 0.000 (6.0)	6.30± 1.461 (6.0)	U=173.0, p=.535 r=-0.041
P1D	Exaggerated ideas of guilt	6.07 ± 1.668 (6.0)	6.32 ± 1.442 (6.0)	U=1546.0, p=.739 r=-0.022
P1D	Ideas of jealousy	5.75 ± 2.500 (7.0)	6.31 ± 1.437 (6.0)	U=451.5, p=.972 r=-0.002
P1D	Erotomanic ideas	6.00 ± 1.414 (6.0)	6.30 ± 1.458 (6.0)	U=189.0, p=.656 r=-0.029
P1E	Ideas of being the center of non-negative attention	6.08 ± 1.412 (6.0)	6.33 ± 1.461 (6.0)	U=2317.0, p=.250 r=-0.075
P1E	Non-paranoid ideas of being especially addressed by random events (e.g., media)	5.75 ± 1.775 (6.0)	6.37 ± 1.404 (6.0)	U=2049.0, p=.140 r=-0.097
P2	General mistrust	5.57 ± 1.697 (6.0)	6.35 ± 1.439 (6.0)	U=1105.0, p=.076 r=-0.117
P2	Mistrust of friends	5.87 ± 0.991 (6.0)	6.32 ± 1.468 (6.0)	U=638.0, p=.155 r=-0.093
P2	Paranoid ideas of reference (gazes of passers-by)	6.23 ± 1.581 (6.0)	6.33 ± 1.415 (6.0)	U=4814.5, p=.686 r=-0.027
P2	Paranoid ideas of reference involving friends / family	6.30 ± 1.160 (6.0)	6.30 ± 1.469 (6.0)	U=1042.0, p=.736 r=-0.022

SIPS No.	Content	With idea (mean±SD; (median))	Without idea (mean±SD; (median))	Statistics
P2	Ideas that others wish the patient ill	6.00 ± 0.000 (6.0)	6.30 ± 1.458 (6.0)	U=87.0, p=.662 r=-0.029
P2	Ideas that others would exploit the patient	6.25 ± 0.957 (6.5)	6.30 ± 1.463 (6.0)	U=423.0, p=.799 r=-0.017
P2	Increased vigilance due to feeling unsafe	5.50 ± 1.690 (5.5)	6.33 ± 1.442 (6.0)	U=630.5, p=.144 r=-0.096
P2	Ideas of being threatened/observed by supernatural / invisible beings	6.00 ± 1.155 (6.0)	6.31 ± 1.464 (6.0)	U=623.0, p=.334 r=-0.064
P2	Ideas of being observed anonymously (e.g. by cameras, internet etc.)	7.00 ± 0.000 (7.0)	6.30 ± 1.457 (6.0)	U=79.0, p=.575 r=-0.037
P2	Ideas of being under surveillance (not solely observation)	6.60 ± 0.894 (7.0)	6.30 ± 1.465 (6.0)	U=499.0, p=.635 r=-0.031
P2	Ideas of persecution	6.22 ± 1.202 (6.0)	6.30 ± 1.466 (6.0)	U=941.5, p=.747 r=-0.021
P2	Ideas of being excluded	6.50 ± 1.378 (6.5)	6.30 ± 1.459 (6.0)	U=648.0, p=.849 r=-0.012
P2	Ideas that others intend to harm the patient (not physically)	6.17 ± 1.537 (6.0)	6.32 ± 1.446 6.0)	U=2848.5, p=.773 r=-0.190
P2	Ideas that others intend to poison the patient	5.00 ± 0.000 (5.0)	6.36 ± 1.397 (6.0)	U=75.0, p=.092 r=-0.111
P2	Ideas that others intend to physically harm the patient	6.00 ± 1.443 (6.0)	6.34 ± 1.455 (6.0)	U=2246.0, p=.268 r=-0.073
P2	Ideas that supernatural beings intend to harm the patient	6.00 ± 0.000 (6.0)	6.30 ± 1.458 (6.0)	U=87.0, p=.662 r=-0.029
P2	Ideas of being at risk of falling victim to terror attacks or similar	7.00 ± 0.000 (7.0)	6.30 ± 1.460 (6.0)	U=157.0, p=.427 r=-0.052
P3	Grandiose ideas with respect to own (natural) abilities	6.20 ± 2.150 (7.0)	6.31 ± 1.422 (6.0)	U=1040.5, p=.731 r=-0.023
P3	Grandiose ideas of becoming famous	6.40 ± 0.894 (7.0)	6.30 ± 1.466 (6.0)	U=564.0, p=.981 r=-0.002
P3	Grandiose ideas of being chosen to fulfil a greater plan (e.g. by God)	6.00 ± 2.000 (6.0)	6.31 ± 1.452 (6.0)	U=306.5, p=.742 r=-0.022
P3	Grandiose ideas of becoming enlightened / a higher being	7.00 ± 0.000 (7.0)	6.30 ± 1.457 (6.0)	U=79.0, p=.575 r=-0.038
P3	Grandiose ideas with respect to own supernatural abilities	5.25 ± 2.217 (6.0)	6.32 ± 1.438 (6.0)	U=321.5, p=.299 r=-0.069
P3	Grandiose ideas of being a god / higher being	6.00 ± 0.000 (6.0)	6.30 ± 1.458 (6.0)	U=87.0, p=.662 r=-.029

Contents highlighted in grey have at least trend significance (p<0.100).

Supplementary Table 12b. Comparisons of social functioning (GF:S) of CHR patients with and without the respective perceptual abnormalities/hallucinations (SIPS-P4; N=232).

SIPS No.	Content	With idea (mean±SD; (median))	Without idea (mean±SD; (median))	Statistics
P4B	Acoustic illusions	7.0 ± 0.000 (7.0)	6.29 ± 1.462 (6.0)	U=234.0, p=.330 r=-0.064
P4B	Hearing sounds made by non-living objects	5.83 ± 1.505 (6.0)	6.34 ± 1.447 (6.0)	U=1537.5, p=.144 r=-0.096
P4B	Hearing sounds made by living beings (humans, animals)	6.00 ± 1.852 (6.5)	6.31 ± 1.443 (6.0)	U=845.0, p=.781 r=-0.018
P4B	Audible thoughts (not by others)	6.00 ± 0.000 (6.0)	6.30 ± 1.458 (6.0)	U=87.0, p=.662 r=-0.029
P4B	Hearing one's own name being called	6.43 ± 1.604 (6.5)	6.29 ± 1.448 (6.0)	U=1396.5, p=.585 r=-0.036
P4B	Hearing of unintelligible voices (e.g. murmur)	6.08 ± 1.553 (6.0)	6.32 ± 1.451 (6.0)	U=1298.5, p=.585 r=-0.036
P4B	Hearing of dialoguing voices	6.00 ± 0.000 (6.0)	6.30 ± 1.461 (6.0)	U=173.0, p=.535 r=-0.040
P4B	Hearing of commenting voices	6.50 ± 1.378 (6.5)	6.30 ± 1.459 (6.0)	U=648.0, p=.849 r=-0.012
P4B	Hearing of imperative voices	7.00 ± 1.155 (7.0)	6.28 ± 1.460 (6.0)	U=555.0, p=.172 r=-0.090
P4B	Hearing of insulting voices	6.44 ± 1.130 (6.0)	6.30 ± 1.468 (6.0)	U=994.0, p=.961 r=-0.003
P4	Hearing of God's voice	5.00 ± 0.000 (5.0)	6.31 ± 1.455 (6.0)	U=38.0, p=.243 r=-0.015
P4C	Sensing a presence	6.21 ± 1.424 (6.5)	6.31 ± 1.460 (6.0)	U=1454.0, p=.761 r=-0.020
P4C	Sensing the presence of deceased persons	6.00 ± 0.000 (6.0)	6.30 ± 1.458 (6.0)	U=87.0, p=.662 r=-0.030
P4	Seeing moving shadows in the corner of the eye	7.00 ± 1.124 (7.0)	6.24 ± 1.467 (6.0)	U=1546.0, p=.040 r=-0.135
P4C	Visual illusions	6.40 ± 1.517 (7.0)	6.3 ± 1.457 (6.0)	U=531.5, p=.803 r=-0.016
P4C	Illusions of objects moving	7.00 ± 0.000 (7.0)	6.30 ± 1.457 (6.0)	U=79.0, p=.575 r=-0.037
P4C	Dysmorphophobic illusions	6.50 ± 0.707 (6.6)	6.30 ± 1.460 (6.0)	U=222.0, p=.931 r=-0.006
P4C	Indistinct visual hallucinations	6.60 ± 1.342 (6.0)	6.30 ± 1.459 (6.0)	U=525.0, p=.769 r=-0.020
P4C	Distinct visual hallucinations	6.18 ± 1.888 (6.0)	6.31 ± 1.435 (6.0)	U=1202.5, p=.951 r=-0.004
P4C	Seeing a person's shape	6.87 ± 0.991 (7.0)	6.28 ± 1.466 (6.0)	U=679.0, p=.232 r=-0.078
P4C	Confusion of persons	7.00 ± 0.000 (7.0)	6.30 ± 1.457 (6.0)	U=79.0, p=.575 r=-0.037

SIPS No.	Content	With idea (mean±SD; (median))	Without idea (mean±SD; (median))	Statistics
P4D	Sense of being touched	6.40 ± 1.673 (6.0)	6.30 ± 1.454 (6.0)	U=533.5, p=.814 r=-0.015
P4D	Sense of changed body functions	5.33 ± 1.000 (5.50)	6.33 ± 1.429 (6.0)	U=486.0, p=.224 r=-0.080
P4D	Non-painful bodily sensation	6.19 ± 1.721 (6.0)	6.31 ± 1.430 (6.0)	U=2099.0, p=.683 r=-0.027
P4D	Painful bodily sensation	5.00 ± 1.773 (6.0)	6.35 ± 1.425 (6.0)	U=500.5, p=.029 r=-0.143
P4D	Sense of being infested by parasites	7.00 ± 0.000 (7.0)	6.30 ± 1.457 (6.0)	U=79.0, p=.575 r=-0.037
P4D	Sensing normally non-sensible body functions (e.g. blood flow)	7.00 ± 0.000 (7.0)	6.30 ± 1.457 (6.0)	U=79.0, p=.575 r=-0.037
P4E	Olfactoric hallucinations	6.60 ± 0.548 (7.0)	6.30 ± 1.468 (6.0)	U=515.0, p=.716 r=-0.024
P4	Gustatoric hallucinations	7.00 ± 0.000 (7.0)	6.30 ± 1.457 (6.0)	U=79.0, p=.575 r=-0.037

Contents highlighted in grey have at least trend significance (p<0.100).

Supplementary Table 12c. Comparisons of social functioning (GF:S) of CHR patients with and without the respective sign of disorganized communication (SIPS-P5; N=232).

SIPS No.	Content	With idea (mean±SD; (median))	Without idea (mean±SD; (median))	Statistics
P5 3	Communication is vague	4.50 ± 0.707 (4.6)	6.32 ± 1.451 (6.0)	U=55.0, p=.058 r=-0.125
P5	Poverty of speech	4.33 ± 2.082 (5.0)	6.33 ± 1.433 (6.0)	U=123.5, p=.050 r=-0.128
P5	Neologisms	4.00 ± 2.828 (4.0)	6.32 ± 1.433 (6.0)	U=87.5, p=.121 r=-0.102
P5	Extremely short, non-elaborative speech	7.00 ± 0.000 (7.0)	6.30 ± 1.457 (6.0)	U=79.0, p=.575 r=-0.037
P5	Losing the thread of thoughts (self-experienced)	5.47 ± 1.767 (5.0)	6.36 ± 1.417 (6.0)	U=1132.0, p=.043 r=-0.133
P5	Losing the thread of thoughts (observed by others)	5.61 ± 1.650 (6.0)	6.36 ± 1.426 (6.0)	U=1410.0, p=.053 r=-0.127
P5	Derailment (self-experienced)	8.00 ± 0.000 (8.0)	6.29 ± 1.454 (6.0)	U=27.0, p=.174 r=-0.089
P5	Derailment (observed by others)	6.14 ± 1.676 (6.0)	6.31 ± 1.451 (6.0)	U=772.5, p=.930 r=-0.006
P5	Paralogia / alogia	7.00 ± 0.000 (7.0)	6.30 ± 1.457 (6.0)	U=79.0, p=.575 r=-0.037
P5	Tangentiality (observed by others)	5.14 ± 1.864 (5.0)	6.34 ± 1.430 (6.0)	U=466.0, p=.059 r=-0.124
P5	Thought blockage by intrusion (self-experienced)	7.00 ± 0.000 (7.0)	6.30 ± 1.457 (6.0)	U=79.0, p=.575 r=-0.037
P5	Thought blockage by intrusion (observed by others)	7.50 ± 0.707 (7.5)	6.29 ± 1.456 (6.0)	U=105.0, p=.174 r=-0.089
P5	Thought intrusion (observed by others)	7.00 ± 0.000 (7.0)	6.30 ± 1.457 (6.0)	U=79.0, p=.575 r=-0.037
P5	Circumstantial speech	4.80 ± 1.483 (5.0)	6.33 ± 1.440 (6.0)	U=245.0, p=.026 r=-0.147
P5	Restricted / stereotyped thinking (observed by others)	7.00 ± 0.000 (7.0)	6.30 ± 1.457 (6.0)	U=79.0, p=.575 r=-0.037
P5	Stilted or pedantic speech	4.00 ± 1.414 (4.0)	6.32 ± 1.442 (6.0)	U=45.5, p=.045 r=-0.132
P5	Use of inadequate words	6.00 ± 0.000 (6.0)	6.30 ± 1.458 (6.0)	U=87.0, p=.662 r=-0.029

Contents highlighted in grey have at least trend significance (p<0.100).

Supplementary Table 13a. Comparisons of role functioning (GF:R) of CHR patients with and without the respective attenuated and transient delusional idea (SIPS-P1, SIPS-P2, SIPS-P3; N=232).

SIPS No.	Content	With idea (mean±SD; (median))	Without idea (mean±SD; (median))	Statistics
P1B	Thought insertion	24.20 ± 7.376 (21.0)	23.20 ± 5.202 (22.0)	U=739.0, p=.069 r=-0.119
P1B	Thought withdrawal	23.50 ± 6.364 (21.0)	23.24 ± 5.303 (22.0)	U=177.0, p=.568 r=-0.038
P1B	Audible thoughts (by others)	20.42 ± 4.641 (19.0)	23.40 ± 5.296 (22.0)	U=1225.6, p=.671 r=-0.028
P1B	Experiences of mind being read	20.41 ± 2.671 (20.0)	23.47 ± 5.391 (22.0)	U=1548.0, p=.286 r=-0.070
P1B	Thought broadcasting	22.29 ± 6.473 (19.0)	23.27 ± 5.271 (22.0)	U=676.0, p=.516 r=-0.043
P1B	Experience of being controlled by external forces	20.80 ± 1.643 (20.0)	23.30 ± 5.338 (22.0)	U=521.0, p=.750 r=-0.021
P1E	Ideas that strangers know something about patient	19.50 ± 0.707 (19.5)	23.27 ± 5.308 (22.0)	U=117.5, p=.226 r=-0.080
P1C	Ideas that own thoughts could become real	20.00 ± 0.000 (20.0)	23.26 ± 5.304 (22.0)	U=114.5, p=.988 r=-0.009
P1C	Ideas that own actions would influence the surrounding	22.00 ± 0.000 (22.0)	23.25 ± 5.307 (22.0)	U=62.5, p=.421 r=-0.053
P1C	Belief in supernatural phenomena (ghosts, telepathy, afterlife, power of the universe etc.)	25.38 ± 6.948 (23.5)	23.17 ± 5.232 (22.0)	U=806.0, p=.623 r=-0.032
P1C	Numbers have special meaning	21.67 ± 5.686 (20.0)	23.26 ± 5.302 (22.0)	U=241.5, p=.369 r=-0.059
P1C	Ideas that positive thoughts might cause bad things	18.00 ± 0.000 (18.0)	23.26 ± 5.297 (22.0)	U=20.0, p=.147 r=-0.096
P1C	Ideas of being directly affected by other persons feelings/actions	18.00 ± 4.243 (18.0)	23.29 ± 5.289 (22.0)	U=81.5, p=.110 r=-0.105
P1C	Belief that everything is connected	21.00 ± 0.000 (21.0)	23.25 ± 5.306 (22.0)	U=62.5, p=.421 r=-0.053
P1C	Belief in conspiracy theories	28.00 ± 0.000 (28.0)	23.22 ± 5.299 (22.0)	U=66.5, p=.457 r=-0.049
P1C	Tendency to see relations between random events	23.50 ± 2.121 (23.5)	23.24 ± 5.318 (22.0)	U=75.0, p=.095 r=-0.110
P1C	Ideas that others take over the patient's self/personality	34.00 ± 0.000 (34.0)	23.19 ± 5.260 (22.0)	U=20.0, p=.147 r=-0.096
P1D	Belief in fate	21.00 ± 0.000 (21.0)	23.25 ± 5.306 (22.0)	U=20.0, p=.147 r=-0.095
P1C	Unusual religious ideas	20.00 ± 0.000 (20.0)	23.26 ± 5.304 (22.0)	U=62.5, p=.421 r=-0.053
P1C	Unusual ideas about the world	25.50 ± 6.364 (25.5)	23.22 ± 5.299 (22.0)	U=176.0, p=561. r=-0.038

SIPS No.	Content	With idea (mean±SD; (median))	Without idea (mean±SD; (median))	Statistics
P1C	Ideas that things in the surrounding have a special meaning	21.00 ± 2.000 (21.0)	23.27 ± 5.322 (22.0)	U=290.5, p=.640 r=0.031
P1D	Unusual and unrealistic ideas about the own body	24.20 ± 1.633 (21.33)	23.29 ± 5.352 (22.0)	U=581.0, p=.543 r=-0.040
P1D	Hypochondriacal ideas	25.07 ± 7.195 (24.50)	23.12 ± 5.151 (22.0)	U=1151.5, p=.117 r=-0.103
P1D	Ideas of being pregnant	19.00 ± 0.000 (19.0)	23.26 ± 5.301 (22.0)	U=114.5, p=.988 r=-0.001
P1D	Nihilistic ideas about own non-existence	22.18 ± 5.247 (20.0)	23.33 ± 5.304 (22.0)	U=1728.0, p=.704 r=-0.025
P1D	Ideas of the existence of another reality / universe	20.50 ± 0.707 (20.5)	23.27 ± 5.313 (22.0)	U=202.5, p=.767 r=-0.019
P1D	Nihilistic ideas about the non-existence of others	20.88 ± 4.076 (20.0)	23.54 ± 5.363 (22.0)	U=2544.0, p=.889 r=-0.009
P1D	Ideas of vanishing from the world	21.00 ± 2.828 (21.0)	23.26 ± 5.312 (22.0)	U=226.0, p=.966 r=-0.003
P1D	Ideas of being part of a movie, computer game etc.	20.67 ± 3.512 (21.0)	23.28 ± 5.313 (22.0)	U=75.0, p=.018 r=-0.155
P1D	Ideas that a part of the soul is separated	20.00 ± 0.000 (20.0)	23.26 ± 5.304 (22.0)	U=62.5, p=.421 r=-0.053
P1D	Ideas of not being a human being	21.00 ± 0.000 (21.0)	23.25 ± 5.306 (22.0)	U=62.5, p=.421 r=-0.053
P1D	Identity confusion (patient thinks s/he is someone else)	34.00 ± 0.000 (34.0)	23.19 ± 5.260 (22.0)	U=20.0, p=.147 r=-0.095
P1D	Nihilistic ideas of being dead / dying	20.00 ± 0.000 (20.0)	23.27 ± 5.311 (22.0)	U=176.0, p=.561 r=-0.038
P1D	Demarcation experiences	15.00 ± 0.000 (15.0)	23.28 ± 5.280 (22.0)	U=20.0, p=.147 r=-0.095
P1D	Ideas of observing oneself from a birds-eye perspective	23.50 ± 4.950 (23.5)	23.24 ± 5.310 (22.0)	U=228.0, p=.983 r=-0.001
P1D	Exaggerated ideas of guilt	21.67 ± 3.619 (22.0)	23.35 ± 5.382 (22.0)	U=1620.5 p=.977 r=-0.002
P1D	Ideas of jealousy	28.25 ± 7.411 (26.5)	23.15 ± 5.232 (22.0)	U=427.0, p=.827 r=-0.014
P1D	Erotomanic ideas	22.50 ± 0.707 (22.5)	23.25 ± 5.319 (22.0)	U=132.0, p=.291 r=-0.069
P1E	Ideas of being the center of non-negative attention	22.38 ± 6.670 (19.5)	23.35 ± 5.107 (22.0)	U=2381.0, p=.349 r=-0.061
P1E	Non-paranoid ideas of being esp. addressed by random events	23.54 ± 5.687 (22.0)	23.21 ± 5.263 (22.0)	U=2118.5, p=.217 r=-0.081
P2	General mistrust	22.93 ± 4.428 (22.5)	23.26 ± 5.356 (22.0)	U=1396.0, p=.587 r=-0.036
P2	Mistrust of friends	20.38 ± 3.739 (20.5)	23.34 ± 5.321 (22.0)	U=839.5, p=.758 r=-0.020

SIPS No.	Content	With idea (mean±SD; (median))	Without idea (mean±SD; (median))	Statistics
P2	Paranoid ideas of reference (gazes of passers-by)	21.65 ± 4.760 (21.0)	23.76 ± 5.371 (22.0)	U=4550.5, p=.312 r=-0.066
P2	Paranoid ideas of reference involving friends / family	24.00 ± 6.799 (22.0)	23.21 ± 5.236 (22.0)	U=853.0, p=.208 r=-0.083
P2	Ideas that others wish the patient ill	22.00 ± 0.000 (22.0)	23.25 ± 5.307 (22.0)	U=66.5, p=.457 r=-0.051
P2	Ideas that others would exploit the patient	28.00 ± 7.572 (25.0)	23.16 ± 5.233 (22.0)	U=159.0, p=.023 r=-0.150
P2	Increased vigilance due to feeling unsafe	24.00 ± 7.010 (21.5)	23.21 ± 5.244 (22.0)	U=741.5, p=.399 r=-0.056
P2	Ideas of being threatened/observed by supernatural / invisible beings	21.00 ± 4.655 (20.0)	23.31 ± 5.309 (22.0)	U=687.0, p=.559 r=-0.038
P2	Ideas of being observed anonymously (e.g. by cameras, internet etc.)	22.00 ± 0.000 (22.0)	23.25 ± 5.307 (22.0)	U=114.5, p=.988 r=-0.001
P2	Ideas of being under surveillance (not solely observation)	27.00 ± 8.062 (26.0)	23.16 ± 5.216 (22.0)	U=364.0, p=.163 r=-0.091
P2	Ideas of persecution	23.33 ± 5.657 (23.0)	23.24 ± 5.295 (22.0)	U=989.0, p=.940 r=-0.005
P2	Ideas of being excluded	22.67 ± 2.338 (22.0)	23.26 ± 5.355 (22.0)	U=584.0, p=.555 r=-0.039
P2	Ideas that others intend to harm the patient (not physically)	24.76 ± 5.944 (23.0)	23.02 ± 5.177 (21.0)	U=2399.5, p=.102 r=-0.108
P2	Ideas that others intend to poison the patient	25.00 ± 2.828 (25.0)	23.23 ± 5.314 (22.0)	U=100.5, p=.163 r=-0.092
P2	Ideas that others intend to physically harm the patient	22.88 ± 5.681 (21.0)	23.29 ± 5.261 (22.0)	U=2548.0, p=.899 r=-0.008
P2	Ideas that supernatural beings intend to harm the patient	19.00 ± 0.000 (19.0)	23.26 ± 5.301 (22.0)	U=114.5, p=.988 r=-0.001
P2	Ideas of being at risk of falling victim to terror attacks or similar	29.50 ± 0.707 (29.5)	23.19 ± 5.287 (22.0)	U=183.5, p=.617 r=-0.007
P3	Grandiose ideas with respect to own (natural) abilities	24.30 ± 6.848 (23.5)	23.19 ± 5.231 (22.0)	U=1039.5, p=.730 r=-0.023
P3	Grandiose ideas of becoming famous	24.20 ± 3.633 (23.0)	23.22 ± 5.331 (22.0)	U=422.5, p=.320 r=-0.065
P3	Grandiose ideas of being chosen to fulfil a greater plan (e.g. by God)	19.67 ± 0.577 (20.0)	23.29 ± 5.315 (22.0)	U=158.0, p=.102 r=-0.107
P3	Grandiose ideas of becoming enlightened / a higher being	24.00 ± 0.000 (24.0)	23.24 ± 5.308 (22.0)	U=114.5, p=.988 r=-0.001
P3	Grandiose ideas with respect to own supernatural abilities	26.50 ± 7.141 (28.5)	23.18 ± 5.262 (22.0)	U=397.5, p=.655 r=-0.030
P3	Grandiose ideas of being a god / higher being	17.00 ± 0.000 (17.0)	23.27 ± 5.292 (22.0)	U=2.0, p=.085 r=-0.113

Contents highlighted in grey have at least trend significance (p<0.100).

Supplementary Table 13b. Comparisons of role functioning (GF:R) of CHR patients with and without the respective perceptual abnormalities/hallucinations (SIPS-P4; N=232).

SIPS No.	Content	With idea (mean±SD; (median))	Without idea (mean±SD; (median))	Statistics
P4B	Acoustic illusions	30.67 ± 4.163 (32.0)	23.14 ± 5.247 (22.0)	U=236.0, p=.346 r=-0.062
P4B	Hearing sounds made by non-living objects	22.11 ± 4.689 (21.0)	23.34 ± 5.343 (22.0)	U=1877.0, p=.855 r=-0.012
P4B	Hearing sounds made by living beings (humans, animals)	23.25 ± 6.182 (22.0)	23.24 ± 5.278 (22.0)	U=752.0, p=.432 r=-0.052
P4B	Audible thoughts (not by others)	18.00 ± 0.000 (18.0)	23.26 ± 5.297 (22.0)	U=66.5, p=.457 r=-0.049
P4B	Hearing one's own name being called	20.93 ± 4.763 (19.0)	23.39 ± 5.304 (22.0)	U=1400.0, p=.598 r=-0.035
P4B	Hearing of unintelligible voices (e.g. murmur)	22.69 ± 5.202 (22.0)	23.27 ± 5.312 (22.0)	U=1107.5, p=.171 r=-0.091
P4B	Hearing of dialoguing voices	27.00 ± 12.728 (27.0)	23.21 ± 5.241 (22.0)	U=226.0, p=.966 r=-0.003
P4B	Hearing of commenting voices	21.33 ± 2.582 (21.5)	23.29 ± 5.344 (22.0)	U=634.5, p=.785 r=-0.018
P4B	Hearing of imperative voices	22.0 ± 5.538 (20.0)	23.14 ± 5.247 (22.0)	U=765.0, p=.898 r=-0.008
P4B	Hearing of insulting voices	23.22 ± 6.888 (21.0)	23.24 ± 5.242 (22.0)	U=820.5, p=.345 r=-0.062
P4	Hearing of God's voice	16.0 ± 0.000 (16.0)	23.27 ± 5.286 (22.0)	U=20.0, p=.147 r=-0.095
P4C	Sensing a presence	20.93 ± 4.698 (20.0)	23.39 ± 5.308 (22.0)	U=1225.0, p=.208 r=-0.083
P4C	Sensing the presence of deceased persons	27.00 ± 0.000 (27.0)	23.23 ± 5.302 (22.0)	U=20.0, p=.147 r=-0.095
P4	Seeing moving shadows in the corner of the eye	21.20 ± 3.792 (20.5)	23.43 ± 5.384 (22.0)	U=1896.0, p=.427 r=-0.052
P4C	Visual illusions	22.20 ± 6.723 (20.0)	23.26 ± 5.277 (22.0)	U=518.0, p=734 r=-0.022
P4C	Illusions of objects moving	25.00 ± 0.000 (25.0)	23.23 ± 5.307 (22.0)	U=114.5, p=.988 r=-0.001
P4C	Dysmorphophobic illusions	23.0 ± 2.82 (23.0)	23.24 ± 5.316 (22.0)	U=133.5, p=.299 r=-0.068
P4C	Indistinct visual hallucinations	19.60 ± 0.548 (20.0)	23.32 ± 5.326 (22.0)	U=454.0, p=.437 r=-0.051
P4C	Distinct visual hallucinations	22.18 ± 4.020 (21.0)	23.29 ± 5.345 (22.0)	U=1107.0, p=.611 r=-0.033
P4C	Seeing a person's shape	21.88 ± 4.224 (21.0)	23.29 ± 5.332 (22.0)	U=739.5, p=.393 r=-0.056
P4C	Confusion of persons	30.00 ± 0.000 (30.0)	23.10 ± 5.289 (22.0)	U=20.0, p=.147 r=-0.095

SIPS No.	Content	With idea (mean±SD; (median))	Without idea (mean±SD; (median))	Statistics
P4D	Sense of being touched	18.80 ± 1.304 (19.0)	23.24 ± 5.310 (22.0)	U=543.0, p=.867 r=-0.011
P4D	Sense of changed body functions	25.5 ± 6.979 (24.0)	23.18 ± 5.252 (22.0)	U=669.5, p=.957 r=-0.003
P4D	Non-painful bodily sensation	23.62 ± 4.674 (24.0)	23.20 ± 5.363 (22.0)	U=2056.5, p=.581 r=-0.036
P4D	Painful bodily sensation	20.87 ± 3.603 (20.5)	23.33 ± 5.333 (22.0)	U=859.0, p=.840 r=-0.013
P4D	Sense of being infested by parasites	21.00 ± 0.000 (21.0)	23.25 ± 5.306 (22.0)	U=62.5, p=.421 r=-0.052
P4D	Sensing normally non-sensible body functions (e.g. blood flow)	24.00 ± 0.000 (24.0)	23.24 ± 5.308 (22.0)	U=114.5, p=.988 r=-0.001
P4E	Olfactoric hallucinations	20.40 ± 1.673 (20.0)	23.30 ± 5.333 (22.0)	U=458.5, p=.455 r=-0.050
P4	Gustatoric hallucinations	21.00 ± 0.000 (21.0)	23.25 ± 5.306 (22.0)	U=114.5, p=.988 r=-0.001

Contents highlighted in grey have at least trend significance (p<0.100).

Supplementary Table 13c. Comparisons of role functioning (GF:R) of CHR patients with and without the respective sign of disorganized communication (SIPS-P5; N=232).

SIPS No.	Content	With idea (mean±SD; (median))	Without idea (mean±SD; (median))	Statistics
P5 3	Communication is vague	21.00 ± 1.414 (21.0)	23.26 ± 5.315 (22.0)	U=182.0, p=.605 r=-0.034
P5	Poverty of speech	32.00 ± 11.269 (38.0)	23.13 ± 5.127 (22.0)	U=308.5, p=.758 r=-0.020
P5	Neologisms	28.00 ± 14.142 (28.0)	23.20 ± 5.218 (22.0)	U=36.0, p=.0377 r=-0.066
P5	Extremely short, non-elaborative speech	39.00 ± 0.000 (39.0)	23.17 ± 5.205 (22.0)	U=114.5, p=.988 r=-0.065
P5	Losing the thread of thoughts (self-experienced)	24.20 ± 5.157 (21.0)	23.18 ± 5.157 (22.0)	U=1548.5, p=.749 r=-0.021
P5	Losing the thread of thoughts (observed by others)	25.22 ± 7.765 (22.0)	23.07 ± 5.025 (22.0)	U=1718.5, p=.440 r=-0.051
P5	Derailment (self-experienced)	23.00 ± 0.000 (23.0)	23.24 ± 5.308 (22.0)	U=62.5, p=.421 r=-0.028
P5	Derailment (observed by others)	25.43 ± 6.680 (22.0)	23.17 ± 5.252 (22.0)	U=736.0, p=.764 r=-0.020
P5	Paralogia / alogia	26.00 ± 0.000 (26.0)	23.23 ± 5.305 (22.0)	U=62.5, p=.421 r=-0.053
P5	Tangentiality (observed by others)	24.14 ± 7.515 (23.0)	23.21 ± 5.234 (22.0)	U=504.0, p=.099 r=-0.108
P5	Thought blockage by intrusion (self-experienced)	19.00 ± 0.000 (19.0)	23.26 ± 5.301 (22.0)	U=114.5, p=.988 r=-0.001
P5	Thought blockage by intrusion (observed by others)	24.50 ± 7.778 (24.5)	23.23 ± 5.293 (22.0)	U=133.5, p=.299 r=-0.069
P5	Thought intrusion (observed by others)	19.00 ± 0.000 (19.0)	23.26 ± 5.301 (22.0)	U=114.5, p=.988 r=-0.001
P5	Circumstantial speech	23.80 ± 6.458 (22.0)	23.23 ± 5.285 (22.0)	U=138.0, p=.003 r=-0.193
P5	Restricted / stereotyped thinking (observed by others)	22.00 ± 0.000 (22.0)	23.25 ± 5.307 (22.0)	U=66.5, p=.457 r=-0.049
P5	Stilted or pedantic speech	19.50 ± 0.707 (19.5)	23.27 ± 5.308 (22.0)	U=36.0, p=.037 r=-0.137
P5	Use of inadequate words	22.00 ± 0.000 (22.0)	23.25 ± 5.307 (22.0)	U=66.5, p=.457 r=-0.049

Contents highlighted in grey have at least trend significance (p<0.100).

Supplementary Table 14. Comparison of the prevalence rates (n (%)) of delusional and hallucinatory contents reported for the APS subsample of the NAPLS-2 study (2; N=444) and an US undergraduate sample divided by psychometric schizotypy (3; N=153) assessed with the Content of Attenuated Positive Symptoms (CAPS) codebook (4), and our sample (N=232; of these 158 with APS/BIPS).

SIPS No.	CAPS content	Marshall et al. (N=444)	Trask et al.: Positive schizotypy[c] (N=49)	Trask et al.: Negative schizotypy[d] (N=63)	Trask et al.: Controls[e] (N=41)	Present study (N=232)	n (%[a], %[b])
P1	Perplexed by reality	240 (54.1%)	38 (77.6%)	30 (47.6%)	8 (19.5%)	Not analyzed for lack of a distinct idea	
P1	Overvalued beliefs (e.g., objects having special meaning)	232 (52.3%)	33 (67.3%)	24 (38.1%)	9 (22.0%)	Not analyzed if shared by other members of the same culture or rated as part of magical thinking:<br>Tendency to see relations between random events<br>Belief that everything is connected<br>Numbers have special meaning<br>Ideas that positive thoughts might cause bad things<br>Ideas that own thoughts could become real<br>Ideas that own actions would influence the surrounding	 2 (0.9%, 1.3%)<br>1 (0.4%, 0.6%)<br>3 (1.3%, 1.9%)<br>1 (0.4%, 0.6%)<br>1 (0.4%, 0.6%)<br>1 (0.4%, 0.6%)
P1	Loss of control of content of thoughts	109 (24.6%)	9 (18.4%)	2 (3.2%)	1 (2.4%)	Indistinct description	
P1	Supernatural (e.g., fairies, ghosts, forces)	100 (22.5%)	5 (10.2%)	3 (4.8%)	1 (2.4%)	Belief in supernatural phenomena (ghosts, telepathy, afterlife, etc.)	8 (3.4%, 5.1%)
						Ideas of the existence of another reality / universe	2 (0.9%, 1.3%)
						Belief in fate	1 (0.4%, 0.6%)
						Belief in conspiracy theories	1 (0.4%, 0.6%)
P1	Thought interference	99 (22.3%)	8 (16.3%)	3 (4.8%)	0 (0.00%)	Not analyzed when immediately self-recognized as a disturbance of own	10 (4.3%, 6.3%)

SIPS No.	CAPS content	Marshall et al. (N=444)	Trask et al.: Positive schizotypy[c] (N=49)	Trask et al.: Negative schizotypy[d] (N=63)	Trask et al.: Controls[e] (N=41)	Present study (N=232)	n (%[a], %[b])
						mental processes or rated as part of thought insertion	
P1	Unusual violent thoughts	85 (19.1%)	Not reported			No separate category	
P1	Reading of thoughts	96 (21.6%)	20 (40.8%)	12 (19.0%)	2 (4.9%)	Experiences of mind being read	17 (7.3%, 10.8%)
						Audible thoughts (heard by others)	12 (5.2%, 7.6%)
						Thought broadcasting	7 (3.0%, 4.4%)
						Ideas that strangers know something about patient	2 (0.9%, 1.3%)
P1	Altered familiar people or surroundings	82 (18.5%)	14 (28.6%)	11 (17.5%)	2 (4.9%)	Not analyzed if only derealization experiences or rated as a nihilistic idea	
P1	Special attention from others	71 (16.0%)	13 (26.5%)	10 (15.9%)	2 (4.9%)	Ideas of being the centre of non-negative attention	26 (11.2%, 16.5%)
P1	Unusual religious thoughts	54 (12.2%)	10 (20.4%)	1 (1.6%)	3 (7.3%)	Unusual religious ideas	1 (0.4%, 0.6%)
P1	Sense of time	45 (10.1%)	10 (20.4%)	12 (19.0%)	4 (9.8%)	Not analyzed as not a distinct idea about actual changes in time	
P1	Negative thoughts regarding self	70 (15.8%)	2 (4.1%)	0 (0.00%)	1 (2.4%)	Indistinct description	
P1	Somatic concerns	61 (13.7%)	22 (44.9%)	19 (30.2%)	9 (22.0%)	Unusual and unrealistic ideas about the own body	6 (2.6%, 3.8%)
						Hypochondriacal ideas	14 (6.0%, 8.7%)
						Ideas of being pregnant	1 (0.4%, 0.6%)
P1	Electronic communication	44 (9.9%)	9 (18.4%)	1 (1.6%)	0 (0.00%)	Indistinct, partly included in non-paranoid ideas of being especially addressed by random events (e.g., media)	24 (10.3%, 15.2%)

SIPS No.	CAPS content	Marshall et al. (N=444)	Trask et al.: Positive schizotypy[c] (N=49)	Trask et al.: Negative schizotypy[d] (N=63)	Trask et al.: Controls[e] (N=41)	Present study (N=232)	n (%[a], %[b])
P1	Guilt	40 (9.0%)	3 (6.1%)	9 (14.3%)	0 (0.00%)	Exaggerated ideas of guilt	15 (6.5%, 9.5%)
P1	Unusual sexual thoughts	19 (4.3%)	0 (0.00%)	0 (0.00%)	0 (0.00%)	Erotomanic ideas	2 (0.9%, 1.3%)
P1	Nihilistic ideas	52 (11.7%)	7 (14.3%)	5 (7.9%)	1 (2.4%)	Nihilistic ideas about own non-existence	17 (7.3%, 10.8%)
						Nihilistic ideas about the non-existence of others	25 (10.8%, 15.8%)
						Ideas of vanishing from the world	2 (0.9%, 1.3%)
						Nihilistic ideas of being dead / dying	2 (0.9%, 1.3%)
						Ideas of being part of a movie, computer game etc.	3 (1.3%, 1.9%)
						Ideas that a part of the soul is separated	1 (0.4%, 0.6%)
						Ideas of not being a human being	1 (0.4%, 0.6%)
						Identity confusion (patient thinks s/he is someone else)	1 (0.4%, 0.6%)
						Demarcation experiences	1 (0.4%, 0.6%)
						Ideas of observing oneself from a birds-eye perspective	2 (0.9%, 1.3%)
P1	Not reported					Ideas of being directly affected by other persons feelings/actions	2 (0.9%, 1.3%)
P1	Not reported					Ideas that others take over the patient's self/personality	1 (0.4%, 0.6%)
P2	Ideas of being thought about in a negative way	211 (47.5%)	36 (73.5%)	39 (61.9%)	21 (51.2%)	Mainly not analyzed if only vague concerns, or rated as part of Ideas that others wish the patient ill or of Ideas of being excluded	1 (0.4%, 0.6%)<br><br>6 (2.6%, 3.8%)

SIPS No.	CAPS content	Marshall et al. (N=444)	Trask et al.: Positive schizotypy[c] (N=49)	Trask et al.: Negative schizotypy[d] (N=63)	Trask et al.: Controls[e] (N=41)	Present study (N=232)	n (%[a], %[b])
P2	Guardedness towards people	189 (42.6%)	38 (77.6%)	36 (57.1%)	17 (41.5%)	Ideas that others would exploit the patient	4 (1.7%, 2.5%)
	Ideas related to the misuse of personal information	11 (2.5%)	0 (0.00%)	1 (1.6%)	0 (0.00%)	General mistrust	14 (6.0%, 8.9%)
						Mistrust of friends	8 (3.4%, 5.1%)
P2	Hypervigilance of surroundings	110 (24.8%)	21 (42.9%)	15 (23.8%)	8 (19.5%)	Increased vigilance due to feeling unsafe	8 (3.4%, 5.1%)
P2	Ideas of being harmed emotionally	101 (22.8%)	4 (8.2%)	5 (7.9%)	3 (7.3%)	Ideas that others intend to harm the patient (not physically)	29 (12.5%, 18.4%)
P2	Ideas of being harmed physically	144 (32.4%)	3 (6.1%)	3 (4.8%)	2 (4.9%)	Ideas that others intend to physically harm the patient	25 (10.8%, 15.8%)
						Ideas that others intend to poison the patient	2 (0.9%, 1.3%)
						Ideas that supernatural beings intend to harm the patient	1 (0.4%, 0.6%)
						Ideas of being at risk of falling victim to terror attacks or similar	2 (0.9%, 1.3%)
P2	Ideas of being watched	128 (28.8%)	6 (12.2%)	7 (11.1%)	2 (4.9%)	Ideas of being threatened/observed by supernatural / invisible beings	7 (3.0%, 4.4%)
						Ideas of being observed anonymously (e.g., by cameras, internet etc.)	1 (0.4%, 0.6%)
						Ideas of being under surveillance (not solely observation)	5 (2.2%, 3.2%)
						Paranoid ideas of reference (gazes of passers-by)	57 (24.6%, 36.1%)
						Paranoid ideas of reference involving friends / family	10 (4.3%, 6.3%)

SIPS No.	CAPS content	Marshall et al. (N=444)	Trask et al.: Positive schizotypy[c] (N=49)	Trask et al.: Negative schizotypy[d] (N=63)	Trask et al.: Controls[e] (N=41)	Present study (N=232)	n (%[a], %[b])
P2	Ideas of being followed	29 (6.5%)	1 (2.0%)	3 (4.8%)	0 (0.00%)	Ideas of persecution	9 (3.9%, 5.7%)
P2	Ideas regarding an unfaithful partner	19 (4.3%)	12 (24.5%)	10 (15.9%)	3 (7.3%)	Ideas of jealousy (P1)	4 (1.7%, 2.5%)
P3	Skills or abilities or talents (e.g., artistic, athletic)	104 (23.4%)	17 (34.7%)	4 (6.3%)	6 (14.6%)	Grandiose ideas with respect to own (natural) abilities	10 (4.3%, 6.3%)
P3	Intelligence	46 (10.4%)	0 (0.00%)	2 (3.2%)	1 (2.4%)	Not analyzed if mostly private thoughts or rated as part of grandiose ideas with respect to own (natural) abilities	
P3	Status (e.g., being famous or particularly important)	71 (15.6%)	9 (18.4%)	3 (4.8%)	0 (0.00%)	Grandiose ideas of becoming famous	5 (2.2%), 3.2%
P3	Unrealistic goals or plans	48 (10.8%)	5 (10.2%)	2 (3.2%)	0 (0.00%)	Not analyzed if only highly ambitious or rated as part of other grandiose ideas	
P3	Religious content	23 (5.2%)	5 (10.2%)	1 (1.6%)	2 (4.9%)	Grandiose ideas of being chosen to fulfil a greater plan (e.g. by God)	3 (1.3%, 1.9%)
						Grandiose ideas of being a god / higher being	1 (0.4%, 0.6%)
						Grandiose ideas of becoming enlightened / a higher being	1 (0.4%, 0.6%)
P3	Ability to influence or control others or the world	12 (2.7%)	1 (2.0%)	0 (0.00%)	0 (0.00%)	Grandiose ideas with respect to own supernatural abilities	4 (1.7%, 2.5%)
P4	Indistinct noises (e.g., ringing, hissing, buzzing)	156 (35.1%)	33 (67.3%)	25 (39.7%)	13 (31.7%)	Hearing sounds made by non-living objects	18 (7.8%, 11.4%)

SIPS No.	CAPS content	Marshall et al. (N=444)	Trask et al.: Positive schizotypy[c] (N=49)	Trask et al.: Negative schizotypy[d] (N=63)	Trask et al.: Controls[e] (N=41)	Present study (N=232)	n (%[a], %[b])
P4	Distinct noises (e.g., foot-steps, knocking, doors opening)	123 (27.7%)	9 (18.4%)	11 (17.5%)	3 (7.3%)	Hearing sounds made by living beings (humans, animals)	8 (3.4%, 5.1%)
P4	Voices<br><br>*Neutral content*<br>*Negative content*<br>*Positive content*	114 (25.7%)<br><br>*53 (11.9%)*<br>*65 (14.6%)*<br>*13 (2.9%)*	8 (16.3%)<br><br>*5 (10.2%)*<br>*1 (2.0%)*<br>*1 (2.0%)*	0 (0.00%)	1 (2.4%)<br><br>*1 (2.4%)*<br>0 (0.00%)<br>0 (0.00%)	Hearing of dialoguing voices	2 (0.9%, 1.3%)
						Hearing of commenting voices	6 (2.6%, 3.8%)
						Hearing of imperative voices	7 (3.0%, 4.4%)
						Hearing of insulting voices	9 (3.9%, 5.7%)
						Hearing of God's voice	1 (0.4%, 0.6%)
P4	Name being called	101 (22.8%)	7 (14.3%)	2 (3.2%)	2 (4.9%)	Hearing one’s own name being called	14 (6.0%, 8.9%)
P4	Auditory: sensitivity	95 (21.4%)	4 (8.2%)	3 (4.8%)	1 (2.4%)	Not analyzed because only rated with score of 1 on the SIPS	
P4	Mumbling	83 (18.7%)	2 (4.1%)	1 (1.6%)	1 (2.4 %)	Hearing of unintelligible voices (e.g., murmur)	13 (5.6%, 8.2%)
P4	Thoughts being said out loud	29 (6.5%)	12 (24.5%)	0 (0.00%)	2 (4.9%)	Audible thoughts (not heard by others)	1 (0.4%, 0.6%)
P4	Distortions (e.g., hearing spoken words as music)	24 (5.4%)	0 (0.00%)	2 (3.2%)	1 (2.4%)	Acoustic illusions	3 (1.3%, 1.9%)
P4	Visual: sensitivity	61 (13.7%)	6 (12.2%)	10 (15.9%)	4 (9.8%)	Not analyzed because only rated with score of 1 on the SIPS	
P4	Vague figures or shadows	208 (46.9%)	27 (55.1%)	17 (27.0%)	3 (7.3%)	Seeing moving shadows in the corner of the eye	20 (8.6%, 12.7%)
						Seeing a person's shape	8 (3.4%, 5.1%)
						Indistinct visual hallucinations	5 (2.2%, 3.2%)
P4	Spots or floaters	22 (5.0%)	2 (4.1%)	1 (1.6%)	0 (0.00%)	Not analyzed if immediately perceived as a perceptual problem or, if initially	
P4	Geometric shapes	13 (2.9%)	0 (0.00%)	0 (0.00%)	0 (0.00%)		
P4	Flames or fire	4 (0.9%)	0 (0.00%)	0 (0.00%)	0 (0.00%)		

SIPS No.	CAPS content	Marshall et al. (N=444)	Trask et al.: Positive schizotypy[c] (N=49)	Trask et al.: Negative schizotypy[d] (N=63)	Trask et al.: Controls[e] (N=41)	Present study (N=232)	n (%[a], %[b])
P4	Flashes of light	50 (11.3%)	3 (6.1%)	3 (4.8%)	1 (2.4%)	perceived as a real perception, rated as Indistinct visual hallucinations	
P4	Distortions (e.g., walls moving in waves)	75 (16.9%)	11 (22.4%)	6 (9.5%)	1 (2.4%)	Not analyzed if immediately perceived as a perceptual problem; or, if initially perceived as a real perception, rated as Illusions of objects moving	1 (0.4%, 0.6%)
P4	Faces or people	72 (16.2%)	9 (18.4%)	4 (6.3%)	0 (0.00%)	Confusion of persons	1 (0.4%, 0.6%)
						Visual illusions	5 (2.2%, 3.2%)
	Animals	46 (10.4%)	11 (22.4%)	3 (4.8%)	0 (0.00%)	Dysmorphophobic illusions	2 (0.9%, 1.3%)
						Distinct visual hallucinations	11 (4.7%, 7.0%)
P4	Not rated					Sensing a presence	14 (6.0%, 8.9%)
P4	Not rated					Sensing the presence of deceased persons	1 (0.4%, 0.6%)
P4	Something touching the individual	44 (9.9%)	1 (2.0%)	1 (1.6%)	0 (0.00%)	Sense of being touched	5 (2.2%, 3.2%)
P4	Numbness or tingling	40 (9.0%)	12 (24.5%)	4 (6.3%)	1 (2.4%)	Non-painful bodily sensation	21 (9.1%, 13.3%)
P4	Electricity or vibrations	21 (4.7%)	3 (6.1%)	3 (4.8%)	0 (0.00%)		
P4	Burning or coldness	17 (3.8%)	1 (2.0%)	2 (3.2%)	0 (0.00%)		
P4	Bugs crawling	16 (3.6%)	0 (0.00%)	0 (0.00%)	0 (0.00%)	Sense of being infested by parasites	1 (0.4%, 0.6%)
P4	Physical alterations	16 (3.6%)	0 (0.00%)	1 (1.6%)	0 (0.00%)	Sensing normally non-sensible body functions (e.g., blood flow)	1 (0.4%, 0.6%)
						Sense of changed body functions	6 (2.6%, 3.8%)
P4	Aches or pain	16 (3.6%)	13 (26.5%)	6 (9.5%)	4 (9.8%)	Painful bodily sensation	8 (3.4%, 5.1%)
P4	Unpleasant smells	28 (6.3%)	4 (8.2%)	2 (3.2%)	1 (2.4%)	Olfactory hallucinations	5 (2.2%, 3.2%)
P4	Pleasant smells	18 (4.1%)	2 (4.1%)	0 (0.00%)	0 (0.00%)		
P4	Not rated					Gustatory hallucinations	1 (0.4%, 0.6%)

[a] % with respect to the whole sample of the present study (N=232).
[b] % with respect to the subsample with APS/BIPS of the present study (n=158, 68.1%).
[c] Participants were then assigned to the positive group (n = 49) if they scored greater than 1.96 SDs above the mean on either - or a combined 3.00 SDs above the mean on both - the Magical Ideation Scale (5) and/or the Perceptual Aberration Scale (6).
[d] Participants were assigned to the negative group (n=63) if they scored greater than 1.96 SDs above the mean on the Social Anhedonia Scale (7).
[e] Those who scored less than 0.5 SDs above the mean on all three schizotypy scales were assigned to the control group (n=41)
Note: Signs of disorganized communication (SIPS-P5) are not investigated / included in the Content of Attenuated Positive Symptoms Codebook (4).
Not analyzed: Generally, a below-APS-level phenomenon (score 1-2) and, therefore, not analyzed in the present study.
Indistinct: Content might include various types of below-APS-level phenomena and APS, and, therefore, a matching content in our study was not clearly identified.

References

1. 44. Deutsche Gesellschaft für Psychitrie. Psychotherapie und Nervenheilkunde (DGPPN). S3 Praxisleitlinien in Psychiatrie und Psychotherapie. Band 1: Behandlungsleitlinie Schizophrenie. Heidelberg: Steinkopff (2006). p. 288.
2. Marshall C, Denny E, Cadenhead KS, Cannon TD, Cornblatt BA, McGlashan TH, et al. The content of attenuated psychotic symptoms in those at clinical high risk for psychosis. Psychiatry Res (2014) 219:506. doi: 10.1016/j.psychres.2014.06.023
3. Trask CL, Cohn JR, Paxson AM, Hansen GS, Cicero DC. Form and content of attenuated psychotic symptoms in psychometrically assessed positive and negative schizotypy. Early Interv Psychiatry (2020) 14:321. doi: 10.1111/eip.12856
4. Marshall C, Falukozi E, Albertin M, Zhu H, Addington J. The development of the Content of Attenuated Positive Symptoms Codebook for those at clinical high risk of psychosis. Psychosis (2011) 4:191. doi: 10.1080/17522439.2011.626070
5. Eckblad M, Chapman LJ. Magical ideation as an indicator of schizotypy. J Consult Clin Psychol (1983) 51:215.
6. Chapman LJ, Chapman JP, Raulin ML. Body-image aberration in Schizophrenia. J Abnorm Psychol (1978) 87:399.
7. Eckblad M, Chapman LJ, Chapman JP, Mishlove M. The revised social anhedonia scale. Available from L. J. Chapman. Department of Psychology, University of Wisconsin, Madison, WI. (1982).
